# Taming C_60_ fullerene: tuning intramolecular photoinduced electron transfer process with subphthalocyanines†
†Electronic supplementary information (ESI) available. CCDC 1014106. For ESI and crystallographic data in CIF or other electronic format see DOI: 10.1039/c5sc00223k


**DOI:** 10.1039/c5sc00223k

**Published:** 2015-04-16

**Authors:** Marc Rudolf, Olga Trukhina, Josefina Perles, Lai Feng, Takeshi Akasaka, Tomas Torres, Dirk M. Guldi

**Affiliations:** a Department of Chemistry and Pharmacy , Interdisciplinary Center for Molecular Materials (ICMM) , Friedrich-Alexander-Universität Erlangen-Nürnberg , 91058 Erlangen , Germany . Email: dirk.guldi@fau.de; b Department of Organic Chemistry , Autonoma University of Madrid , Cantoblanco , 28049 Madrid , Spain; c IMDEA Nanoscience , Faraday 9 , 28049 Madrid , Spain; d Interdepartamental Research Service (SIDI) , Lab. of High Resolution X-Ray Diffraction of Monocrystals , Autonoma University of Madrid , Cantoblanco , 28049 Madrid , Spain; e Life Science Center of Tsukuba Advanced Research Alliance , University of Tsukuba , 305-8577 Tsukuba , Japan; f College of Physics , Optoelectronics and Energy & Collaborative Innovation Center of Suzhou Nano Science and Technology , Soochow University , 215006 Suzhou , China; g Foundation for Advancement of International Science , 305-0821 Tsukuba , Japan; h Department of Chemistry , Tokyo Gakugei University , 184-8501 Koganei , Japan; i College of Materials Science and Engineering , Huazhong University of Science and Technology , 430074 Wuhan , China

## Abstract

Two subphthalocyanine–C_60_ conjugates have been prepared by means of the 1,3-dipolar cycloaddition reaction of (perfluoro) or hexa(pentylsulfonyl) electron deficient subphthalocyanines to C_60_. Comprehensive assays regarding the electronic features – in the ground and excited state – of the resulting conjugates revealed energy and electron transfer processes upon photoexcitation. Most important is the unambiguous evidence – in terms of time-resolved spectroscopy – of an ultrafast oxidative electron transfer evolving from C_60_ to the photoexcited subphthalocyanines. This is, to the best of our knowledge, the first case of an intramolecular oxidation of C_60_ within electron donor–acceptor conjugates by means of only photoexcitation.

## Abstract 2

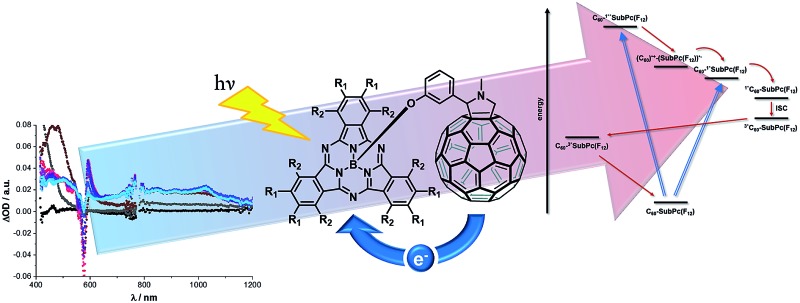
Two subphthalocyanine–C_60_ fullerene electron donor–acceptor conjugates have been prepared from electron deficient subphthalocyanines and C_60_, with evidence of an ultrafast oxidative electron transfer from C_60_ to the subphthalocyanines.

## Introduction

The conversion of solar light into electrical energy remains one of the grand challenges of modern society due to the constantly growing energy demand and resource depletions.^1^ In natural photosynthetic systems,^2^ cascades of energy- and electron-transfer reactions are triggered either directly by photoexcitation or indirectly by energy transfer from light-harvesting antenna systems. In terms of charge separation, the characteristics of the individual electron acceptors and electron donors are decisive to modulate its overall efficiency. In terms of charge recombination, the environment of the photosynthetic reaction center plays a crucial role to slow it down. Therefore, designing, synthesizing, and probing efficient energy capacitors as well as porphyrinoid chromophores featuring unique panchromatic absorptive, redox, and electrical properties is of crucial importance.

To this end, fullerenes – as one of the most explored class of molecular materials – have generated an enormous interest in the field of nanoelectronics. On one hand, their rigid aromatic structure evokes low reorganization energies in electron transfer reactions and, on the other hand, their extended π-conjugation affords efficient charge stabilization.^3^ Unique architectural flexibility and chemical versatility of fullerenes allow the fine-tuning of their properties by, for example, covalent modification of their exterior^4^ or incorporation of molecular guests into their interior.^5^ In light of the aforementioned, a myriad of electron donor–acceptor conjugates/hybrids have been designed featuring porphyrins,^6^ (sub)phthalocyanines,^7^,^8^ perylene-diimides,^9^ and other chromophores as light-harvesting electron donors and fullerenes as electron acceptors. Notably, reports in which fullerenes are employed as electron donors remain, however, scarce.

Oxidation of fullerenes requires harsh conditions and, thus, occurs only by treating them with, for example, strong oxidizing agents^10^ or upon photo- or electron-induced ionization in the presence of electron transferring photosensitizers.^11^ The first example of *intermolecular* electron-transfer oxidation of fullerenes was reported by Fukuzumi *et al.* in the presence of *p*-benzoquinone and radical ion pair stabilizing scandium triflate Sc(OTf)_3_.^12^ Only a single case of *intramolecular* oxidation of fullerenes has been reported to date. In a fullerene–trinitrofluorenone conjugate, photoexcitation and the presence of Sc(OTf)_3_ leads, indeed, to the formation of oxidized fullerenes, whereas only the triplet excited state of the fullerene was observed in the absence of Sc(OTf)_3_.^13^ No successful *intramolecular* oxidation of fullerenes by means of simple photoexcitation has ever been reported.

In stark contrast, recent studies have demonstrated that endohedral metallofullerenes function as electron donors in electron donor–acceptor conjugates.^14^ Thus, linking fullerenes to strong electron acceptors, which provide the means of charge stabilization within their extended π-system, could assist in shedding light onto the rare electron donating features even of empty fullerenes. This could open up new ways of using fullerenes in novel photoelectrochemical water splitting devices, which operate under oxidative conditions, novel solar cells, which feature larger open circuit voltages, or towards optoelectronic devices integrating novel n-type semiconductors.

In this connection, 14 π-electron aromatic subphthalocyanines (SubPc) represent ideal candidates with an extended conjugation and a cone-shaped geometry.15a,b Owing to their intense absorption in the visible region of the solar spectrum – complementary to that of fullerenes – and high optical and thermal stabilities, they have evolved as perfect light-harvesting building blocks.15*c* Low reorganization energies in electron transfer reactions and low reduction potentials of SubPcs, which bear electron-withdrawing substituents,15*d* render them ideal electron acceptors when linked to fullerenes.

**Scheme 1:**
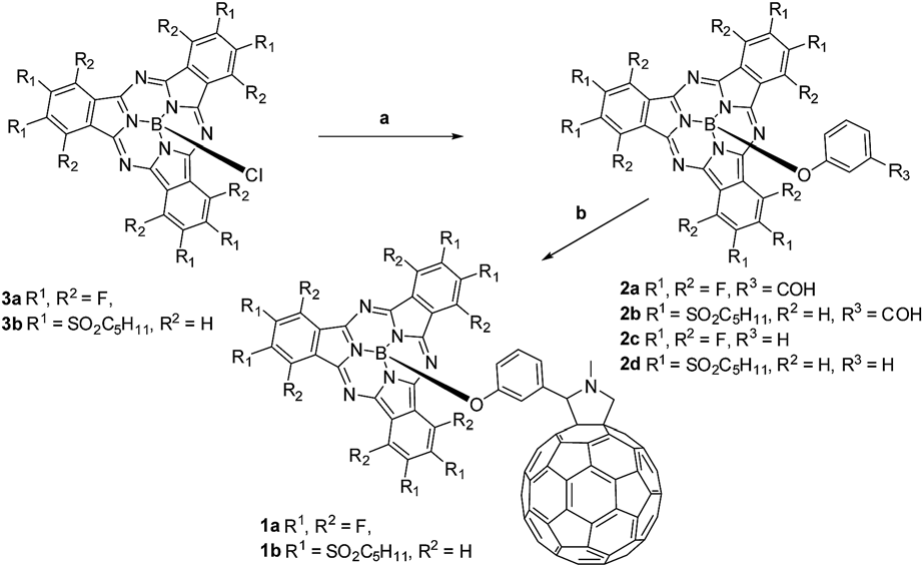
Synthesis of SubPc–C_60_ conjugates **1a,b**. Conditions: (a) AgOTf, 3-hydroxybenzaldehyde/phenol, toluene; (b) *N*-methylglycine, C_60_, toluene, reflux.

Herein, we report on the synthesis as well as the electrochemical and photophysical features of conjugates **1a**,**b** (Scheme 1) that is, C_60_ and electron accepting SubPcs with electron-withdrawing fluorine or pentylsulfonyl substituents on their periphery. This is meant to assist in realizing a game changer for advanced solar energy conversion schemes, namely photoinduced oxidation of empty fullerenes under small reorganization energies.

## Results and discussion

Electron-deficient chloro-(alkylsulfonyl) SubPcs are known as the least reactive SubPcs in terms of axial substitution of the chlorine atoms. In fact, examples of conjugates involving (alkylsulfonyl) SubPcs are limited to only a single case, namely a hexa(octylsulfonyl) SubPc–ferrocene conjugate reported by our group.15*d* In the present work, (perfluoro) and hexa(pentylsulfonyl) derivatives of (formylphenoxy) substituted SubPc precursors **2a**,**b** and (phenoxy) SubPc references **2c**,**d** were successfully synthesized through triflate-substituted intermediates, generated by treating (chloro) SubPcs **3a**,**b** with AgOTf (Scheme 1) according to a recently developed procedure.^16^ Corresponding triflate-containing SubPc intermediates were further subjected to *in situ* substitution of the triflyl group by 3-hydroxybenzaldehyde or phenol in toluene at 100 °C affording the desired (formylphenoxy) (**2a**,**b**) or (phenoxy) (**2c**,**d**) substituted SubPcs in good yields. Transformation of (chloro)-hexa(pentylsulfonyl) SubPcs **3b** into the corresponding phenoxy-derivative **2d** enabled its single crystal and, in turn, structural analysis by means of X-ray diffraction (Fig. S45 and S46 in the ESI†).

Conjugates **1a**,**b** were obtained by the 1,3-dipolar cycloaddition of azomethine ylides generated *in situ* in a reaction of (3-formylphenoxy) SubPcs **2a**,**b** and *N*-methylglycine, to C_60_.17*c* TLC and HPLC analyses (Fig. S1 and S2†) inform that the reaction time for forming **1b** is only 2 h. This reflects the perturbation stemming from the six electron deficient substituents on the electronic properties of **2b** and its corresponding intermediates. In contrast, perfluorinated SubPc **2a** reacts in 8 h and reveals, as such, a reactivity similar to that seen for electron-donating SubPcs in Prato protocols.8a,c–e Subsequent column chromatography on silica gel, followed by size exclusion separation on Bio-Beads, yielded pure **1a** in 63% and moderate amounts, namely around 6% of bis-adducts. Efficient purification of **1b** was, nevertheless, hampered by its degradation on silica gel and formation of decomposition products with similar polarities. Performing column chromatography on cyanopropyldichlorosilyl-modified silica gel as a solid support^18^ allowed the successful separation of the conjugate in somewhat lower yields of 24% followed by the final separation of bis-adducts by GPC.

All compounds were fully characterized by ^1^H and ^13^C NMR, MALDI-MS, UV-vis, and FTIR spectroscopy (Fig. S3–S38†). **1a** and **1b** are obtained as a mixture of two diastereoisomers as is known for Prato addition reactions to C_60_.17*a*

Being inseparable by conventional column chromatography on silica gel or by HPLC on a Buckyprep column (Fig. S1 and S2†), their presence is clearly manifested by the broadening in the ^1^H NMR spectra (Fig. S3 and S9†). The proton signals of the phenyl spacer in **1a** and **1b** are assigned on the basis of the coupling patterns observed in ^1^H COSY NMR (Fig. S4 and S10†). We conclude from the ^1^H NMR spectra that the proton signals of the phenoxy spacer remain unaffected by the nature of the SubPc substituents and in both cases appear shifted upfield into the range from 5.4 to 6.9 ppm as a consequence of the SubPc ring current.

**Fig. 1:**
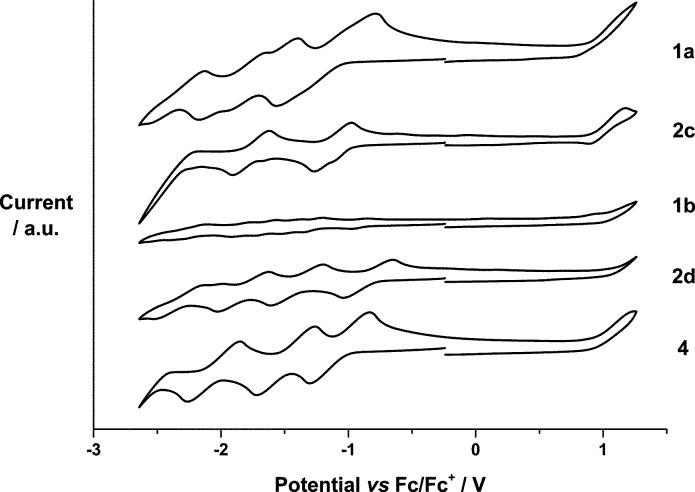
Cyclic voltammograms of **1a**, **2c**, **2d** and **4** (10^–3^ M) and **1b** (10^–4^ M) measured at 100 mV s^–1^ in *o*-dichlorobenzene with 0.05 M (*n*-Bu)_4_NPF_6_ as supporting electrolyte, glassy carbon as working electrode, Pt wire as counter electrode, and Ag wire as reference electrode.

The electrochemical properties of **1a**,**b** were examined and compared with the redox characteristics of their references, that is, **2c**,**d** and *N*-methylfulleropyrrolidine **4**, by CV (Fig. 1) and DPV (Fig. S39†) in *o*-DCB containing 0.05 M (*n*-Bu)_4_NPF_6_. The corresponding data are summarized in Table 1. Overall, **1a** and **1b** exhibit up to seven reductions and two oxidations in the cathodic and the anodic directions, respectively, which were assigned upon comparison with SubPcs **2c**,**d** and *N*-methylfulleropyrrolidine **4**.

**Table 1:** Electrochemical oxidation and reduction data in V *vs.* (Fc^+^/Fc) detected by DPV (sweep rate 0.02 V s^–1^) in *o*-DCB solutions (0.05 mol L^–1^ TBAPF_6_) at RT for **1a**,**b** and references **2c**, **2d**, and **4** (*N*-methyl-3,4-fulleropyrrolidine)

		*E*_p,red_^7^	*E*_p,red_^6^	*E*_p,red_^5^	*E*_p,red_^4^	*E*_p,red_^3^	*E*_p,red_^2^	*E*_p,red_^1^	*E*_p,ox_^1^	*E*_p,ox_^2^	HOMO–LUMO gap
**1a**	SubPc centered			–2.21	–1.79		–1.18			+1.08	2.05
	C_60_ centered					–1.57		–1.06	+0.99		
**1b**	SubPc centered	–2.17		–1.62		–1.26		–0.91		+1.12	1.95
	C_60_ centered		–1.81		–1.42		–1.10		+1.04		
**2c**						–2.30	–1.71	–1.09	+1.02		2.11
**2d**					–2.19	–1.73	–1.34	–0.81	+1.18		1.99
**4**						–1.95	–1.40	–1.01	+1.04		2.05

For example, (perfluoro) SubPc **2c** reveals three reversible reductions at –1.09, –1.71, and –2.30 and one reversible oxidation at 1.02 V. Similarly, four reversible reductions at –0.81, –1.34, –1.73, and –2.19 V and one irreversible oxidation at 1.18 V are noted for hexa(pentylsulfonyl) SubPc **2d**. With the latter in hand, the reductions of SubPc–C_60_ conjugate **1a** evolving at –1.18, –1.79, and –2.21 V are ascribed to SubPc centered processes, whereas those at –1.06 and –1.57 V relate to processes involving C_60_ (Table 1). Oxidations at +0.99 and +1.08 V are C_60_ and SubPc centered, respectively. In **1b**, the presence of two strong acceptors, namely hexa(pentylsulfonyl) SubPc and C_60_, leads to a coalescence and irreversibility of the reductions followed by two oxidations.^19^ As such, seven reductions at –0.91, –1.10, –1.26, –1.42, –1.62, –1.81, and –2.17 V plus two oxidations at +1.04 and +1.12 V evolve (Table 1). Those at +1.04, –1.10, –1.42, and –1.81 V are C_60_ centered, while those at +1.12, –0.91, –1.26, –1.62, and –2.17 V correlate with either the oxidation or the reduction of hexa(pentylsulfonyl) SubPc.

From Fig. 1 and Table 1, we conclude that hexa(pentylsulfonyl) SubPc **2d** is more easily reduced and more difficult to be oxidized than (perfluoro) SubPc **2c**. In other words, the alkylsulfonyl groups impose stronger electron withdrawing character than the fluorine atoms. A corroborating trend is found in **1a** and **1b**, **1b** is more easily reduced and more difficult to be oxidized than **1a**, where the differences in reduction and oxidation are 150 and 50 mV, respectively. On the other hand, the corresponding first reductions in **1a** and **1b** are considerably 30 and 100 mV shifted if compared to those of the reference SubPcs **2c** and **2d**, suggesting stronger ground-state interactions between the electron donor and the electron acceptor subunits in **1b**. The HOMO–LUMO gaps were calculated using the data (Table 1). The estimated values are found to be in good agreement with the SubPc ground state absorption features. Thus, **1b** containing hexa(pentylsulfonyl) SubPc reveals strong absorption at 582 nm, which is 7 nm red-shifted in comparison with the absorption of perfluorinated **1a** at 575 nm (Fig. S7 and S13†). The precursors (**2a**,**b**) and the reference SubPcs (**2c**,**d**) follow the same trend. As such, the absorption spectra of **1a** and **1b** are best described as the superimposition of the absorption characteristics of the single components, namely SubPcs **2c** and **d**, on one hand, and *N*-methylfulleropyrrolidine **4**, on the other hand.

**Fig. 2:**
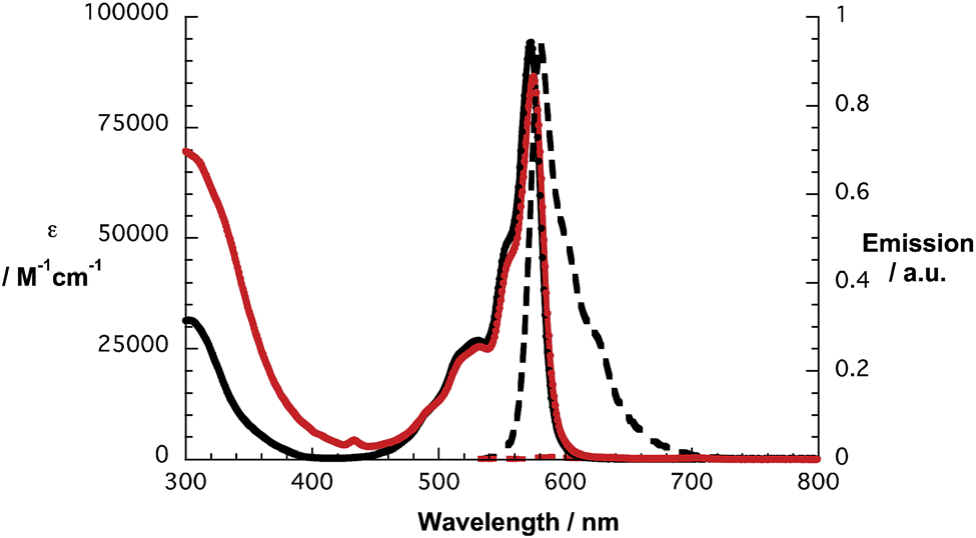
Absorption (solid lines) and fluorescence spectra (dashed lines) of SubPc **2c** (black lines) and conjugate **1a** (red lines) in toluene.

In line with what was observed in steady state absorptions, the nature of the peripheral substituents influences the singlet excited state properties of **1a** and **1b** (Fig. 2). In reference experiments, a solvent independent fluorescence quantum yield of 0.17 is determined for **2c**. In contrast, **2d** exhibits solvent dependent quantum yields of 0.15, 0.07 and 0.06 in toluene, THF and benzonitrile, respectively. In reference to the aforementioned, fluorescence assays with **1a** and **1b** give rise to strong quenching of the SubPc fluorescence with values from 0.0007 to 0.0012.

To assign the differential absorption changes – *vide infra* – spectroelectrochemical reductions of **2c** or **2d** to form the corresponding (SubPc(F_12_))˙^–^ or (SubPc(SO_2_C_5_H_11_)_6_)˙^–^ radical anions were deemed important. On one hand, reduction of **2c** results in two maxima at 455 and 655 nm, two shoulders at around 475 and 610 nm, and a minimum at 570 nm. On the other hand, upon reduction of **2d** spectral characteristics with maxima at 480, 545, 620, and 735 nm complemented by minima at 534 and 581 nm evolve (Fig. S44†). Notably, pulse radiolytical reductions with **2c** or **2d** in deaerated toluene–2-propanol–acetone mixtures (8 : 1 : 1 v/v) result in quantitatively similar spectra with characteristic fingerprints at 610 and 620 nm, respectively. For establishing the signature of (C_60_)˙^+^ we turned to time-resolved investigations. From, for example, pulse radiolysis experiments with *N*-methyl-3,4-fulleropyrrolidine **4** we derive characteristic maxima for (C_60_)˙^+^ in the near infrared at 860 and 950 nm. As a complement, we probed the scandium-ion promoted photoinduced oxidation of **4** by *p*-chloranil in deaerated benzonitrile. Here, a broad signature in the near infrared with maxima at 880, 960, and 1080 nm corroborate the pulse radiolytic experiments with **4**. Important is the determination of the extinction coefficient for the 880 nm maximum of approximately 3000 M^–1^ cm^–1^.

Insights into the excited state characteristics of **4**, **2c**, **2d**, **1a**, and **1b** came from transient absorption measurements following femtosecond and nanosecond excitation. For *N*-methylfulleropyrrolidine, differential absorption changes evolve immediately after the 387 nm laser excitation, which are characterized by maxima at 500 and 900 nm. The C_60_ singlet excited state (1.8 eV) decays with 1.4 ± 0.1 ns to afford the energetically lower lying triplet excited state (1.5 eV), whose characteristics are a transient maximum at 700 nm and a transient lifetime of up to 20 μs in the absence of molecular oxygen. **2c** reveals, upon excitation at 530 nm, differential absorption changes, which include transient maxima at 440 and 600 nm as well as transient minima at 514, 575, and 635 nm (Fig. S40†). In addition, a broad near infrared feature spans from 650 to 1200 nm, which peaks around 710 nm. These features relate to the SubPc singlet excited state (2.16 eV) and transform with 1.9 ± 0.1 ns into the corresponding triplet excited state (1.4 eV). Transient absorption spectra of the latter maximize at 470 and 610 nm and minimize at 532 and 570 nm. They all give rise to the same triplet excited state lifetime of 39 ± 2 μs. For **2d**, commencing with the conclusion of the 530 nm excitation, differential absorption changes develop in the form of transient maxima at 424, 474, 623, and 660 nm, a broad tail extending far into the near infrared, as well as transient minima at 533 and 583 nm (Fig. S41†). These SubPc singlet excited state (2.12 eV) related transient absorption features undergo intersystem crossing to the corresponding triplet excited state (1.4 eV), which exhibits a broad transient in the visible part of the spectrum that maximizes at 470 and 620 nm and minimizes at 533 and 583 nm. Owing to the presence of the sulfur, singlet excited state lifetimes of 1220 ± 20, 420 ± 10, and 415 ± 10 ps evolve in toluene, THF, and benzonitrile, respectively. In contrast, the triplet excited state lifetime is 53 ± 2 μs.

530 nm excitation of **1a** results in the exclusive formation of the SubPc singlet excited state (Fig. S42†). In particular, transient maxima at 450, 600, and 720 nm as well as transient minima at 515, 575, and 635 nm are formed instantaneously and decay in the presence of C_60_ rapidly with about 1.5 ± 0.3 ps in toluene, THF, and benzonitrile. As the SubPc singlet excited state decays a broad near-infrared transient, which maximizes at 910 nm, grows in, and, whose features are attributed to the C_60_ singlet excited state. Interestingly, we did not find the characteristic C_60_ triplet feature at the end of the C_60_ singlet excited state deactivation. On the contrary, maxima at 470 and 615 nm as well as a minimum at 575 nm are noted. Earlier we have established such features as reliable attributes of the SubPc triplet excited state. From this we infer that the C_60_ triplet excited state (1.5 eV), once formed, undergoes a thermodynamically allowed transfer of triplet excited state energy to SubPc (1.4 eV).

The kinetics at the 470 and 615 nm maxima, which allowed us to follow the generation of the SubPc triplet excited state, further furnish the following kinetic assignment: the rate-determining step in the SubPc triplet excited state formation is the C_60_ centered intersystem crossing. A global analysis discloses kinetics that are very similar (1.6 ± 0.1 ns) to the inherent intersystem crossing dynamics seen for C_60_.^20^ When turning to excitation of **1b** at 530 nm (Fig. S43†), its singlet excited state, that is, maxima at 427, 474, 623, and 660 nm, and minima at 535 and 585 nm, decay in the presence of C_60_ rapidly within 1.5 ± 0.3 ps to form accordingly the C_60_ singlet excited state with its 910 nm maximum. Like for **1a**, we did not find the characteristic C_60_ triplet feature. Instead, maxima at 470 and 625 nm as well as minima at 535 and 585 nm of the SubPc triplet excited state were concluded. Again, the triplet excited state energy of SubPc (1.4 eV) evolves from a thermodynamically allowed transfer of triplet excited state energy. The kinetics document that the rate-determining step is the C_60_ centered intersystem crossing (1.6 ± 0.1 ns).^21^

**Fig. 3:**
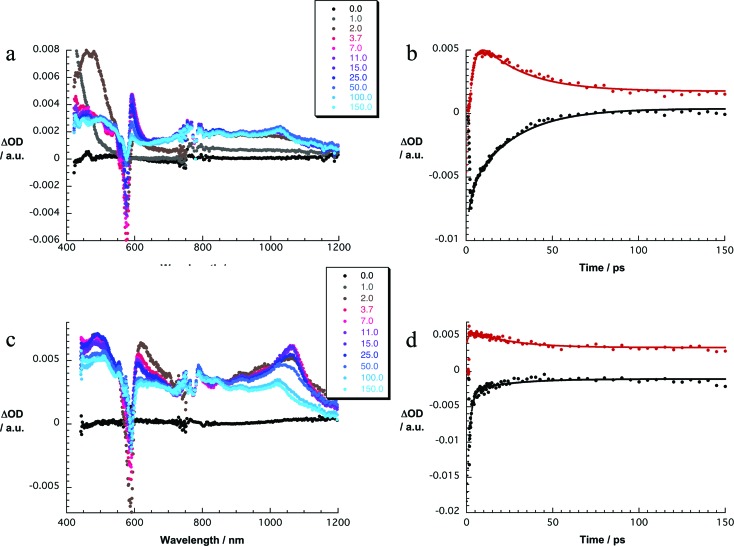
(a) Differential absorption spectra (visible and near-infrared) obtained upon femtosecond flash photolysis (320 nm) of **1a** (10^–5^ M) in argon-saturated toluene with several time delays between 0 and 150 ps at room temperature. (b) Time–absorption profiles of the spectra shown in (a) at 576 nm (black) and 591 nm (red) monitoring the charge separation and the charge recombination processes. (c) Differential absorption spectra (visible and near-infrared) obtained upon femtosecond flash photolysis (387 nm) of **1b** (10^–5^ M) in argon-saturated toluene with several time delays between 0 and 150 ps at room temperature. (d) Time–absorption profiles of the spectra shown in (c) at 589 nm (black) and 608 nm (red) monitoring the charge separation and the charge recombination processes.

**Fig. 4:**
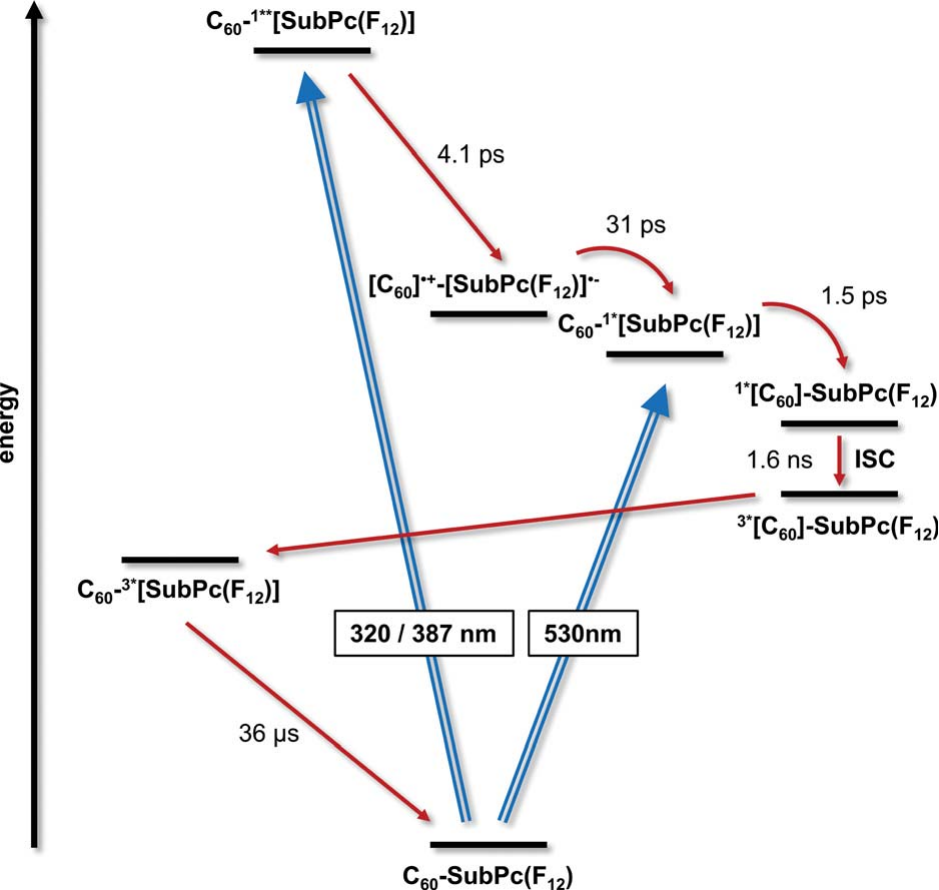
Energy level diagram of **1a** reflecting the different pathways of energy and electron transfer.

Exciting **1a** at 320 or 387 nm populates higher lying singlet excited states (S_2_: 3.4 ± 0.1 eV) of SubPc directly – the light partitioning at, for example, 320 nm is 1 : 1 (Fig. 3). The latter are discernable with maxima at 430, 600, and 995 and minima at 515 and 570 nm, which transform within 7 ± 1 ps to the lowest-energy singlet excited state in the absence of C_60_. In contrast to that, in **1a** the lifetime is shortened to 4.1 ± 0.2 ps (toluene), 3.5 ± 0.2 ps (THF), and 2.6 ± 0.5 ps (benzonitrile) by the presence of C_60_. The product of an instantaneous transformation includes maxima at 590, 840, 910, and 1020 nm and a minimum at 575 nm. These are in sound agreement with those noted upon pulse radiolytical generation of (C_60_)˙^+^ – *vide supra*. In the visible, a transient maximum at 595 nm and a transient minimum at 578 nm are clear fingerprints of (SubPc(F_12_))˙^–^. In other words, the close spectral resemblance between radiolytical and photophysical experiments prompts to the unprecedented formation of the (C_60_)˙^+^–(SubPc(F_12_))˙^–^ (2.17 eV) radical ion pair state, as a product of charge separation (Fig. 4). Both fingerprints served as reliable probes to determine the lifetime of the metastable (C_60_)˙^+^–SubPc(F_12_)˙^–^ radical ion pair state. Charge recombination proceeds with time constants of 31 ± 5 ps (toluene), 35 ± 5 ps (THF), and 38 ± 10 ps (benzonitrile) to populate the SubPc triplet excited state as the only discernable component. The singlet excited state of C_60_ (1.8 eV), which is partly excited at excitation wavelengths like 320 and 387 nm, is, however, insufficient in terms of thermodynamics to drive a charge transfer. Instead, the C_60_ singlet excited state related transient absorptions with maxima at 500 and 900 nm transform with a time constant of 1.6 ± 0.1 ns into the SubPc triplet excited state *via* triplet excited state energy transfer – *vide supra*.

Experiments with 387 nm excitation of **1b**, where the light partitioning is 2.6 : 1, lead to the formation of the singlet excited states of C_60_ and higher lying singlet excited states of SubPc (Fig. 3). The C_60_ singlet excited state (1.8 eV) related transient absorption features with maxima at 500 and 900 nm transform with a time constant of 1.6 ± 0.1 ns into the SubPc triplet excited state *via* triplet excited state energy transfer, similar to the 530 nm excitation experiments. However, the higher lying singlet excited SubPc states (S_2_: 3.3 ± 0.2 eV) are able to drive an ultrafast electron transfer evolving from C_60_ to SubPc. As a matter of fact, maxima at 481, 623, 655, and 1065 nm and minima at 532 and 585 nm decay fast in toluene, THF, and benzonitrile with lifetimes of around 0.6 ± 0.2, 1.4 ± 0.5, and 1.7 ± 0.5 ps, respectively. Simultaneously with the latter decay, new transitions develop in the visible and the near-infrared regions. Importantly, the new transients do not match the signature of the SubPc triplet excited state. Instead, maxima at 480, 550, 610, 840, 910, and 1025 nm as well as a minimum at 590 nm are discernable. Upon spectral comparison – *vide supra* – we ascribe the transitions in the visible to (SubPc(SO_2_C_5_H_11_)_6_)˙^–^, while those in the near-infrared correspond to (C_60_)˙^+^. In light of the aforementioned, we conclude an electron transfer from C_60_ to, for example, the SubPc singlet excited state to yield (C_60_)˙^+^–(SubPc(SO_2_C_5_H_11_)_6_)˙^–^ (1.95 eV). The (C_60_)˙^+^–(SubPc(SO_2_C_5_H_11_)_6_)˙^–^ radical ion pair state is short lived and decays with lifetimes of 16 ± 5, 21 ± 5, and 26 ± 5 ps in toluene, THF, and benzonitrile, respectively. At the end of this decay, the SubPc triplet excited state with maxima at 470 and 625 nm as well as minima at 535 and 585 nm emerges as the product of charge recombination.^22^

## Conclusions

To sum up, we have documented the comprehensive investigation regarding the covalent attachment of two different light harvesting/electron accepting SubPcs to C_60_ by means of the Prato reaction complemented by several steady-state and time-resolved assays. Most importantly, our photophysical assays corroborate unambiguously the ultrafast charge separation (in the range from 0.6 to 4.1 ps) between SubPcs and C_60_ upon photoexcitation at either 320 or 387 nm to form the one-electron oxidized form of C_60_ and the one-electron reduced forms of the two different SubPcs. This is, to the best of our knowledge, the first case of an *intramolecular* oxidation of C_60_ as part of an electron donor–acceptor conjugate for advanced solar energy conversion schemes by means of only photoexcitation. The charge separated state lifetime is at this state rather short lived with values of up to 38 ps. Currently, we are directing our efforts to extend the radical ion pair state lifetime by, for example, changing the energetics/thermodynamics of charge recombination.

## Supplementary Material

Supplementary information

Crystal structure data

## References

[cit1] (b) Wengenmair R. and Bührke T., Renewable energy: sustainable energy concepts for the future, Wiley-VCH, Weinheim, 2013, pp. 1–170.

[cit2] (a) Alberts B., Johnson A., Lewis J., Raff M., Roberts K. K. and Walter P., Molecular Biology of the Cell, Garland Science, New York, 5th edn, 2007, pp. 813–878.

[cit3] Kirner S., Sekita M., Guldi D. M. (2014). Adv. Mater..

[cit4] (b) Hirsch A., The Chemistry of the Fullerenes, John Wiley & Sons, 2008, pp. 1–215.

[cit5] Wang T., Wang C. (2014). Acc. Chem. Res..

[cit6] (a) Hasobe T., Handbook of Carbon Nano Materials, ed. F. D'Souza and K. M. Kadish, 2012, vol. 4, pp. 95–130.

[cit7] (a) Bottari G., Urbani M. and Torres T., Organic Nanomaterials: Synthesis, Characterization, and Device Applications, ed. T. Torres and G. Bottari, John Wiley & Sons, Inc., Hoboken, New Jersey, 2013, pp. 163–187.

[cit8] Kc C. B., Lim G. N., Zandler M. E., D'Souza F. (2013). Org. Lett..

[cit9] (b) Hudhomme P. and Williams R. M., Handbook of Carbon Nano Materials, ed. F. D'Souza and K. M. Kadish, 2011, vol. 2, pp. 545–591.

[cit10] (a) Cataldo F., Iglesias-Groth S. and Manchado A., in Fullerenes, Nanotubes and Carbon Nanostructures, 2012, vol. 20, pp. 656–671.

[cit11] (c) Biczok L. Linschitz H., J. Phys. Chem. A, 2001, 105 , 11051 –11056 , , and references therein .

[cit12] Fukuzumi S., Mori H., Imahori H., Suenobu T., Araki Y., Ito O., Kadish K. M. (2001). J. Am. Chem. Soc..

[cit13] Ohkubo K., Ortiz J., Martin-Gomis L., Fernandez-Lazaro F., Sastre-Santos A., Fukuzumi S. (2007). Chem. Commun..

[cit14] Rudolf M., Feng L., Slanina Z., Akasaka T., Nagase S., Guldi D. M. (2013). J. Am. Chem. Soc..

[cit15] Claessens C. G., Gonzalez-Rodriguez D., Rodriguez-Morgade M. S., Medina A., Torres T. (2014). Chem. Rev..

[cit16] Guilleme J., Gonzalez-Rodriguez D., Torres T. (2011). Angew. Chem., Int. Ed..

[cit17] Maggini M., Prato M. (1998). Acc. Chem. Res..

[cit18] Okusa K., Tanaka H., Ohira M. (2000). J. Chromatogr. A.

[cit19] Irreversibility is likely based on electron density rearrangement chemical reactions

[cit20] In this context, it is reassuring that the transients seen at the end of the femtosecond experiments match those at the beginning of the nanosecond experiment. Moreover, maxima at 470 and 610 nm, minima at 532 and 570 nm, and an excited state lifetime of 36 μs in the absence of oxygen perfectly agree with what was found for the SubPc triplet excited state of **2c**

[cit21] In the absence of oxygen, the SubPc triplet excited state lifetime is 20 ± 5 μs and, as such, agrees with what was found for **2d**

[cit22] Singlet oxygen quantum yields of **1b** were found to be as high as 0.49 (toluene), 0.24 (THF), and 0.65 (benzonitrile) and support the assignment that the triplet excited state evolves as the product of charge recombination. Please note that the singlet oxygen yields in **2d** are 0.39 (toluene), 0.09 (THF), and 0.26 (benzonitrile)

