# Supplementary material for: Taming C60 fullerene: tuning intramolecular photoinduced electron transfer process with subphthalocyanines
Source: Chem Sci. 2015 Apr 16;6(7):4141–7. doi: 10.1039/c5sc00223k (PMC5707453; doi:10.1039/c5sc00223k)
Supplement: Supplementary file 1 [file SC-006-C5SC00223K-s001.pdf]

Electronic Supplementary Material (ESI) for Chemical Science.
This journal is © The Royal Society of Chemistry 2015

Supporting Information

Taming C_{60} fullerene: tuning intramolecularphotoinduced electron transfer process with subphthalocyanines

Marc Rudolf,[a] Olga Trukhina,[b,c] Josefina Perles,[d] Lai Feng,[e,f]Takeshi Akasaka,*[e,g,h,i] Tomas Torres*[b,c] and Dirk M. Guldi*[a]

[a] *Department of Chemistry and Pharmacy and Interdisciplinary Center for Molecular Materials (ICMM), Friedrich-Alexander-Universität Erlangen-Nürnberg, 91058 Erlangen (Germany)*

[b] *Department of Organic Chemistry, Autonoma University of Madrid, Cantoblanco, 28049 Madrid (Spain)*

[c] *IMDEA Nanoscience, Faraday 9, 28049 Madrid (Spain)*

[d] *Interdepartamental Research Service (SIDI), Lab. of High Resolution X-Ray Diffraction of Monocrystals, Autonoma University of Madrid, Cantoblanco, 28049 Madrid (Spain)*

[e] *Life Science Center of Tsukuba Advanced Research Alliance, University of Tsukuba, 305-8577 Tsukuba (Japan)*

[f] *College of Physics, Optoelectronics and Energy & Collaborative Innovation Center of Suzhou Nano Science and Technology, Soochow University, 215006 Suzhou (China)*

[g] *Foundation for Advancement of International Science, 305-0821 Tsukuba (Japan)*

[h] *Department of Chemistry, Tokyo Gakugei University, 184-8501 Koganei (Japan), College of Materials Science and Engineering, Huazhong University of Science and Technology, 430074 Wuhan (China)*

[i] *College of Materials Science and Engineering, Huazhong University of Science and Technology, 430074 Wuhan (China)*

*E-mail:dirk.guldi@fau.de, akasaka@tara.tsukuba.ac.jp, tomas.torres@uam.es

Contents

		Page
1.	General	2
2.	Synthesis	4
3.	Electrochemistry	34
4.	Time resolved absorption	35
5.	Crystallographic data	40
6.	References	43

1. General

Chemicals were purchased from Aldrich Chemical Co and from Alfa Aesar Co, Inc. and used as received without further purification. Solid hygroscopic reagents were dried under vacuum before use. Glassware was oven-dried at 110 ^{o}C overnight every time before use. Reaction solvents werethoroughly dried before use according standard procedures. All manipulations were carried out using Schlenk techniques under an atmosphere of dry argon. Column chromatography was carried out using silica gel Merck-60 (230-400 mesh, 60 Å) as the solid support. TLC analyses were performed on aluminum sheets precoated with silica gel 60 F254 (E. Merck). Size-exclusion chromatography (GPC) was carried out using Bio-Beads S-X1 support (BIO-RAD) as a stationary phase and toluene as a mobile phase. High-performance liquid chromatography (HPLC) wasperformed on an Agilent 1100 LC (Agilent Technologies), using ø4.6×250 mm Buckyprep column (Cosmosil). A mixture of toluene-dichloromethane-acetonitrile = 89:10:1, vol %, at 0.5 mL/min flow rate was employed. Matrix-assisted laser desorption ionization (coupled to a time-of-flight analyzer) experiments (MALDI-TOF) were carried out using BrukerUltraflex III mass spectrometer, in both positive and negative ion modes. A convenient matrix for these measurements is indicated for each compound. NMR spectra were measured on a Bruker AV-300, Bruker AV-400 or a Bruker DRX-500 spectrometer, locked on deuterated solvents. Carbon chemical shifts are measured in ppm relative to trimethylsilane (TMS), downfield from TMS using the resonance of the deuterated solvent as the internal standard. The assignment of the NMR signals was supported in some cases by COSY spectra (400 MHz). UV/Vis spectra were recorded on a Jasco V-660-spectrophotometer in spectroscopic grade toluene. IR spectra were collectedonBruker Vector 22 spectrophotometer.

Compounds **2c**,[1]**3a**,[2]**4**,[3]**7**[4]were prepared according to the synthetic procedures reported elsewhere; **1a**[5]and **2a**[2,6]have been described previously and their synthesis was further improved in the present work.

Steady-State Emission: The spectra were recorded on a FluoroMax 3 fluorometer (Vis detection) and on a Fluorolog spectrometer (NIR detection). Both spectrometers were built by HORIBA JobinYvon. The measurements were carried out at room temperature.

Time-Resolved Absorption: femtosecond transient absorption studies were performed with 320, 387, 530, and 568 nm laser pulses (1 kHz, 150 fs pulse width) from amplified Ti:Sapphire laser systems (CPA-2101 and CPA-2110 from Clark-MXR, Inc.), the laser energy was 200 nJ. Nanosecond laser flash photolysis experiments were performed with 355 and 532 nm laser pulses from a Quanta-Ray CDR Nd:YAG system (6 ns pulse width) in a front face excitation geometry.

Photoinduced Oxidation of C_{60}-Py: Adeaeratedbenzonitrile solution containing N-methylfulleropyrrolidine (1.5×10^{-4} M) and *p*-chloranil (0.04 M) in the presence of $Sc(OTf)_3$ (0.11 M) was excited with the help of the Quanta-Ray CDR Nd:YAG system at 532 nm (10 mJ per pulse).

Electrochemistry: Cyclic voltammograms (CVs) and differential pulse voltammograms (DPVs) were recorded on Metrohm μAutolabType III FRA2electrochemical analyzer.Measurements were carried out in a home-built one-compartment cell using a three-electrode configuration, *o*-dichlorobenzene (*o*-DCB) as a solvent and 0.05 M tetrabutylammoniumhexafluorophosphate ($TBAPF_6$) as a supporting electrolyte. A carbon-glassy electrode was used as the working electrode and a platinum wire as a counter electrode. All potentials were recorded against a silver wirepseudo-reference electrode and corrected against Fc^+/Fc redox couple. DPV and CV were measured at scan rates of 20 and 100 mVs^{-1}, respectively. Prior to each voltammetric measurement, the cell was degassed by bubbling with argon for about 20 min. Electrochemical measurements were performed using a concentration of approximately 1×10^{-3} M for the compound in question.

2. Synthesis

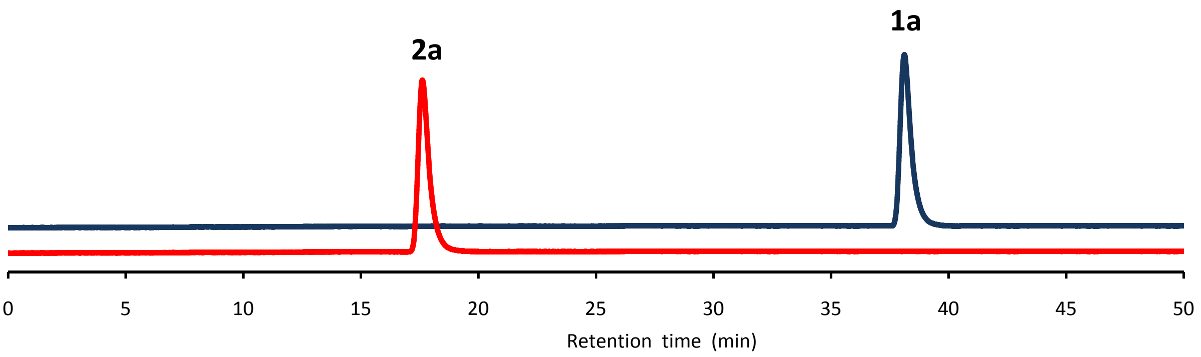

Figure S1:HPLC profiles of the isolated **1a** and **2a** on Buckyprep column (ø4.6×250 mm, Cosmosil, toluene-dichloromethane-acetonitrile = 89:10:1, vol %, 0.5 mL/min flow rate, at wavelength of 572 nm).

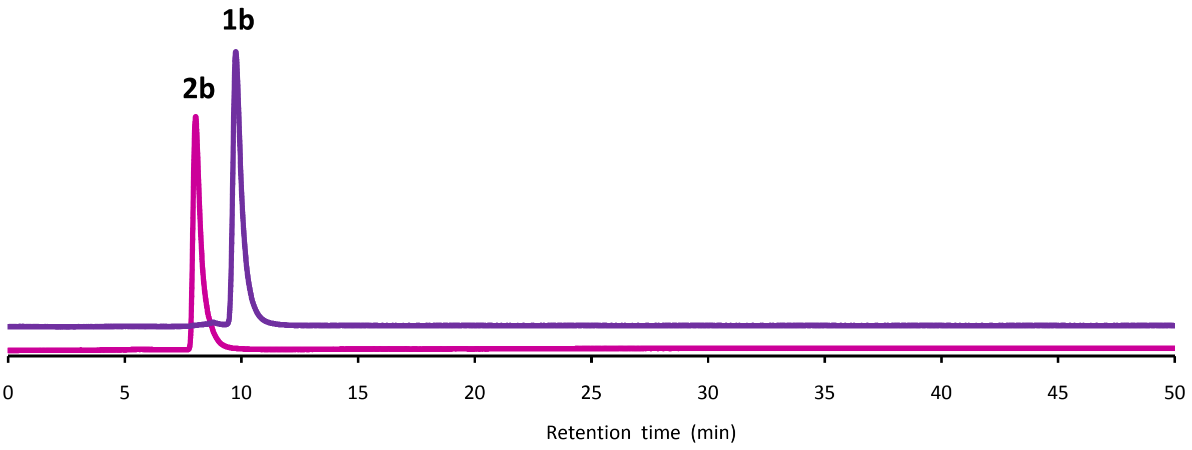

Figure S2:HPLC profiles of the isolated **1b** and **2b** on Buckyprep column (ø4.6×250 mm, Cosmosil, toluene-dichloromethane-acetonitrile = 89:10:1, vol %, 0.5 mL/min flow rate, at wavelength of 579 nm).

- 3´-(N-Methyl-3´´,4´´-[60]fulleropyrrolidin-2´´-yl)phenoxy-[1,2,3,4,8,9,10,11,15,16,17,18-dodecafluorosubphthalocyaninato]boron(III) **1a**

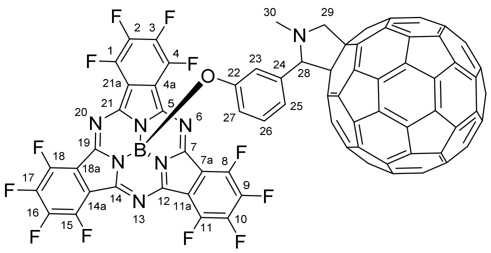

A mixture of (formylphenoxy) F_{12}SubPc **2a** (22 mg, 0.03 mmol), C_{60} fullerene (43 mg, 0.06 mmol), N-methylglycine (8 mg, 0.09 mmol) in dry toluene (30 mL) was sonicated for approx. 10 min, until all the C_{60} got dissolved. The reaction mixture was stirred at reflux under argon atmosphere for 8 hours untilno **2a** was detected by TLC. The solution was then cooled to room temperature and poured onto a silica gel column, followed by the elution of products with toluene in the following sequence: unreacted C_{60}, monoadduct**1a**, bisadducts. Final purification of **1a**from bisadducts was achieved by using a Bio-Beads column and toluene as an eluting solvent. Rotary evaporation of toluene resulted in a purple residue, that was suspended in hexane, filtered, dried in vacuum, yielding **1a** as a purple solid: 28 mg (63%).R_f = 0.8 (toluene).

^{1}H NMR (300 MHz, $CDCl_3$):δ(ppm) = 7.13-6.92 (m, 1H; H-25), 6.87-6.81 (dd, $J_o = J_{o'}$ = 7.6 Hz, 1H; H-26), 5.95-5.85 (m, 1H; H-23), 5.37-5.34 (d (br), J_o = 7.6 Hz, 1H; H-27), 4.91 (d (br), ^2J = 9.4 Hz, 1H; H-29), 4.54 (s (br), 1H; H-28), 4.13 (d, ^2J = 9.5 Hz, 1H; H-29’), 2.67 (s, 3H; H-30);^{13}C NMR (100.6 MHz, $CDCl_3$): δ (ppm) = 155.9, 153.7, 152.9, 152.8, 148.5 (6C; C-5, C-7, C-12, C-14, C-19, C-21), 146.1, 145.9, 145.7, 145.5, 145.2, 145.1, 145.0, 144.3, 144.1, 143.7, 143.0, 142.8, 142.5, 141.8, 141.4, 141.3, 139.9, 139.1, 138.6, 135.8, 135.6, 135.4 (C_{60}^{sp2}, C-F), 129.6 (1C; C-26), 128.3 (1C; C-25), 123.3 (1C; C-27), 122.3 (1C; C-24), 118.4 (1C; C-23), 115.1 (6C; C-4a, C-7a, C-11a, C-14a, C-18a, C-21a), 82.5 (1C; C-28), 69.8 (1C; C-29), 68.7 (2C; C_{60}^{sp3}), 39.8 (1C; C-30);MALDI-TOF MS (negative mode, DCTB): found: 1497.1 *m/z,* calc. for $C_{93}H_{10}B_1F_{12}N_7O_1$, $[M]^-$: 1497.1; IR (KBr): ν (cm^{-1}) =2924 (C-H), 2785, 1651 (C=N), 1605 (C=C), 1535, 1475 (C-F), 1381, 1265 (C-N), 1219, 1173, 1113 (C-C), 1063 (B-O), 970, 716, 600 (C-H), 530;UV-vis (toluene): λ_{max}(nm) (log ε) =575 (4.9), 559 (sh), 534 (4.4), 520 (sh), 498 (sh), 435, 312 (4.8).

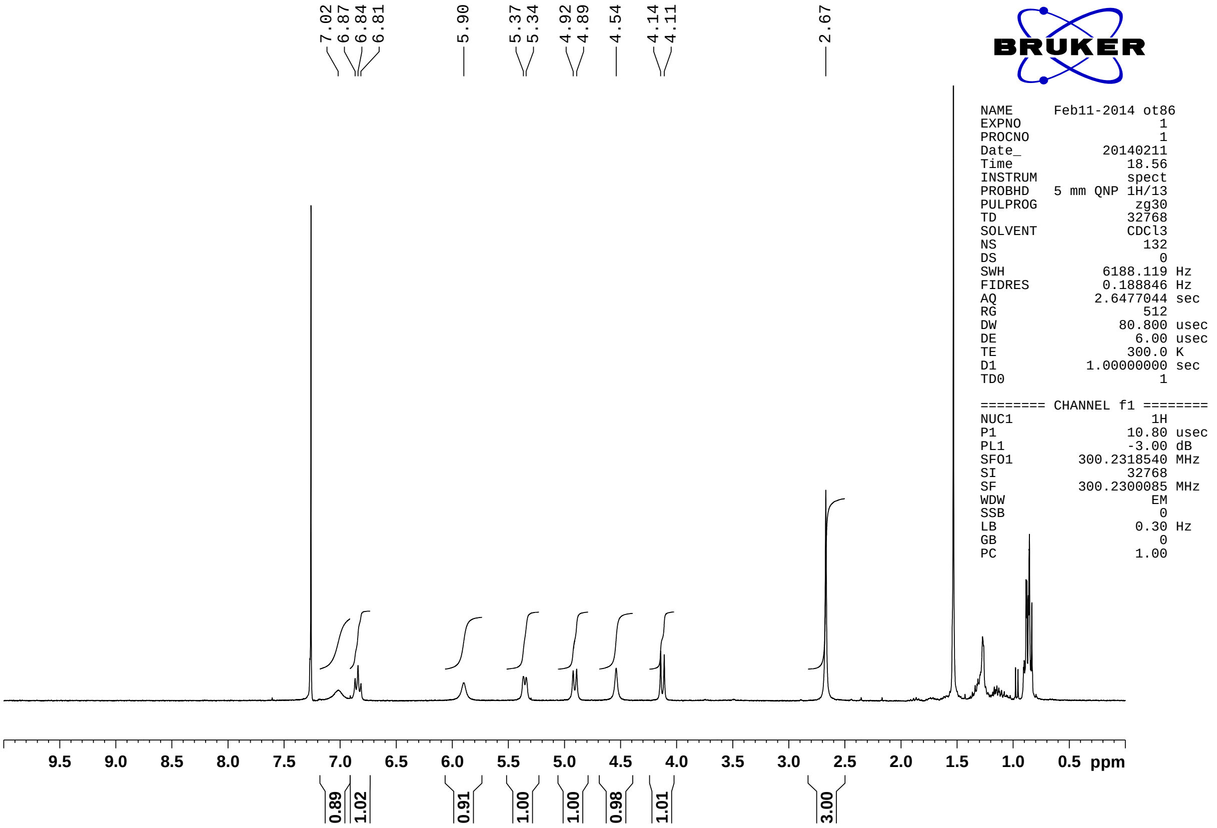

Figure S3: ^{1}H NMR spectrum (300 MHz, $CDCl_3$) of **1a**.

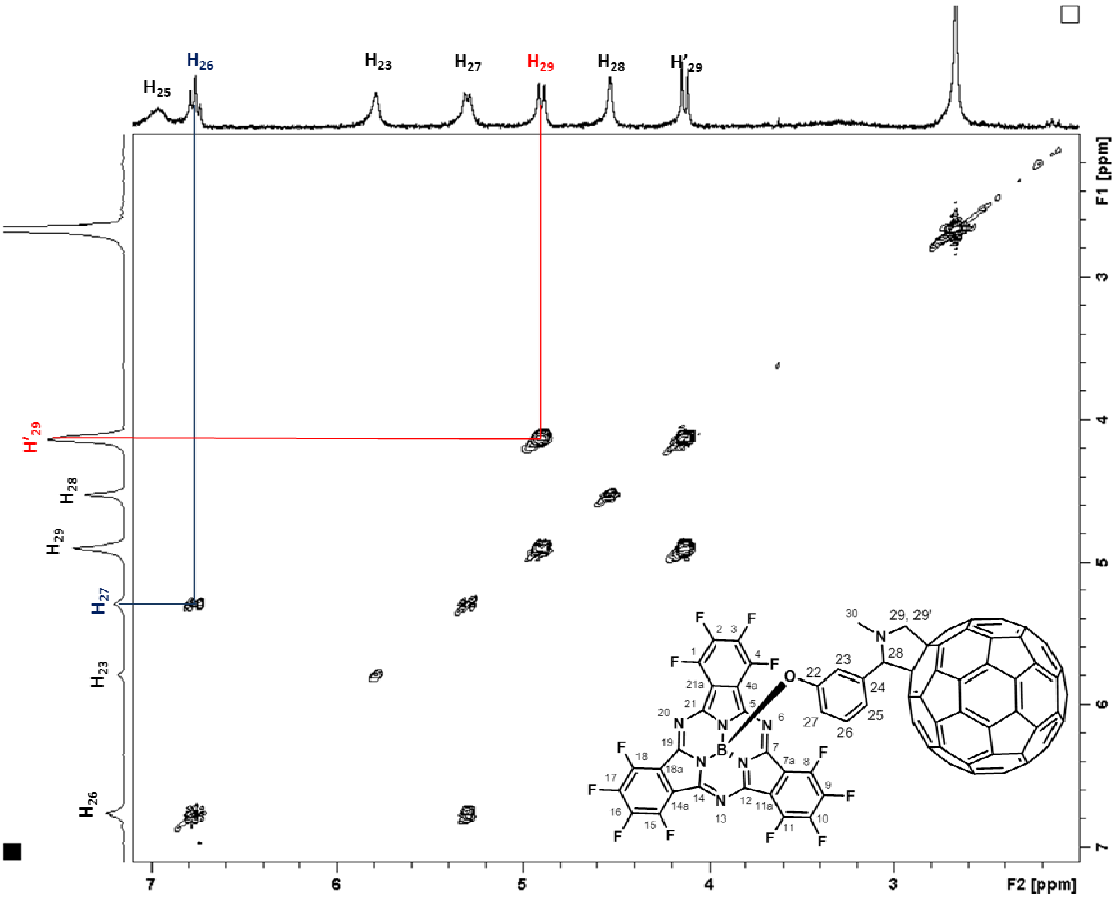

Figure S4: ^{1}H-^{1}H COSY NMR spectrum (300 MHz, $CDCl_3$) of **1a**.

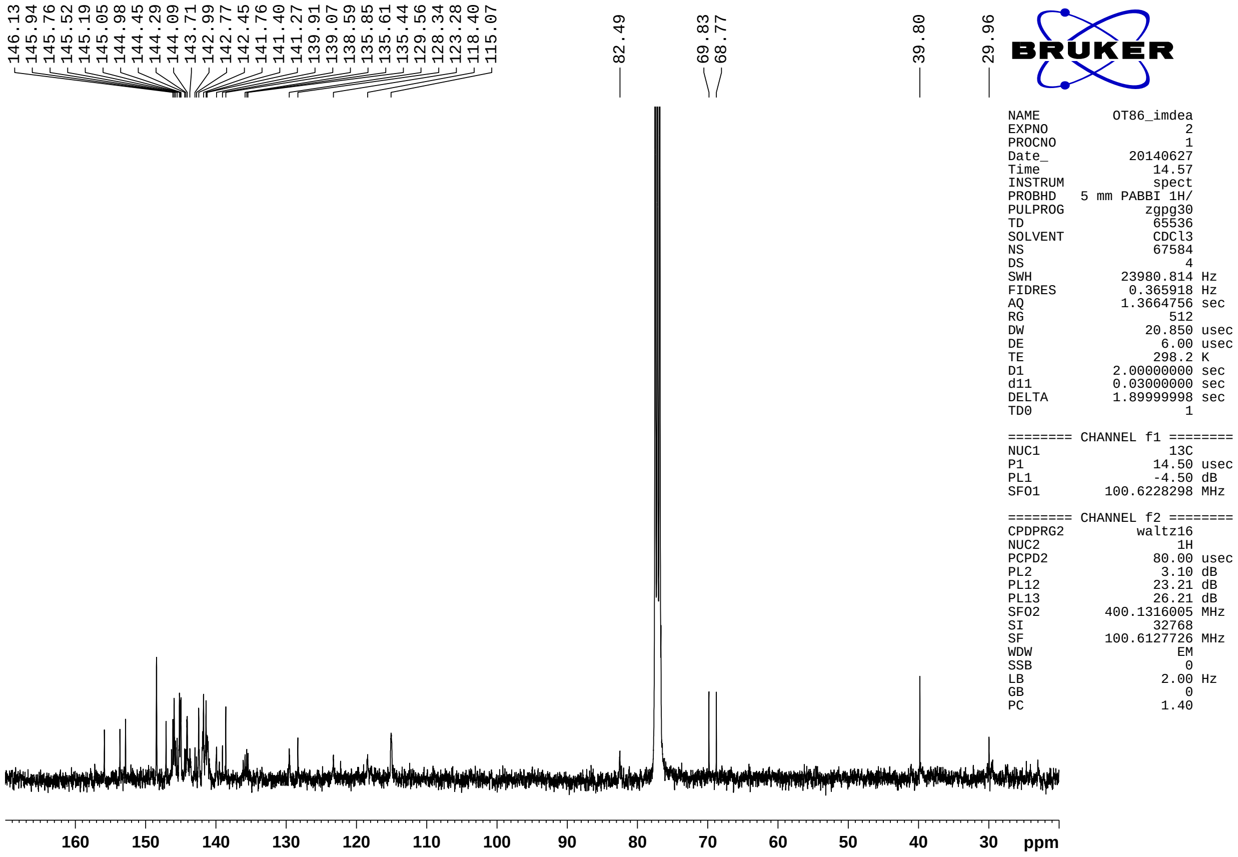

Figure S5: ^{13}C NMR spectrum (100.6 MHz, $CDCl_3$) of **1a**.

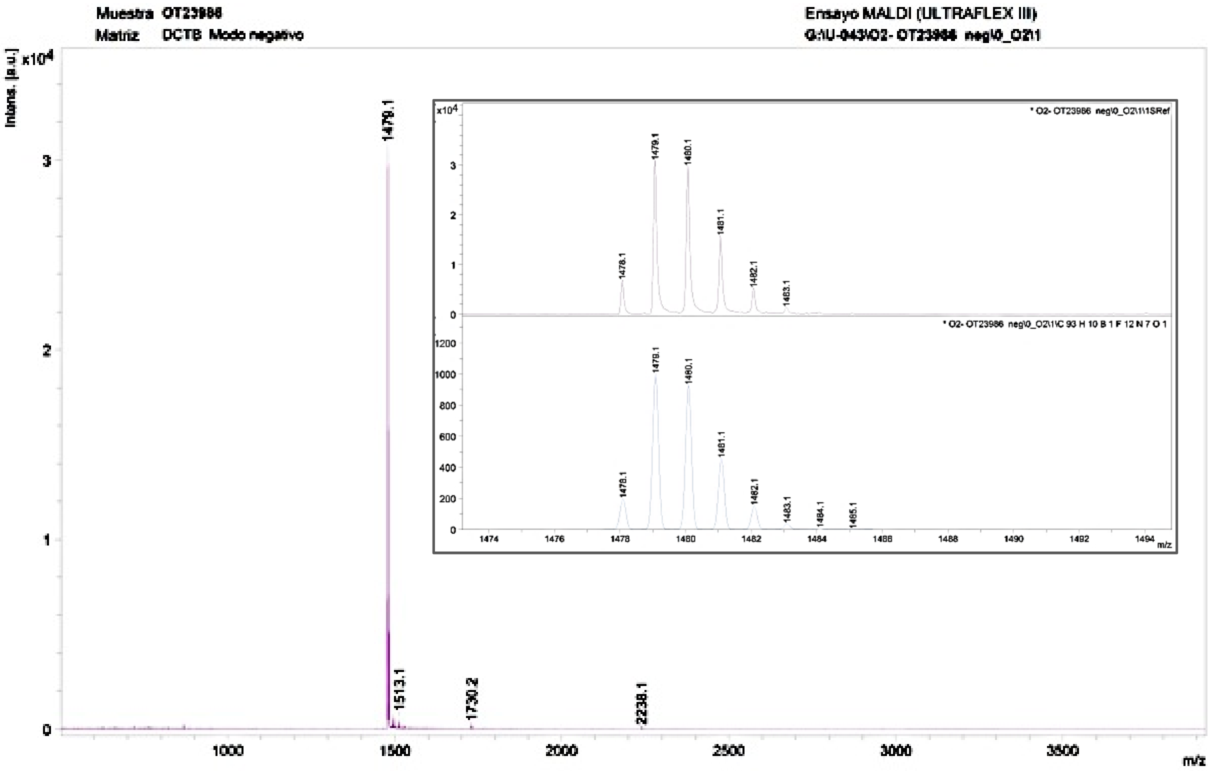

Figure S6: MALDI-TOF mass spectrum of **1a**, (DCTB,negative mode), (inserted graph) isotopic distribution: observed (top) *vs* calculated (bottom).

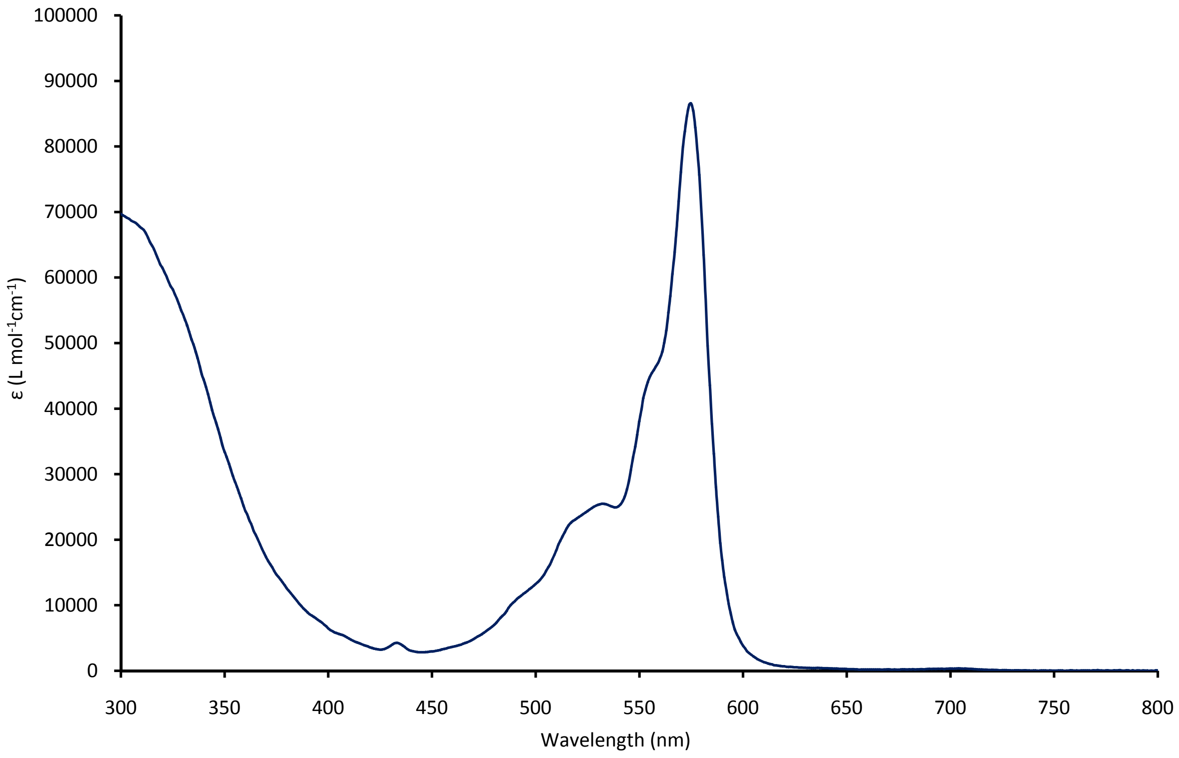

Figure S7: UV-vis spectrum of **1a** in toluene.

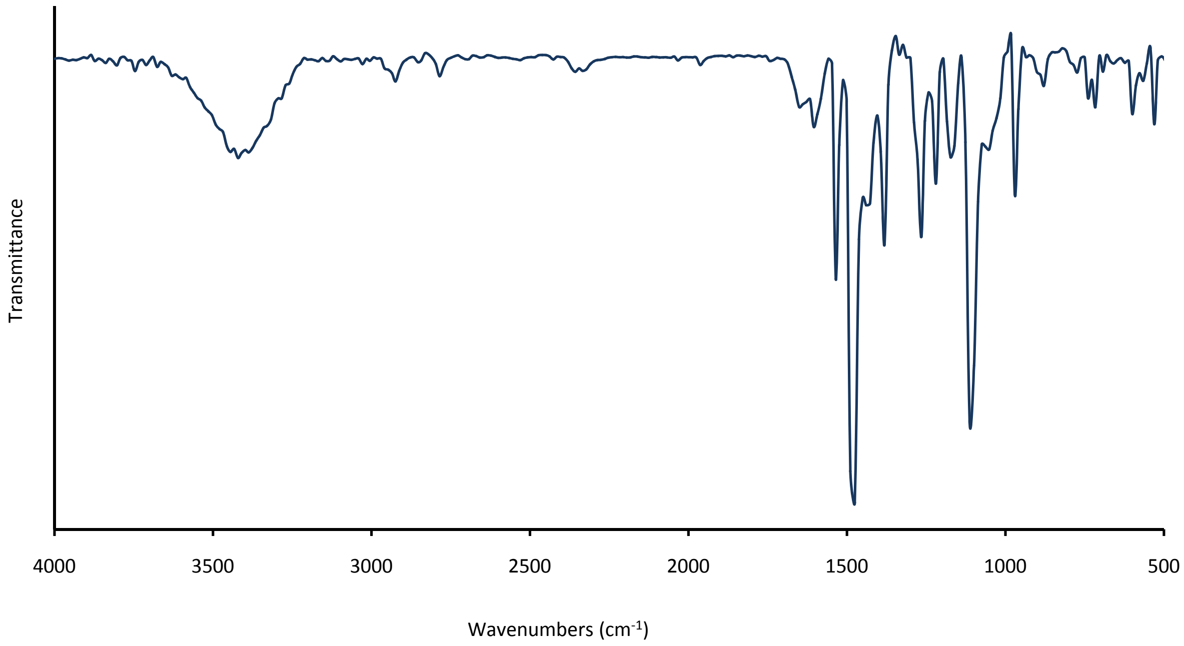

Figure S8: IR spectrum of **1a** (KBr).

- 3´-(N-Methyl-3´´,4´´-[60]fulleropyrrolidin-2´´-yl)phenoxy-[2,3,9,10,16,17-hexa(pentylsulfonyl)-subphthalocyaninato]boron(III) **1b**

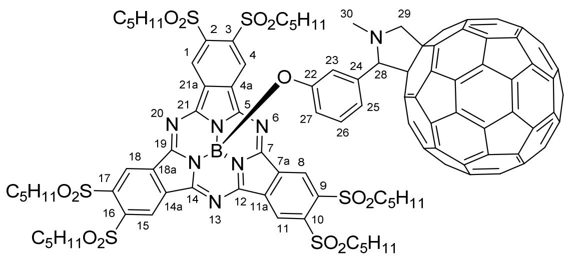

A mixture of (formylphenoxy) $(SO_2C_5H_{11})_6$SubPc**2b** (37 mg, 0.03 mmol), C_{60} fullerene (43 mg, 0.06 mmol), N-methylglycine (8 mg, 0.09 mmol) in dry toluene (40 mL) was sonicated for approx. 10 min, until all the C_{60} got dissolved. The reaction mixture was stirred at reflux under argon atmosphere for 2 hours untilno **2b** was detected by TLC. The solution was then cooled to room temperature and poured onto a silica gel column, followed by the elution of products with a mixture of toluene-dicloromethane (1:1vol), containing 0,8 % vol of ethanol, in the following sequence: unreacted C_{60}, monoadduct**1b**, bisadducts. Due to high instability of sulfonyl-substituted SubPc on silica gel, an additional chromatography on cyanopropyldichlorosilyl-modified silica gel as a solid support [], elutingwith a mixture of toluene-dicloromethane (1:1 vol), was neededto eliminate SubPc decomposition products. Final purification from bisadducts was achieved by using a Bio-Beads column and toluene-dicloromethane (1:1) as an eluting mixture. Rotary evaporation of solvents resulted in a violetresidue, that was suspended in hexane, filtered, dried in vacuum, yielding **1b** as a violet solid: 14 mg (24%).R_f = 0.5 (toluene-ethylacetate = 3:1, vol).

^{1}H NMR (500 MHz, $CDCl_3$): δ(ppm) = 9.75-9,74 (ss, 6H; H-1, H-4, H-8, H-11, H-15, H-18), 7.1-6.9 (m, 1H; H-25), 6.88-6.70 (m, 1H; H-26), 5.90-5.60 (m, 1H; H-23), 5.50-5.20 (m, 1H; H-27), 4.88 (d (br), ^2J= 7.90 Hz, 1H; H-29), 4.52 (s (br), 1H; H-28), 4.12 (d (br), ^2J= 9.1 Hz, 1H; H-29’), 3.84-3.58 (m, 12H; SO_2**CH_2**), 2.61 (s, 3H; H-30), 1.99-1.82 (m, 12H; SO_2CH_2**CH_2**),1.46-1.26 (m, 24H; $SO_2(CH_2)_2$(**CH_2**)$_2$), 0.88-0.85 (m, 18H; $SO_2(CH_2)_4$**CH_3**); ^{13}C NMR (75.5 MHz, $CDCl_3$): δ(ppm) = 156.3, 153.9, 153.3, 153.1, 152.2 (6C; C-5, C-7, C-12, C-14, C-19, C-21), 152.1 (1C; C-22), 146.2 (6C; C-2, C-3, C-9, C-10, C-16, C-17), 141.9, 141.1, 139.6, 139.1, 138.7, 137.9, 132.8 (6C; C-4a, C-7a, C-11a, C-14a, C-18a, C-21a), 129.0 (1C; C-26), 128.9 (1C; C25), 128.3, 125.4, 124.7 (1C; C-27), 118.7 (1C; C-23), 82.5 (1C; C-28), 69.7 (1C; C-29), 68.9 (2C; C_{60}^{sp3}), 57.39 (6C; SO_2CH_2), 39.9 (1C; C-30), 30.6, 22.2, 22.0 (18C; SO_2CH_2(**CH_2**)$_3$), 13.9 (6C; CH3); MALDI-TOF MS (negative mode, DCTB): found: 2067.4 *m/z,* calc. for $C_{123}H_{82}B_1N_7O_{13}S_6$, $[M]^-$: 2067.4; HR MALDI-TOF MS (DCTB, PMMANa2100, NaI) found: 2067.4380 *m/z,* calc. for $C_{123}H_{82}B_1N_7O_{13}S_6$, $[M]^+$: 2067.4400; found: 2090.4274 *m/z,* calc. for $C_{123}H_{82}B_1N_7Na_1O_{13}S_6$, $[M+Na]^+$: 2090.4298; IR (KBr): ν(cm^{-1}) = 2959, 2866 (C-H), 1605 (C=C), 1466, 1383, 1312, 1285, 1177 (S=O), 1149, 1097 (B-O), 710, 652; UV-vis (toluene): λ_{max} (log ε) = 582 (5.0), 566 (sh), 544 (sh), 534 (sh) 436, 314 (4.9).

57.49 (6C; SO_2CH_2), 30.66, 22.60, 22.39 (18C; SO_2CH_2(**CH_2**)$_3$), 14.04 (6C; CH3)

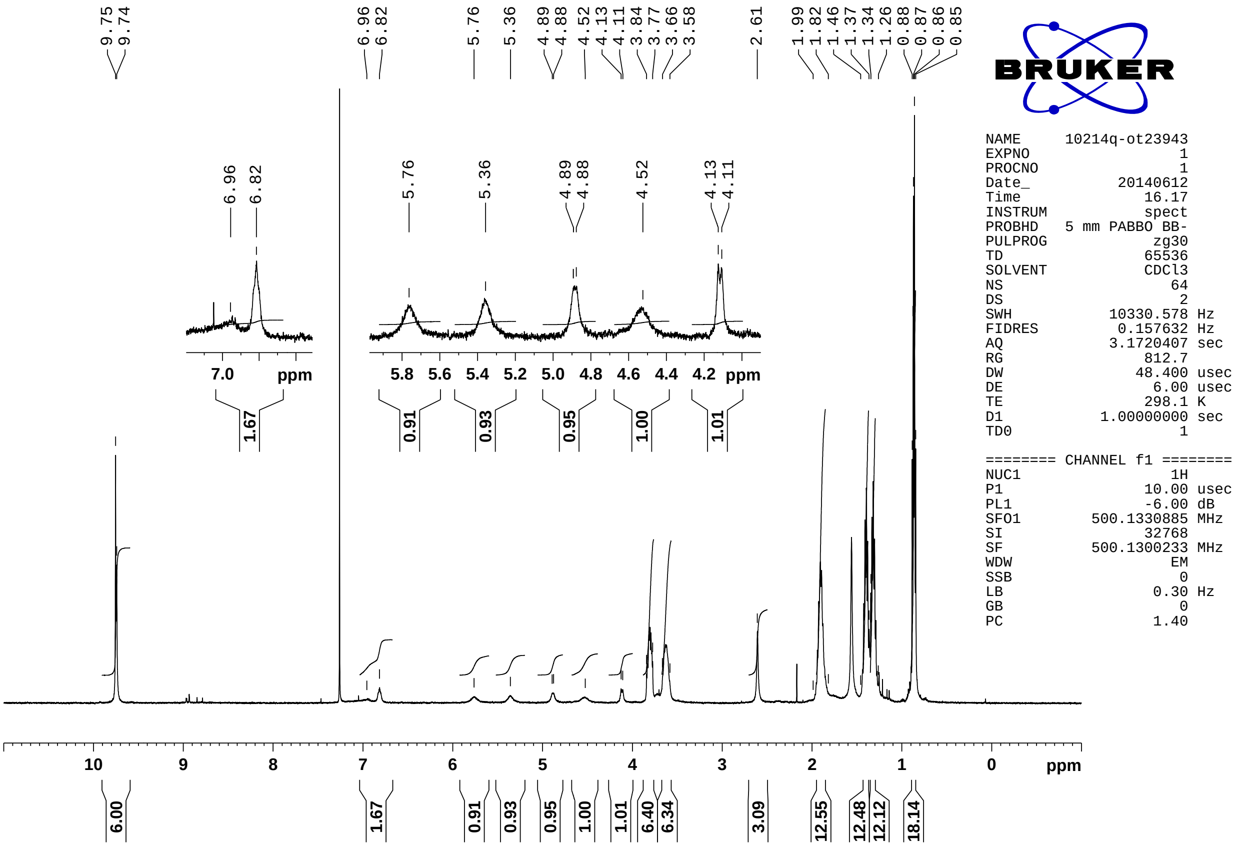

Figure S9: ^{1}H NMR spectrum (500 MHz, $CDCl_3$) of **1b**.

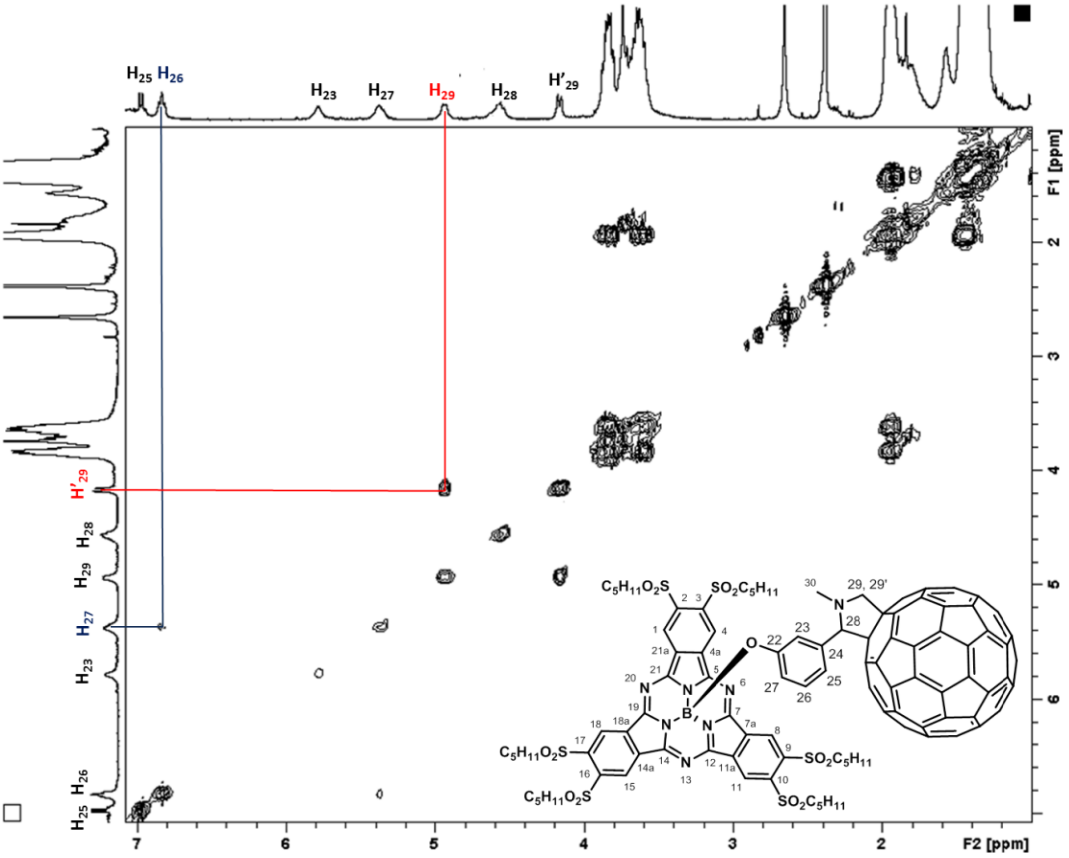

Figure S10: ^{1}H-^{1}H COSY NMR spectrum (300 MHz, $CDCl_3$) of **1b**.

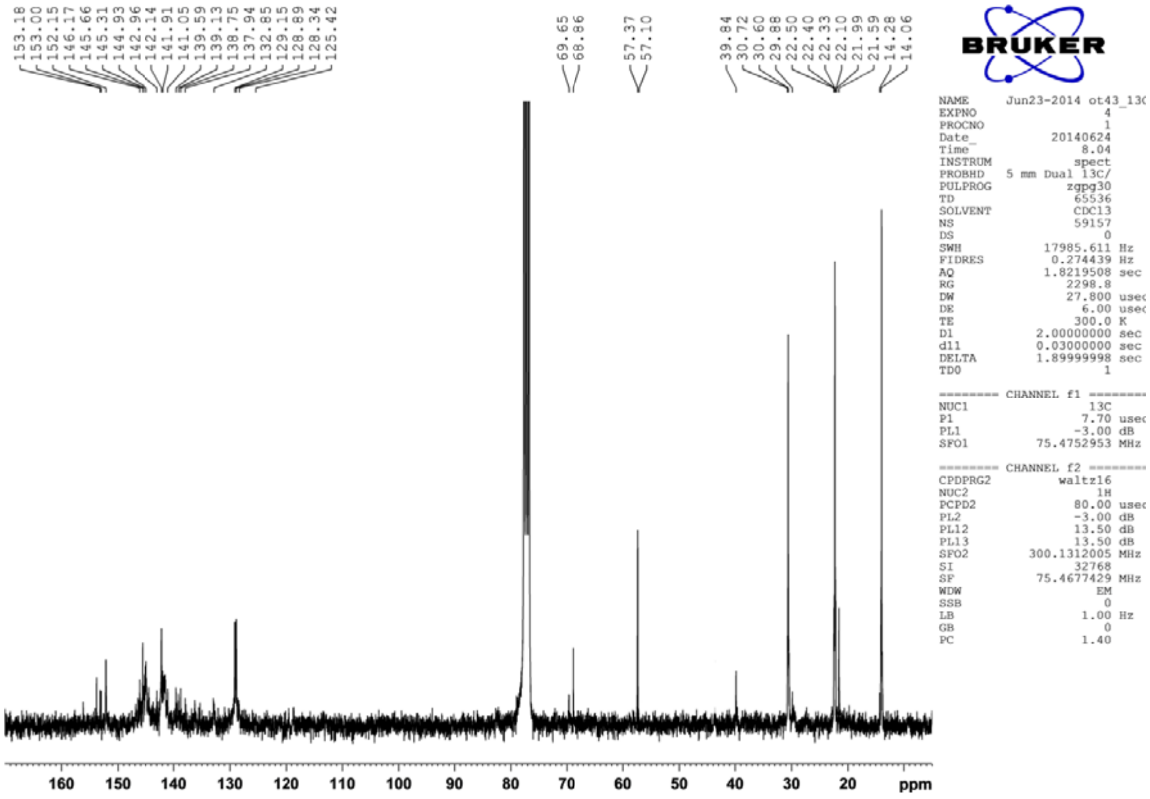

Figure S11: ^{13}C NMR spectrum (75.5 MHz, $CDCl_3$) of **1b**.

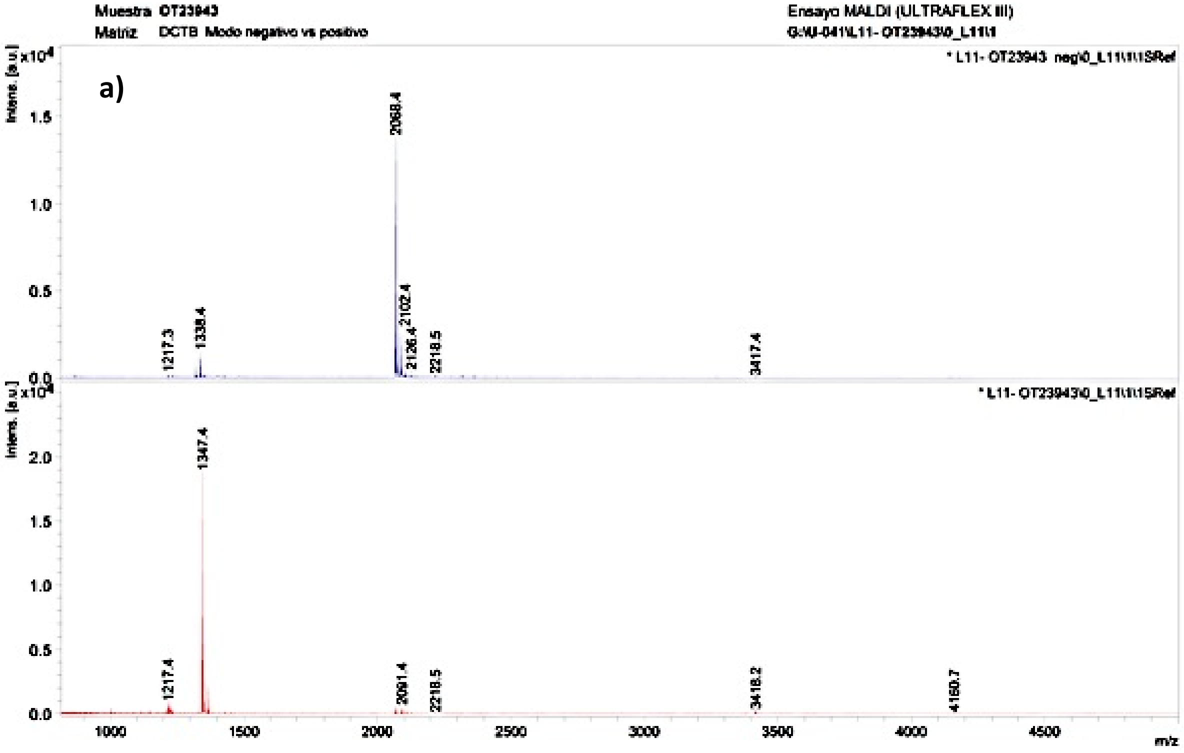

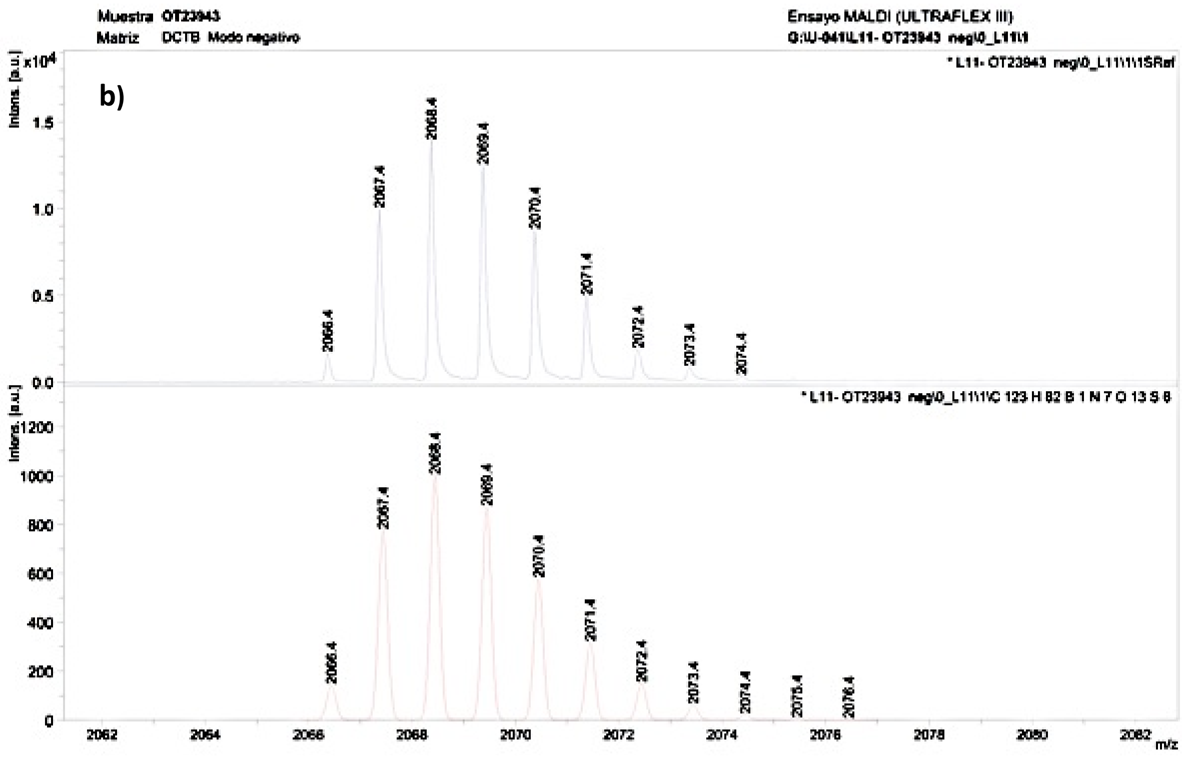

Figure S12: MALDI-TOF mass spectrum of **1b**, (DCTB) a)negative mode *vs* positive mode), b) isotopic distribution (negative mode), observed (top) *vs* calculated (bottom).

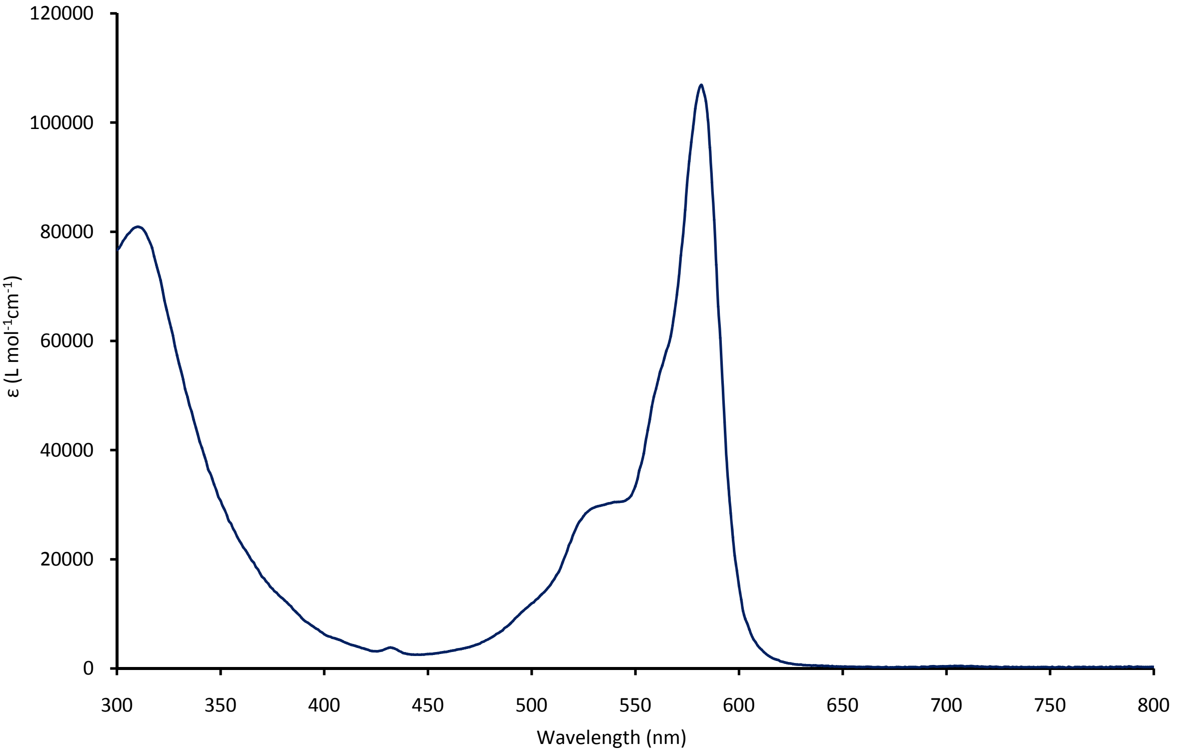

Figure S13: UV-vis spectrum of **1b** in toluene.

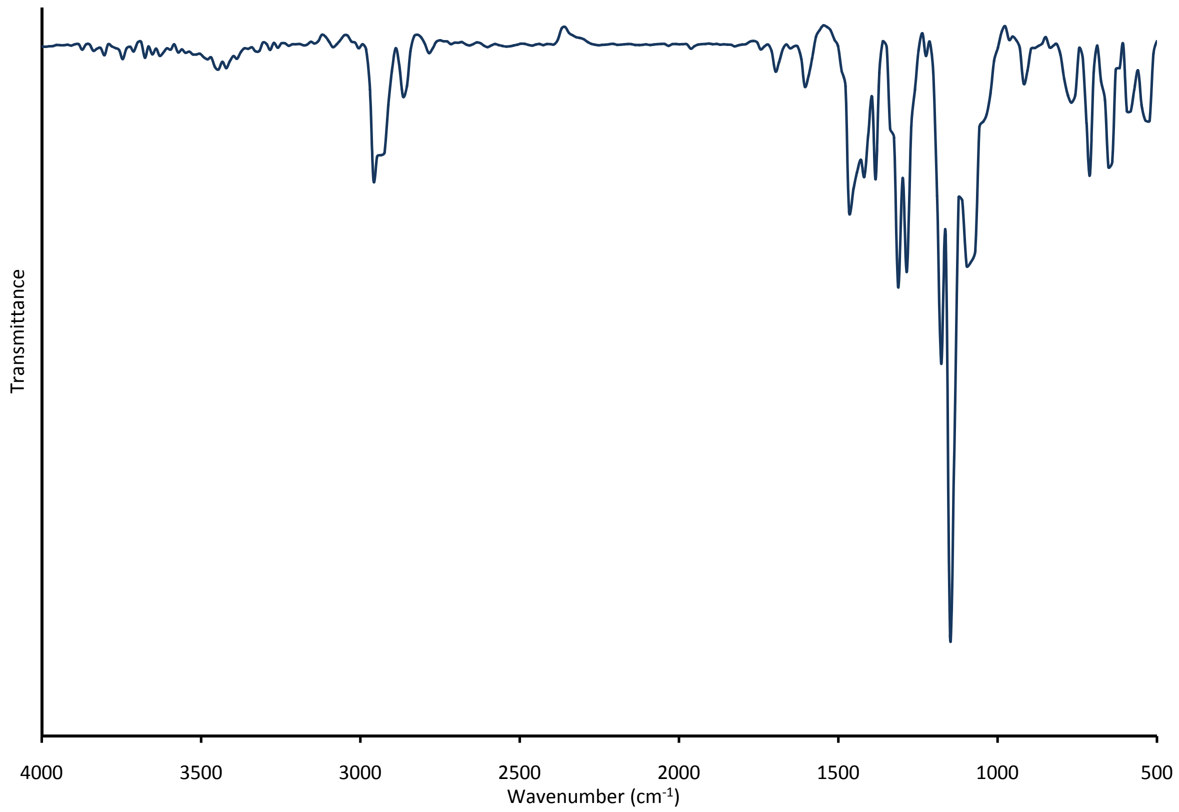

Figure S14: IR spectrum of **1b** (KBr).

- 3’-Formylphenoxy-[1,2,3,4,8,9,10,11,15,16,17,18-dodecafluorosubphthalocyaninato]boron(III) **2a**

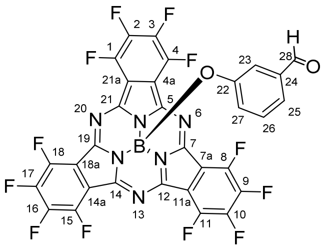

A mixture of F_{12}SubPcCl**3a** (130 mg, 0.2mmol) andsilver (I) trifluoromethanesulfonate(AgOTf)(77 mg, 0.3mmol) were stirred in dry toluene (3 mL) at room temperature under argon atmosphere protected from light. After 12 h, when no unreacted**3a** was detected by TLC, 3-hydroxybenzaldehyde (49 mg, 0.4 mmol) was added under argon flow, following by the addition of N,N-diisopropylethylamine (DIPEA) (52 µL, 0.3 mmol) via syringe. The stirring was continued at 80 °C under argon atmosphere, and stopped after 4 h when no intermediate SubPcOTf was observed by TLC.[a]The reaction mass was diluted with 20 mL of toluene, passed through celite pad to eliminate impurities of inorganic salts, and thoroughly collected with toluene. Resulted solution was reduced in volume to approx. 8mL and charged onto silica gel column. Elution with a mixture of toluene-ethylacetate = 3:1 (vol) and evaporation of solvents resulted in a dark brown residue that was suspended in hexane, filtered, dried in vacuum, yielding 125 mg of **2a** (85%). R_f = 0.5 (hexane-ethylacetate = 6:1, vol).

^{1}H NMR (300 MHz, $CDCl_3$): δ (ppm) = 9.59 (s, 1H; H-28), 7.20 (dd, J_o = 7.6 Hz, J_m = 1.6 Hz, 1H; H-25), 6.98 (dd, J_o = 7.6 Hz, $J_{o'}$ = 8.2 Hz, 1H; H-26), 5.91 (dd, J_m=$J_{m'}$ = 1.5 Hz, 1H; H-23), 5.56 (dd, $J_{o'}$ = 8.2 Hz, J_m = 1.6 Hz, 1H; H-27); ^{13}C NMR (75.5 MHz, $CDCl_3$): δ (ppm) = 191.3 (1C; C-28), 152.2 (1C; C-22), 148.6 (6C; C-5, C-7, C-12, C-14, C-19, C-21), 144.7-144.4 (m, 6C; C-F), 141.3-140.9 (m, 6C; C-F), 137.8 (1C; C-24), 130.2 (1C; C-26), 124.94, 124.87 (2C; C-25, C-27), 119.1 (1C; C-23), 115.1-114.6 (m, 6C; C-4a, C-7a, C-11a, C-14a, C-18a, C-21a); MALDI-TOF MS (positive mode, DCTB): found: 732.1 *m/z,* calc. for $C_{31}H_5B_1F_{12}N_6O_2$, $[M]^+$: 732.0; IR (KBr): ν (cm^{-1}) = 2947, 1697 (C=N), 1533, 1483 (C-F), 1387, 1269, (C-N), 1221, 1163, 1115, 1088 (B-O), 970, 714, 609; UV-vis (toluene): λ_{max} (nm) (log ε) = 574 (4.9), 558 (sh), 534 (4.4), 520 (sh), 499 (sh), 306 (4.5).

[a]SubPcs with electron-withdrawing groups are known to require longer reaction time and higher reaction temperatures in reactions of nucleophilic substitution at boron atom, compared to non-substituted SubPcs[1].

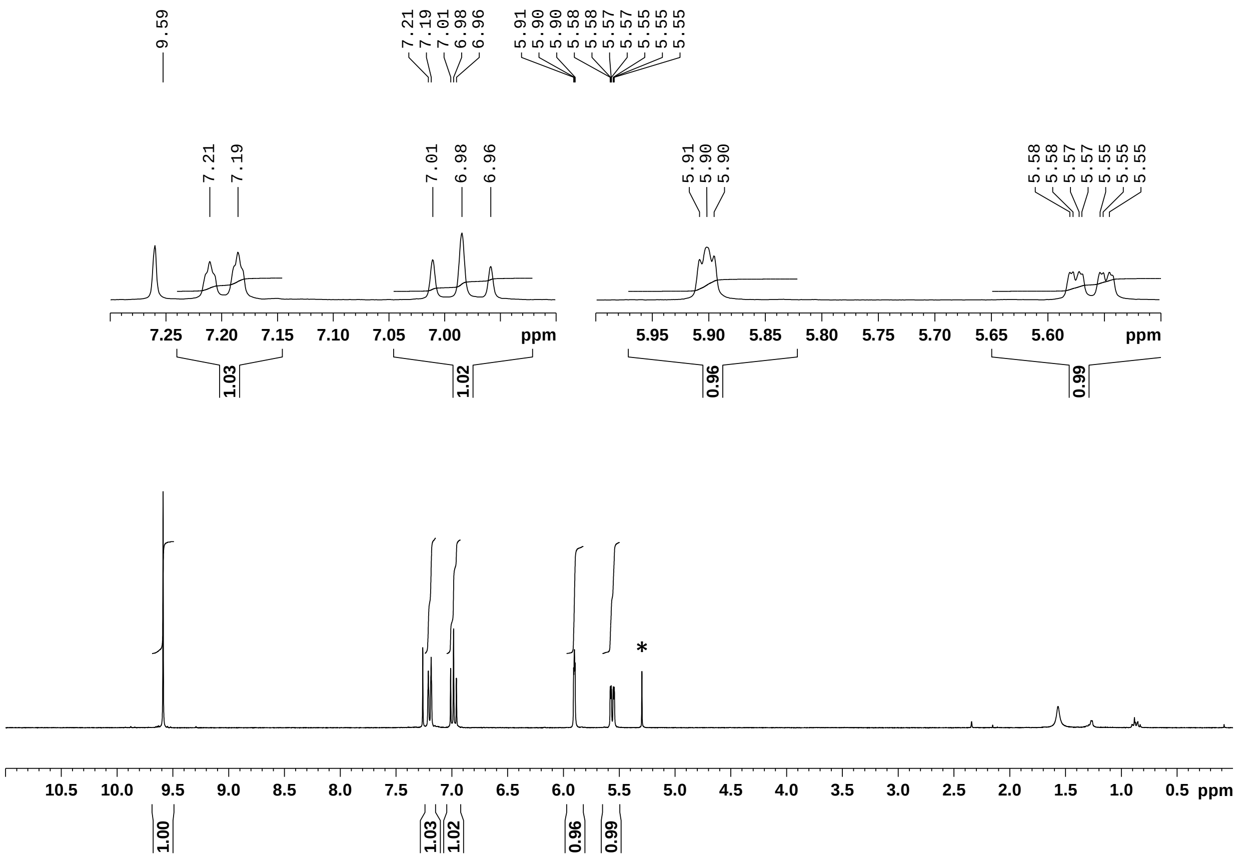

(*impurity of dichloromethane)

Figure S15: ^{1}H NMR spectrum (300 MHz, $CDCl_3$) of **2a**.

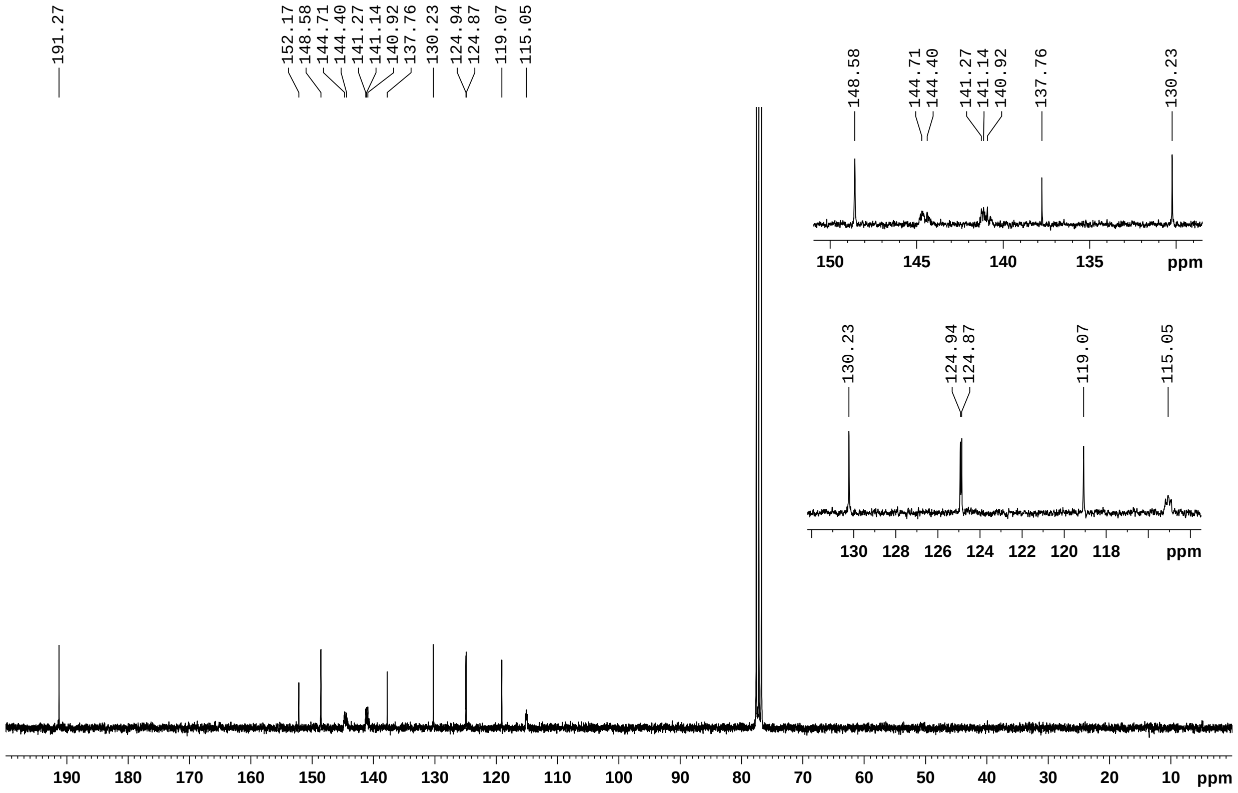

Figure S16: ^{13}C NMR spectrum (75 MHz, $CDCl_3$) of **2a**.

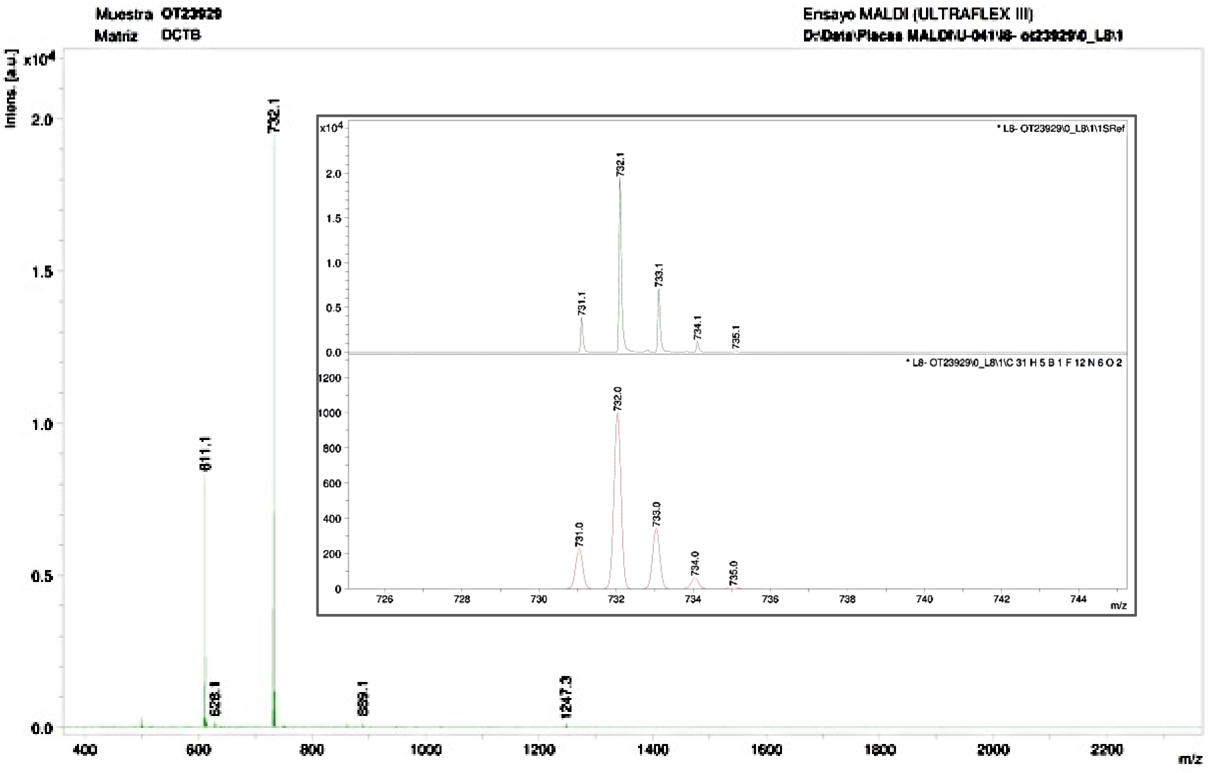

Figure S17: MALDI-TOF mass spectrum of **2a**, (DCTB,positive mode) (inserted graph) isotopic distribution: observed (top) *vs* calculated (bottom).

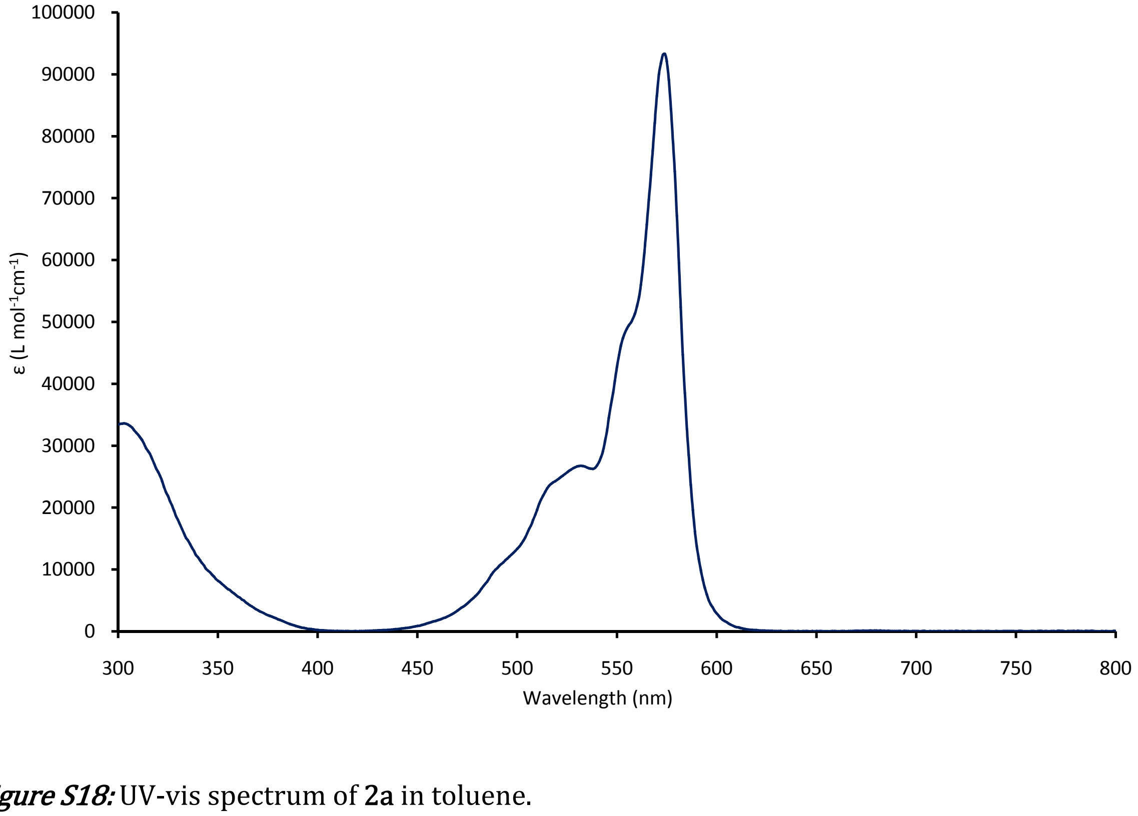

Figure S18: UV-vis spectrum of **2a** in toluene.

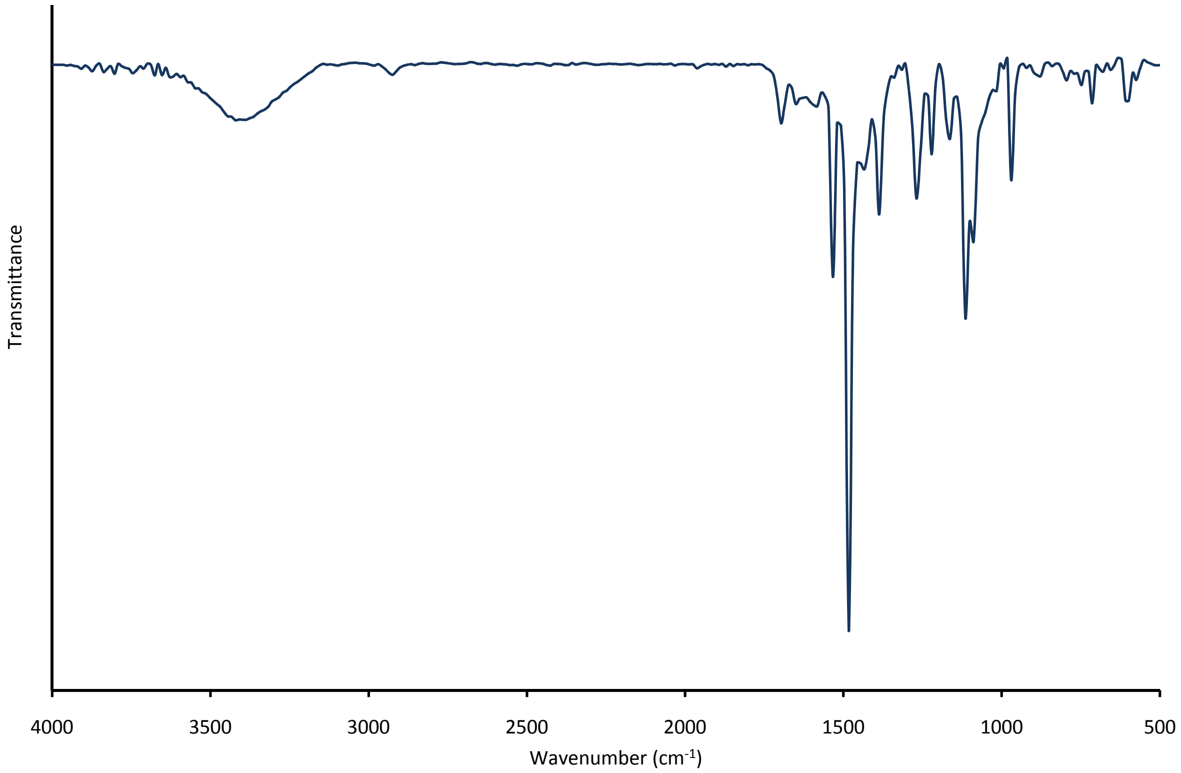

Figure S19: IR spectrum of **2a** (KBr).

- 3’-Formylphenoxy-[2,3,9,10,16,17-hexa(pentylsulfonyl)-subphthalocyaninato]boron(III) **2b**

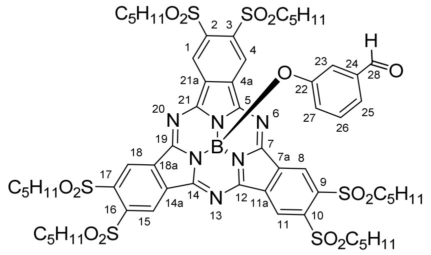

A mixture of $(SO_2C_5H_{11})_6$SubPcCl**3b** (865 mg, 0.7mmol) and AgOTf(257 mg, 1.0mmol) were stirred in dry toluene (10 mL) at room temperature under argon atmosphere protected from light. After 12 h, some unreacted **3b**could be still detected by TLC.Nevertheless, 3-hydroxybenzaldehyde (171 mg, 1.4 mmol) was added under argon flow, following by the addition of DIPEA (174 µL, 1.0mmol) via syringe. The stirring was continued at 115°C under argon atmosphere, and stopped after 1 h when no unreacted **3b**orintermediate SubPcOTf were observed by TLC.[b] The reaction mass was diluted with 20 mL of dichloromethane, passed through celite pad, and thoroughly collected with dichloromethane. Resulted solution was rotary evaporated till dryness. The residue was dissolved in 25 mL of dichloromethane and charged onto a silica gel column. Dichloromethane containing 0.1% of ethanol was used as an eluting solvent, affording the compound as a main zone. Additional purification of the compound on Bio-Beads, eluting with toluene, allowed pure **2b**, allowing the separation of the main compound from(sulfonyl)SubPcdecomposition products formed on silica gel. Evaporation of solvents resulted in a magneta-colorsolid that was suspended in hexane, filtered, dried in vacuum, yielding 125 mg of **2b** (25%). R_f = 0.5 (dichloromethane-ethanol, 0.1 vol %).

^{1}H NMR (300 MHz, $CDCl_3$): δ (ppm) = 9.82 (s, 6H; H-1, H4, H-8, H-11, H-15, H-18), 9.66 (s, 1H; H-28), 7.23 (dd (br), 1H; H-25), 7.01 (dd, J_o = 8.2 Hz, $J_{o'}$ = 8.6 Hz, 1H; H-26), 5.93 (dd (br), J_m = 7.6 Hz, $J_{m'}$ = 1.6 Hz, 1H; H-23), 5.59 (dd, J_o = 8.2 Hz, J_m = 1.6 Hz, 1H; H-27); ^{13}C NMR (75.5 MHz, $CDCl_3$): δ (ppm) = 191.4 (1C; C-28), 152.31 (6C; C-5, C-7, C-12, C-14, C-19, C-21), 152.28 (1C; C-22), 141.96 (6C; C-2, C-3, C-9, C-10, C-16, C-17), 138.02 (1C; C-24), 132.89 (6C; C-4a, C-7a, C-11a, C-14a, C-18a, C-24a), 130.49 (1C; C-26), 129.13 (2C; C-25), 125.48 (6C; C-1, C-4, C-8, C-11, C-15, C-18), 125.05 (1C; C-27), 118.57 (2C; C-23), 57.49 (6C; SO_2CH_2), 30.66, 22.60, 22.39 (18C; $SO_2CH_2(CH_2)_3$), 14.04 (6C; CH3); MALDI-TOF MS (positive mode, DCTB): found: 1320.4 *m/z,* calc. for $C_{61}H_{77}B_1N_6O_{14}S_6$, $[M]^+$: 1320.4; IR (KBr): ν (cm^{-1}) = 2959, 2935 (C-H), 1697, 1616 (C=C), 1466, 1385, 1315, 1292, 1177 (S=O), 1140, 1068 (B-O), 918, 710, 652;UV-vis (toluene): λ_{max} (nm) (log ε) = 581 (5.1), 566 (sh), 536 (4.5), 311 (4.7).

[b] Presence of six electron-withdrawing sulfonylpentoxy groups on the periphery of SubPc**3b** hampers nucleophilic substitution of the axial group at the boron atom even stronger than twelve fluorine substituents of **3a**, causing the need of using of high temperatures. Worthy to note, that the reaction proceeds as well at lower temperatures during longer times, giving lower yields of the product of nucleophilic substitution and higher amount of side- and decomposition products.

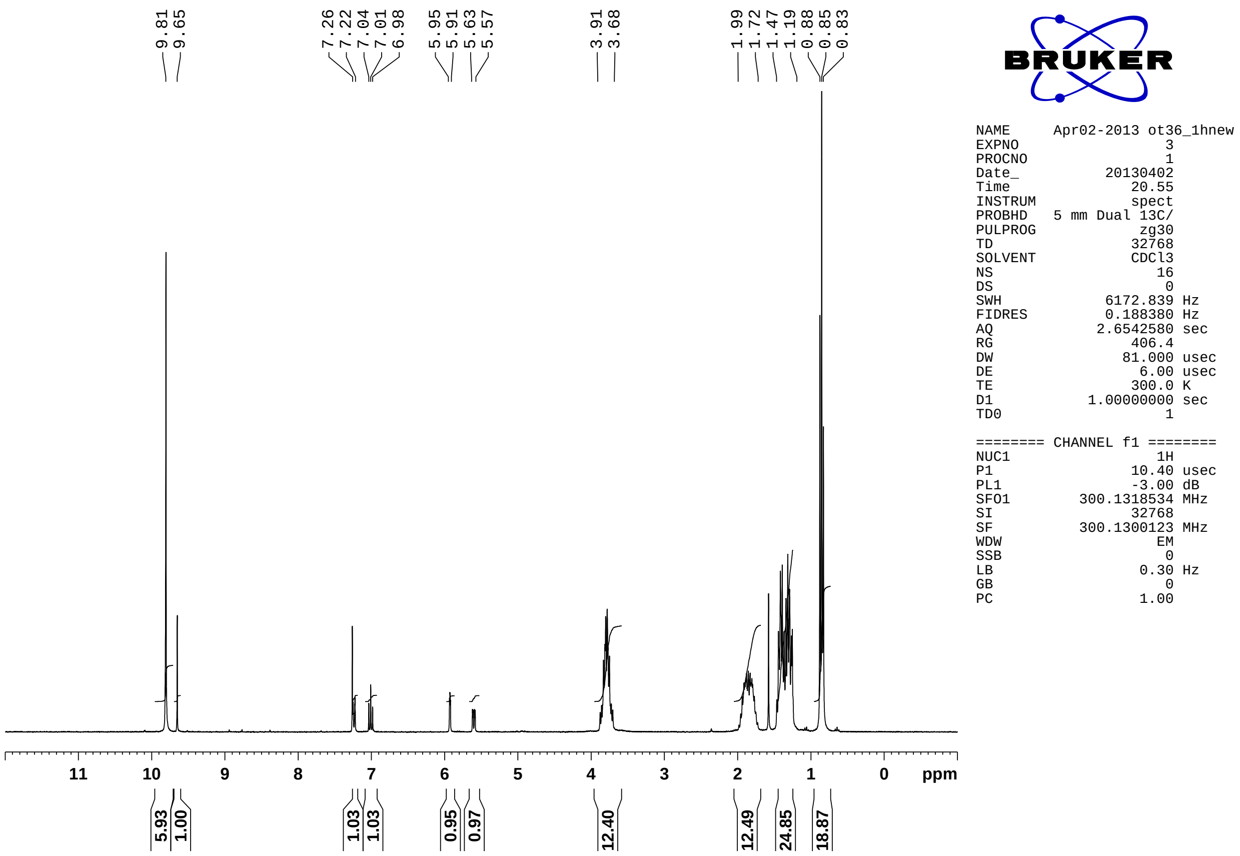

Figure S20: ^{1}H NMR spectrum (300 MHz, $CDCl_3$) of **2b**.

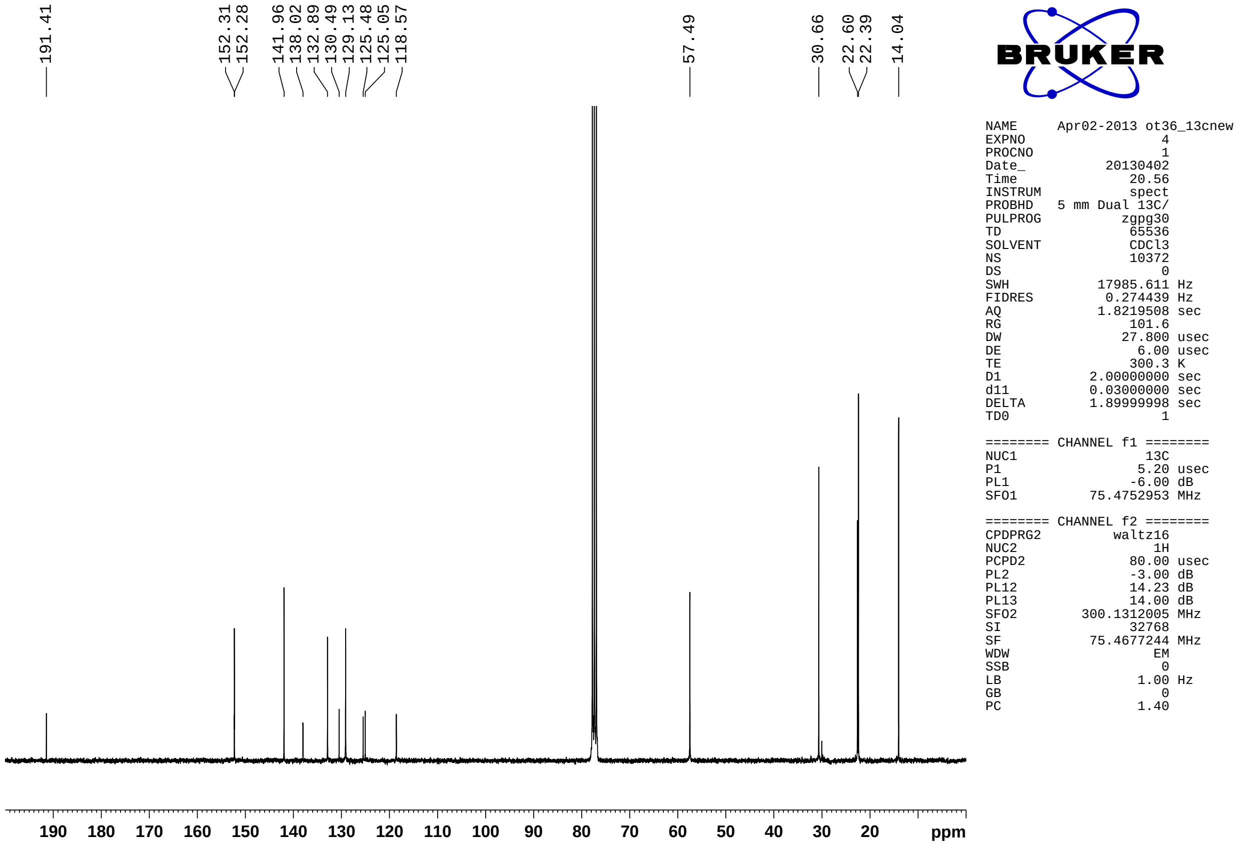

Figure S21: ^{13}C NMR spectrum (75 MHz, $CDCl_3$) of **2b**.

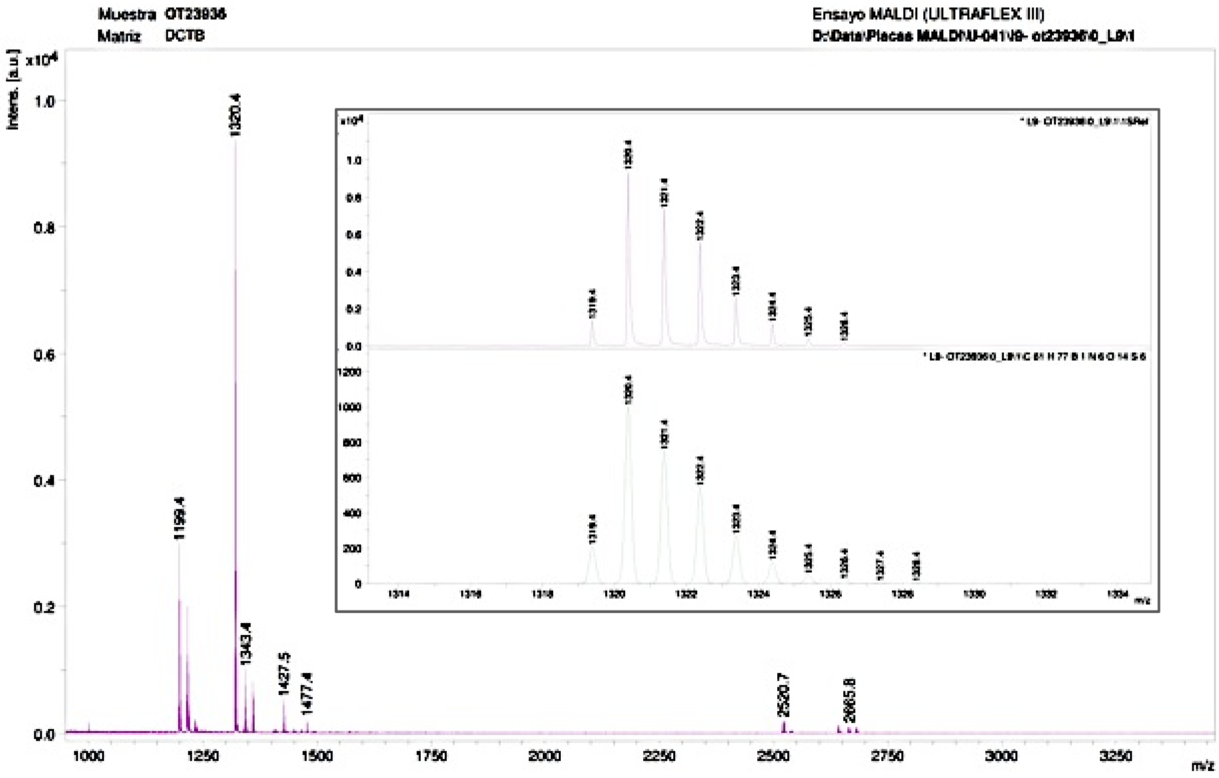

Figure S22: MALDI-TOF mass spectrum of **2b**, (DCTB,positive mode).

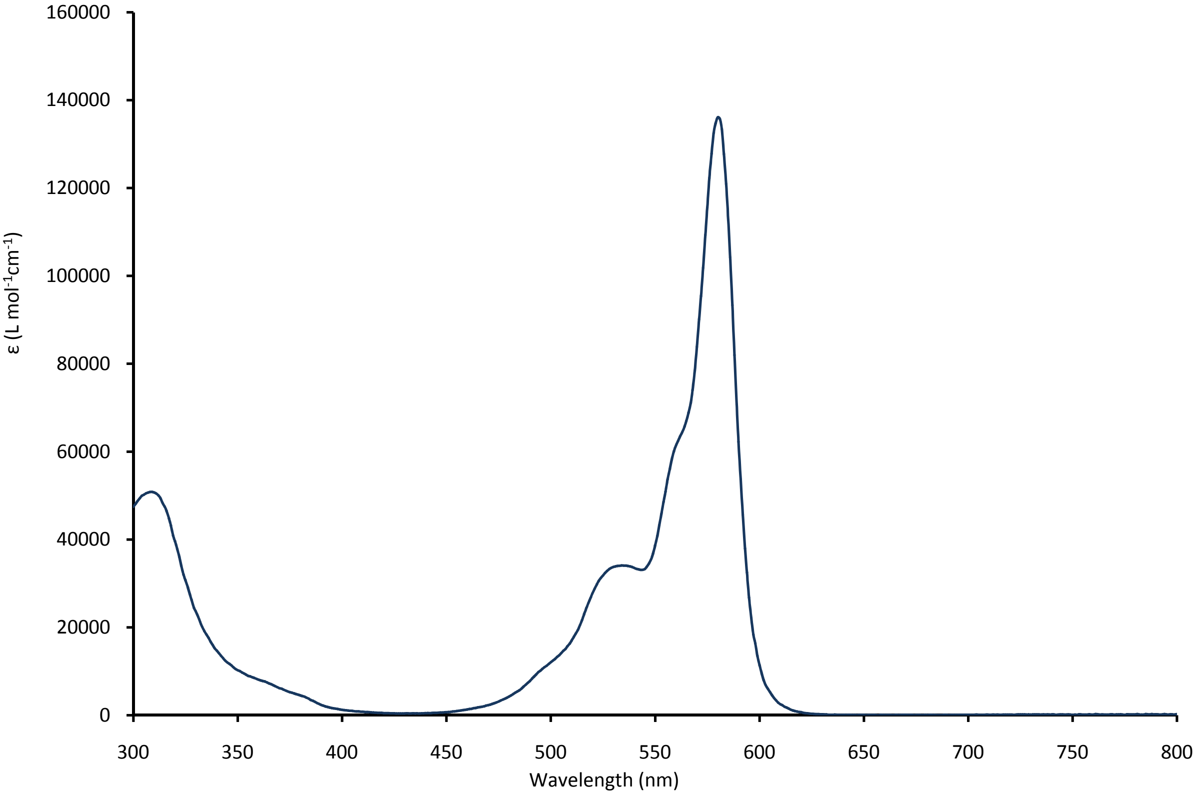

Figure S23: UV-vis spectrum of **2b** in toluene.

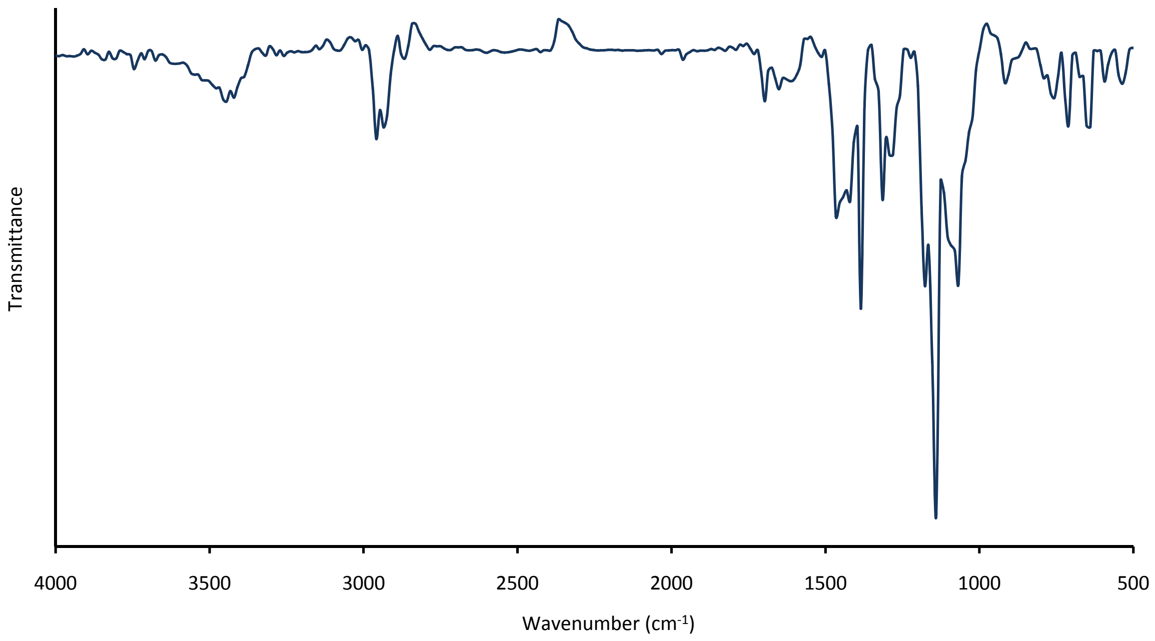

Figure S24: IR spectrum of **2b** (KBr).

- Phenoxy-[2,3,9,10,16,17-hexa(pentylsulfonyl)-subphthalocyaninato]boron(III) **2d**

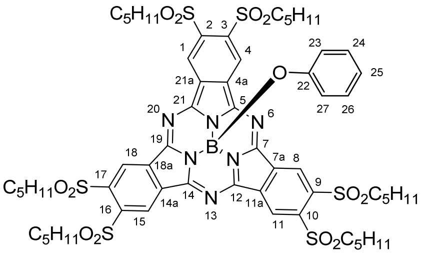

A mixture of $(SO_2C_5H_{11})_6$SubPcCl**3b** (494 mg, 0.4mmol) and AgOTf (154 mg, 0.6mmol) were stirred in dry toluene (7 mL) at room temperature under argon atmosphere protected from light. After 12 h, some unreacted **3b** could be still detected by TLC. Nevertheless, phenol (75 mg, 0.8mmol) was added under argon flow, following by the addition of DIPEA (91 μL, 0.6mmol) via syringe. The stirring was continued at 115 °C under argon atmosphere, and stopped after 1 h when no unreacted **3b** or intermediate SubPcOTf were observed by TLC. The reaction mass was diluted with 20 mL of dichloromethane, passed through celite pad, and thoroughly collected with dichloromethane. Resulted solution was rotary evaporated till dryness. The residue was dissolved in 15 mL of dichloromethane and charged onto a silica gel column. Dichloromethane containing 0.1% of ethanol was used as an eluting solvent, affording the compound as a main zone. Additional purification of the compound on Bio-Beads, eluting with toluene, allowed pure **2d**, allowing the separation of the main compound from (sulfonyl)SubPc decomposition products formed on silica gel. Partial evaporation of a solvent and further precipitation of the compound with hexane afforded the compound in the form of magneta-color single crystals suitable for X-Ray diffraction analysis. Crystalline solid was filtered from hexane, dried in vacuum, yielding 181 mg of **2d** (35%). $R_f = 0.8$ (dichloromethane-ethanol, 0.1 vol %).

^{1}H NMR (300 MHz, $CDCl_3$): δ(ppm) = 9.80 (s, 6H; H-1, H-4, H-8, H-11, H-15, H-18), 6.82 (dd, J_o = 7.1 Hz, $J_{o'}$ = 7.6 Hz, 2H; H-24), 6.73 (t, J_o = 7.6 Hz, 1H; H-25), 5.36 (d, J_o = 7.1 Hz, 2H; H-23), 3.90-3.70 (m, 12H; SO_2CH_2), 2.0-1.73 (m, 12H; SO_2CH_2**CH_2**), 1.47-1.25 (m, 24H; $SO2(CH_2)_2$(**CH_2**)$_2$), 0.88-0.83 (m, 18H; CH_3);^{13}C NMR (75.5 MHz, $CDCl_3$): δ(ppm) = 152.19 (6C; C-5, C-7, C-12, C-14, C-19, C-21), 151.45 (1C; C-22), 141.81 (6C; C- 2, C-3, C-9, C-10, C-16, C-17), 132.94 (6C; C-4a, C-7a, C-11a, C-14a, C-18a, C-24a), 129.81 (2C; C-24), 129.08 (6C; C-1, C-4, C-8, C-11, C-15, C-18), 123.11 (1C; C-25), 118.95 (2C; C-23), 57.51 (6C; SO_2CH_2), 30.67, 22.64, 22.40 (18C; SO_2CH_2(**CH_2**)$_3$), 14.03 (6C; CH_3);MALDI-TOF MS (positive mode, DCTB): found: 1292.4 *m/z,* calc. for $C_{60}H_{77}B_1N_6O_{13}S_6$, $[M]^+$: 1292.4; HR MALDI-TOF MS (DCTB, PPGNa1000, PPGNa2000) found: 1292.3963 *m/z,* calc. for $C_{60}H_{77}B_1N_6O_{13}S_6$, $[M]^+$: 1292.3971; found: 1315.3896 *m/z,* calc. for $C_{60}H_{77}B_1Na_1N_6O_{13}S_6$, $[M+Na]^+$: 1315.3868;IR (KBr): ν(cm^{-1}) = 2959, 2935 (C-H), 1744, 1593 (C=C), 1464, 1385, 1310, 1283, 1177, 1149 (S=O), 1097 (B-O), 710, 640, 536; UV-vis (toluene): λ_{max} (nm) (log ε) = 581 (5.1), 563 (sh), 534 (4.5), 505 (sh), 308 (4.7)

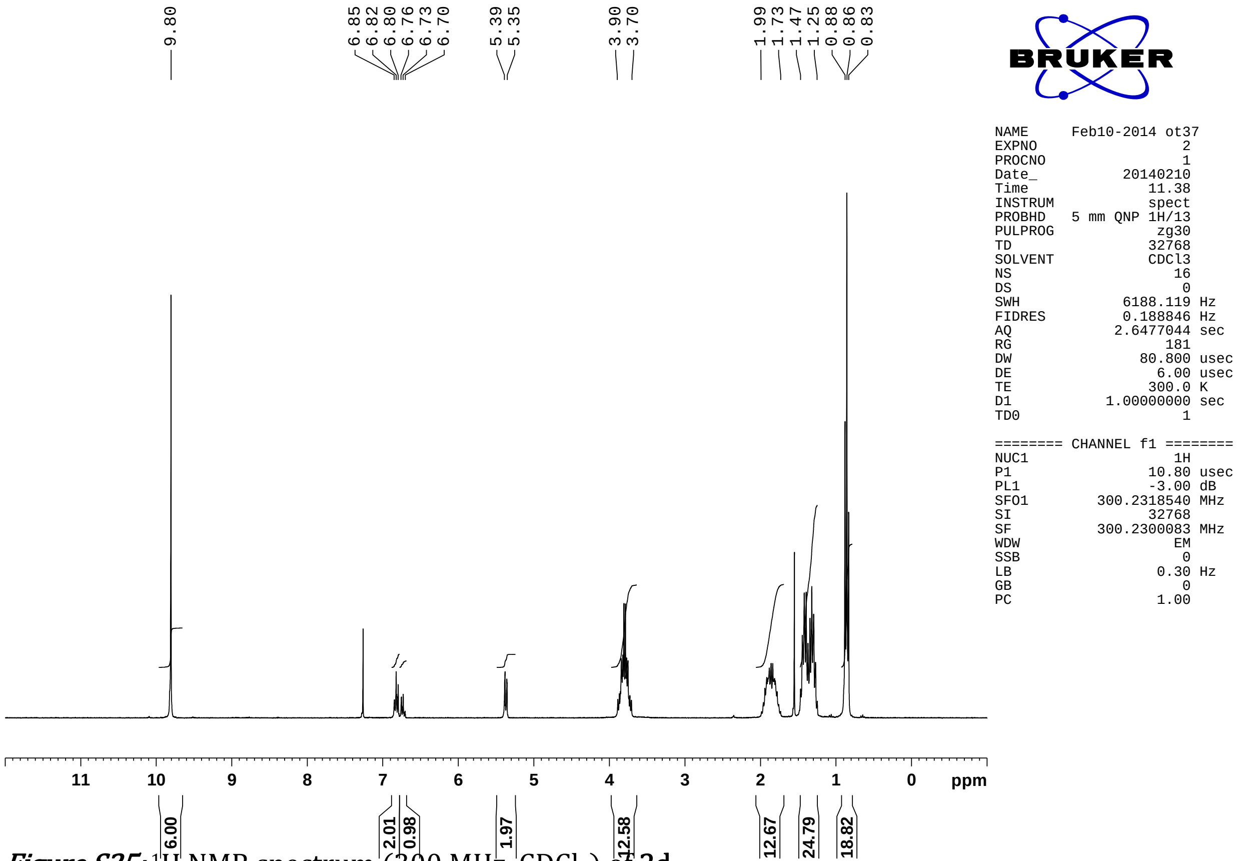

Figure S25: ^{1}H NMR spectrum (300 MHz, $CDCl_3$) of **2d**.

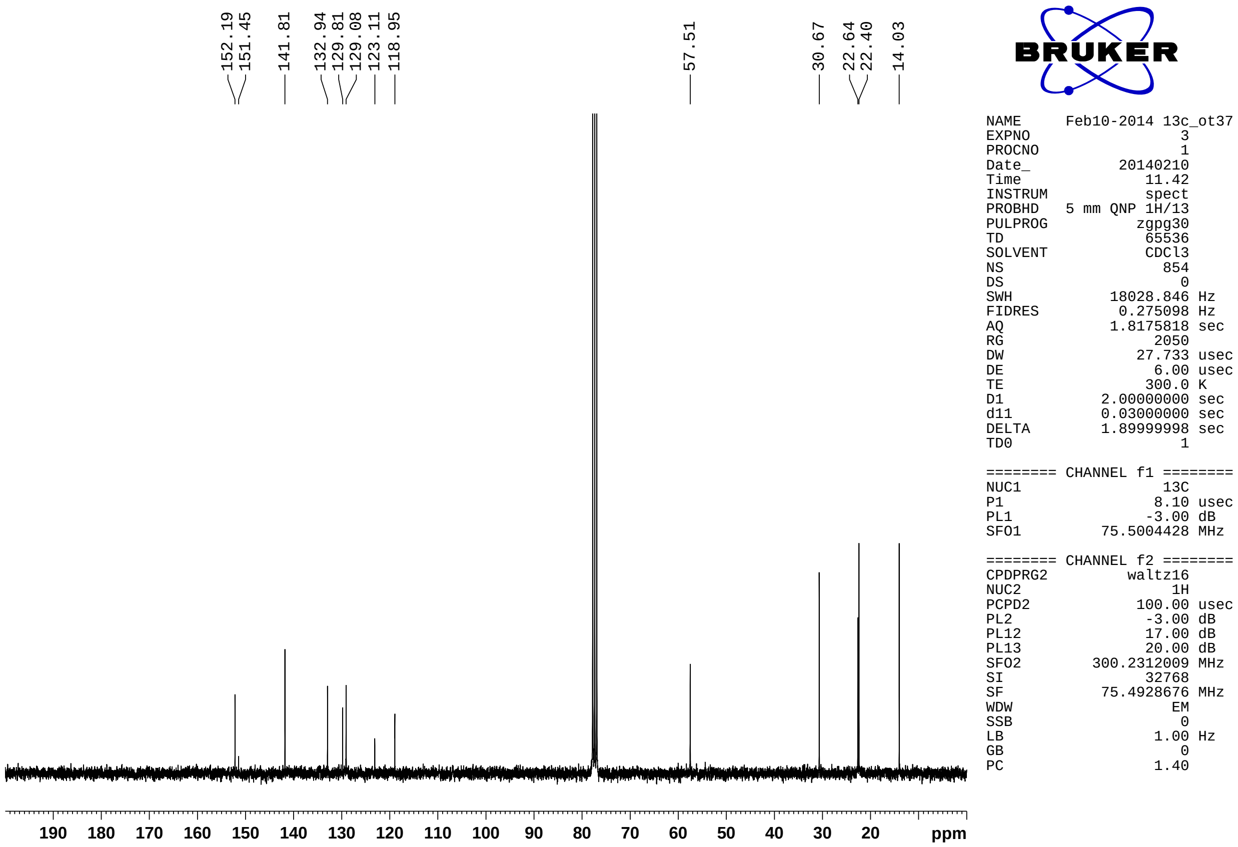

Figure S26: ^{13}C NMR spectrum (75 MHz, $CDCl_3$) of **2d**.

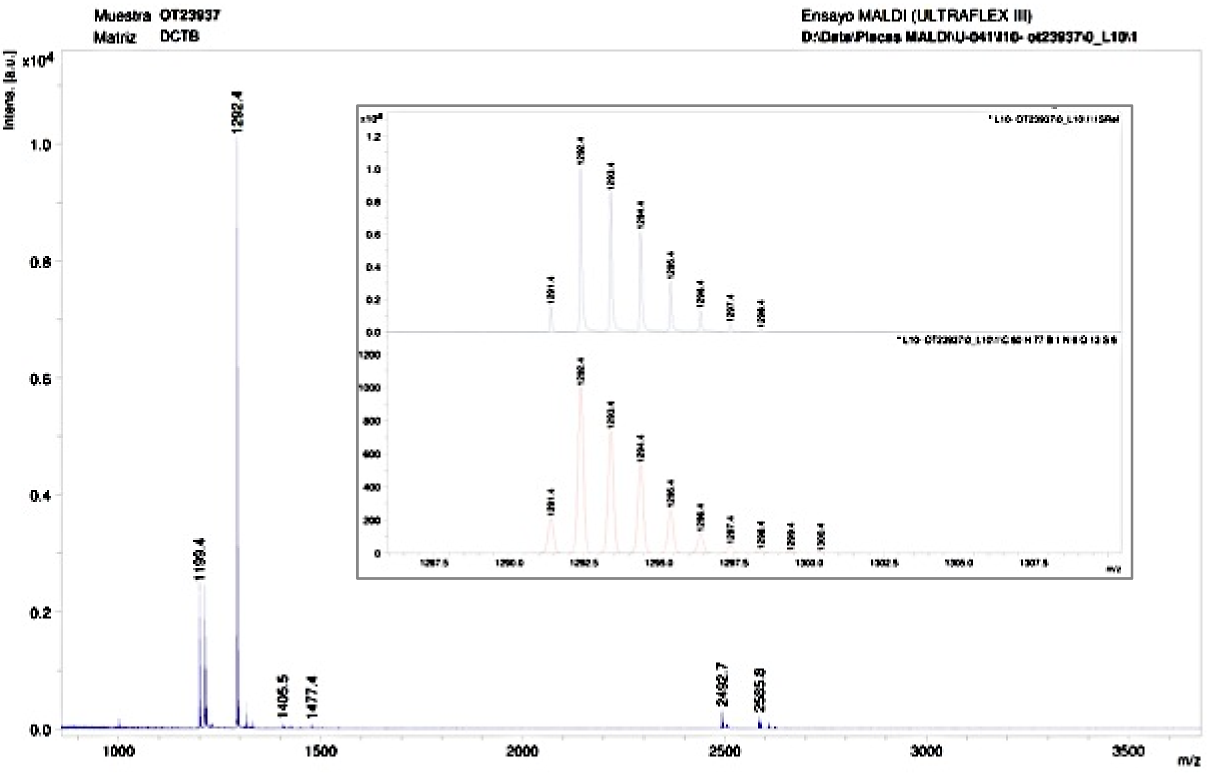

Figure S27: MALDI-TOF mass spectrum of **2d**, (DCTB,positive mode).

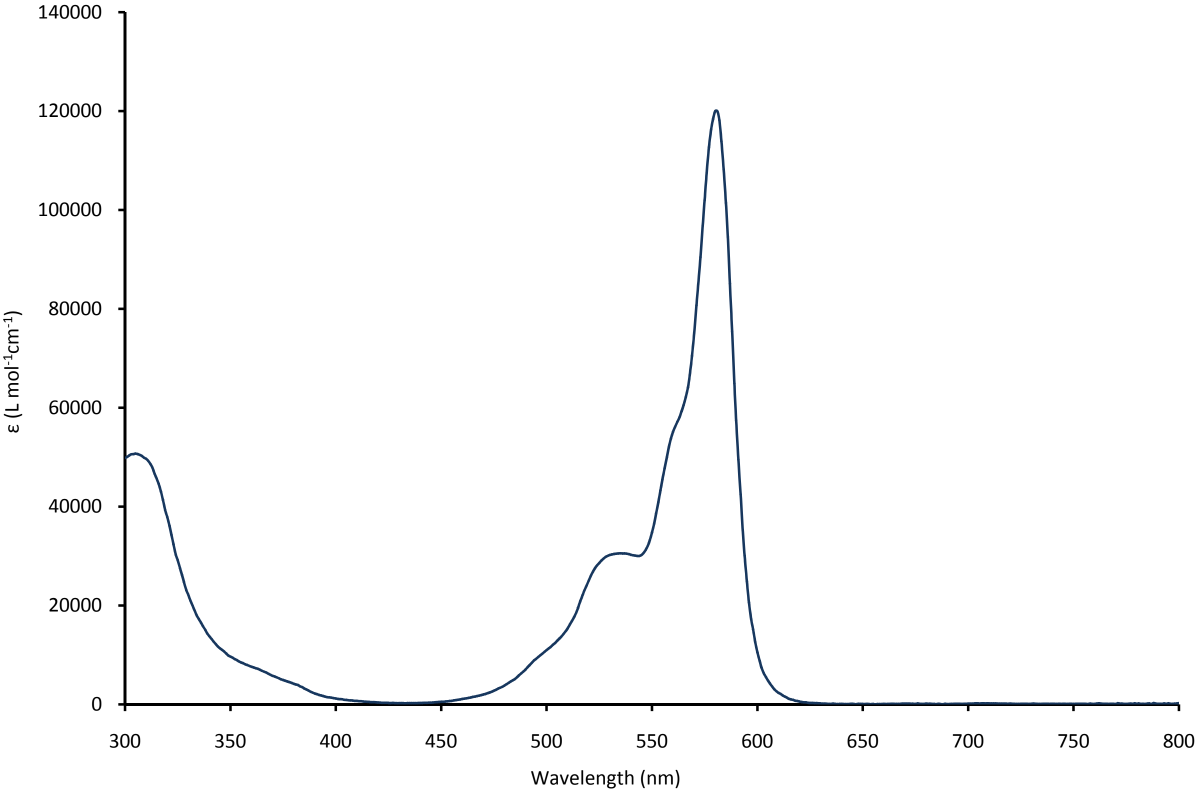

Figure S28: UV-vis spectrum of **2d** in toluene.

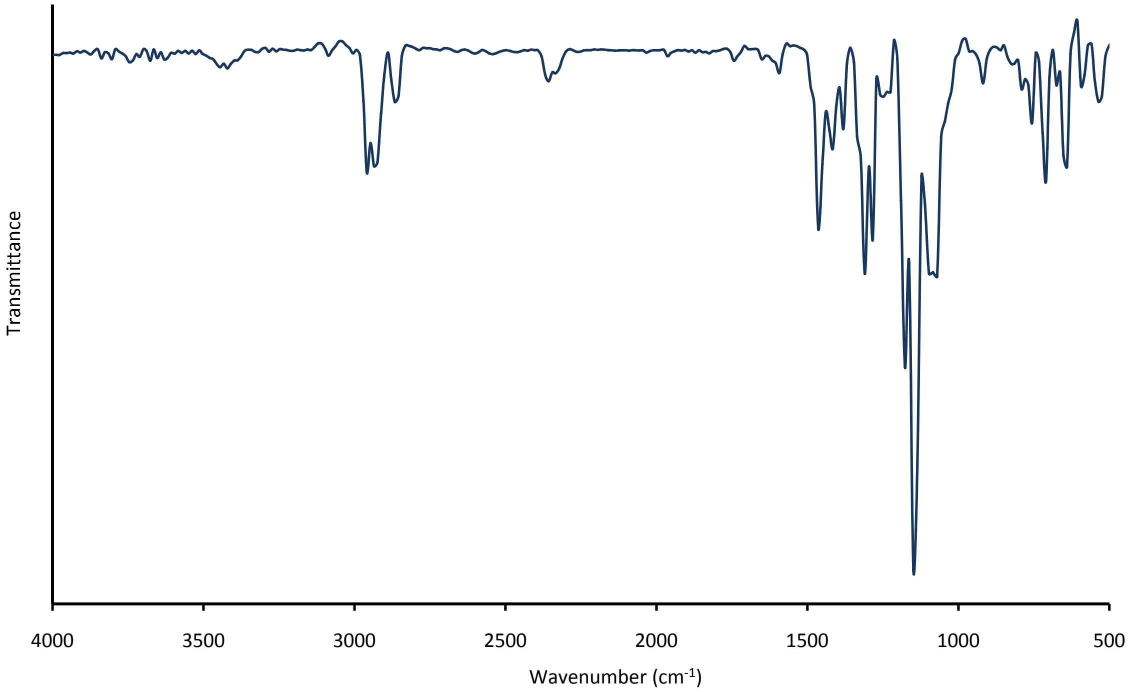

Figure S29: IR spectrum of **2d** (KBr).

- Chloro-[2,3,9,10,16,17-hexa(pentylsulfonyl)-subphthalocyaninato]boron(III) **3b**

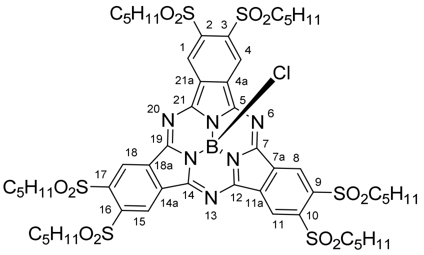

A 1M BCl_3 in *p*-xylene (10 mL, 10 mmol) was added to a1,2-dicyano-4,5-bis(pentylsulfonyl)benzene**5** (3.9 g, 10 mmol), and the reaction mixture was stirred vigorously at reflux for 30 min under argon atmosphere. Then the reaction was stopped, allowed to reach room temperature and flushed with argon. Xylene was rotary evaporated till dryness. The dark purple residuewas dissolved in10 mL of dichloromethane and subjected to column chromatography onsilica gel using a dichloromethane containing 0.5% of ethanol as an eluent. Rotary evaporation of solvents and precipitation of the product with hexane gave a dark blue solid that was filtered, washed with hexane, dried in vacuum, yielding the pure compound **3b** in an amount of 2.7 g of **3b** (65%). R_f = 0.8 (dichloromethane-ethanol, 0.1 vol %).

^{1}H NMR (300 MHz, $CDCl_3$): δ (ppm) = 9.86 (s, 6H; H-1, H-4, H-8, H-11, H-15, H-18), 3.92-3.72 (m, 12H; SO_2CH_2), 1.97-1.73 (m, 12H; SO_2CH_2**CH_2**), 1.47-1.25 (m, 24H; SO2$(CH_2)_2$(**CH_2**)$_2$), 0.88-0.83 (m, 18H; CH_3); ^{13}C NMR (75.5 MHz, $CDCl_3$): δ (ppm) = 150.70 (6C; C-5, C-7, C-12, C-14, C-19, C-21), 141.88 (6C; C-2, C-3, C-9, C-10, C-16, C-17), 132.81 (6C; C-4a, C-7a, C-11a, C-14a, C-18a, C-24a), 129.05 (6C; C-1, C-4, C-8, C-11, C-15, C-18), 57.31 (6C; SO_2CH_2), 30.47, 22.51, 22.20 (18C; SO_2CH_2(**CH_2**)$_3$), 13.84 (6C; CH_3); MALDI-TOF MS (positive mode, DCTB): found: 1234.3 *m/z,* calc. for $C_{54}H_{72}B_1Cl_1N_6O_{12}S_6$, $[M]^+$: 1234.3; IR (KBr): ν (cm^{-1}) = 2959, 2947 (C-H), 2866, 1744, 1466 (C=C), 1383, 1312, 1298, 1177, 1149 (S=O), 798, 706, 648, 532; UV-vis (toluene): λ_{max} (nm) (log ε) = 583 (5.0), 565 (sh), 539 (4.4), 315 (4.6).

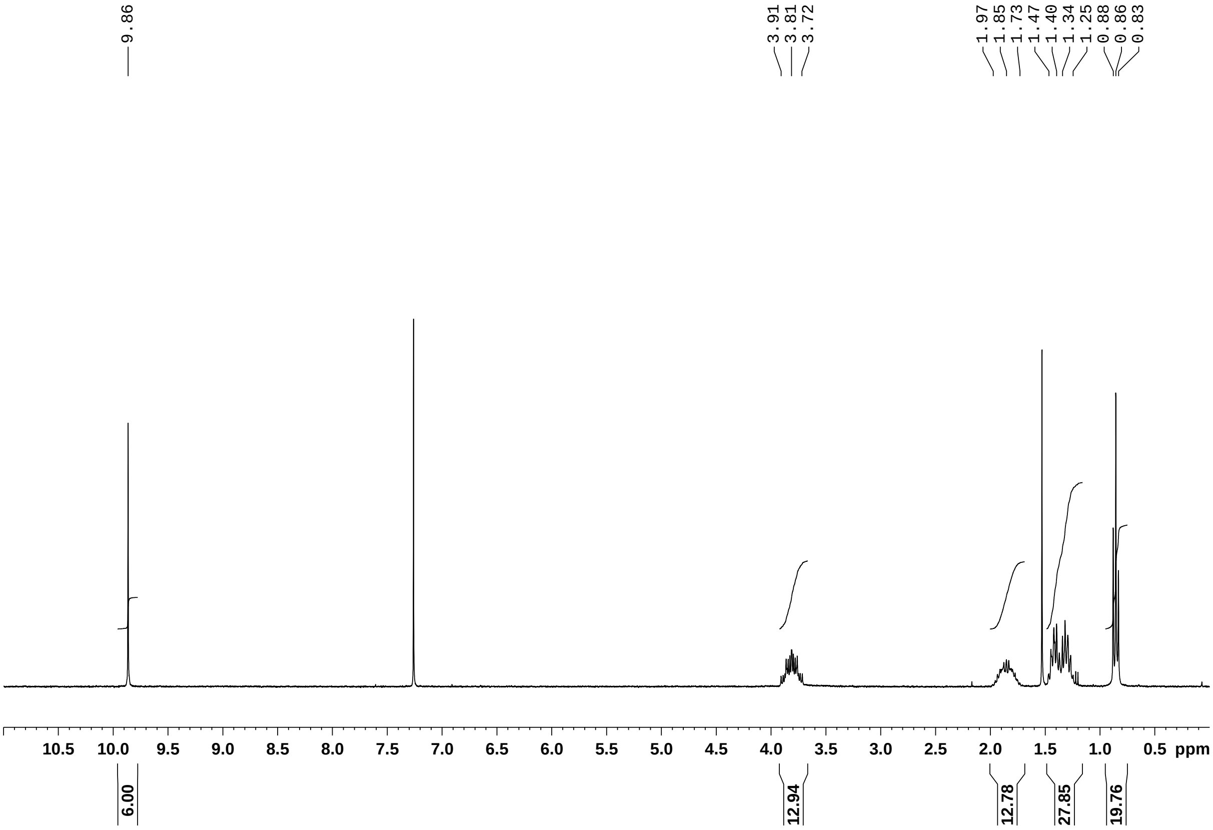

Figure S30: ^{1}H NMR spectrum (300 MHz, $CDCl_3$) of **3b**.

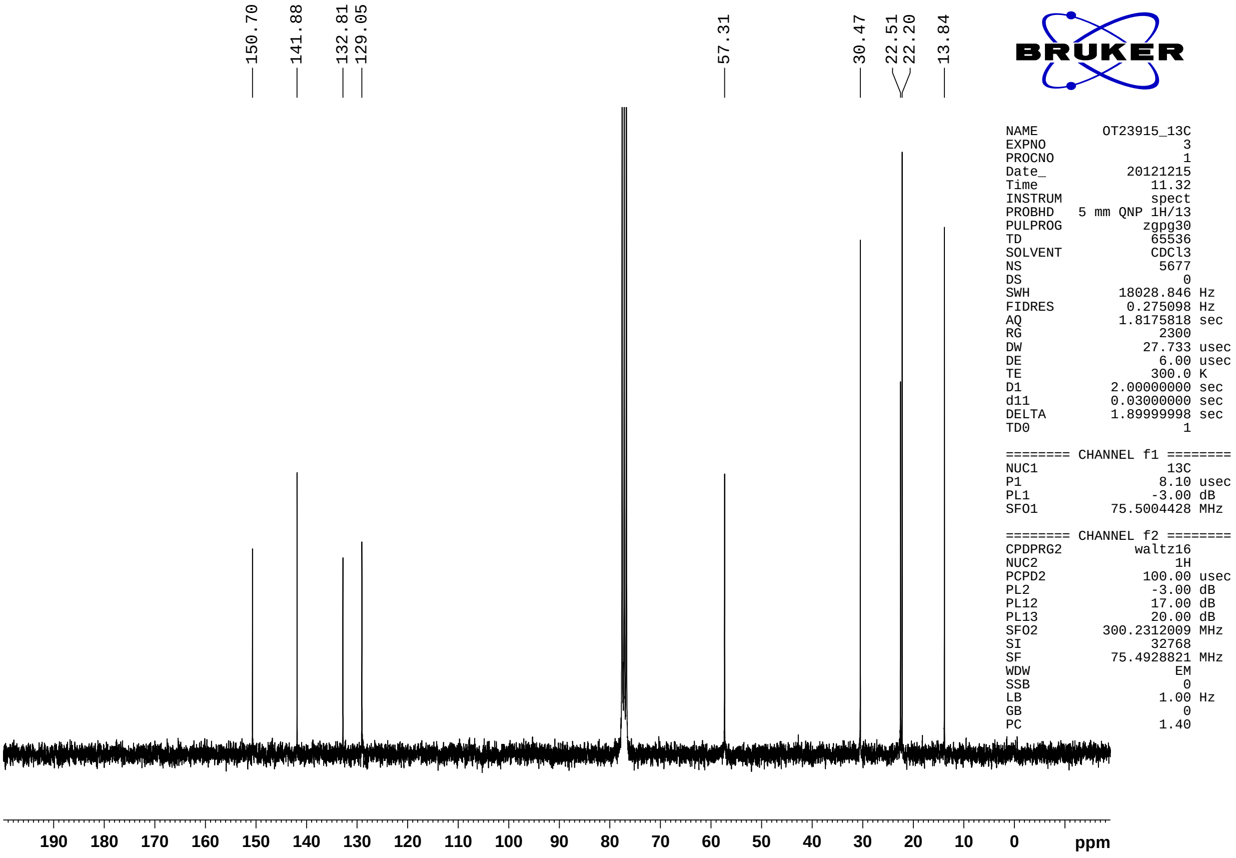

Figure S31: ^{13}C NMR spectrum (75 MHz, $CDCl_3$) of **3b**.

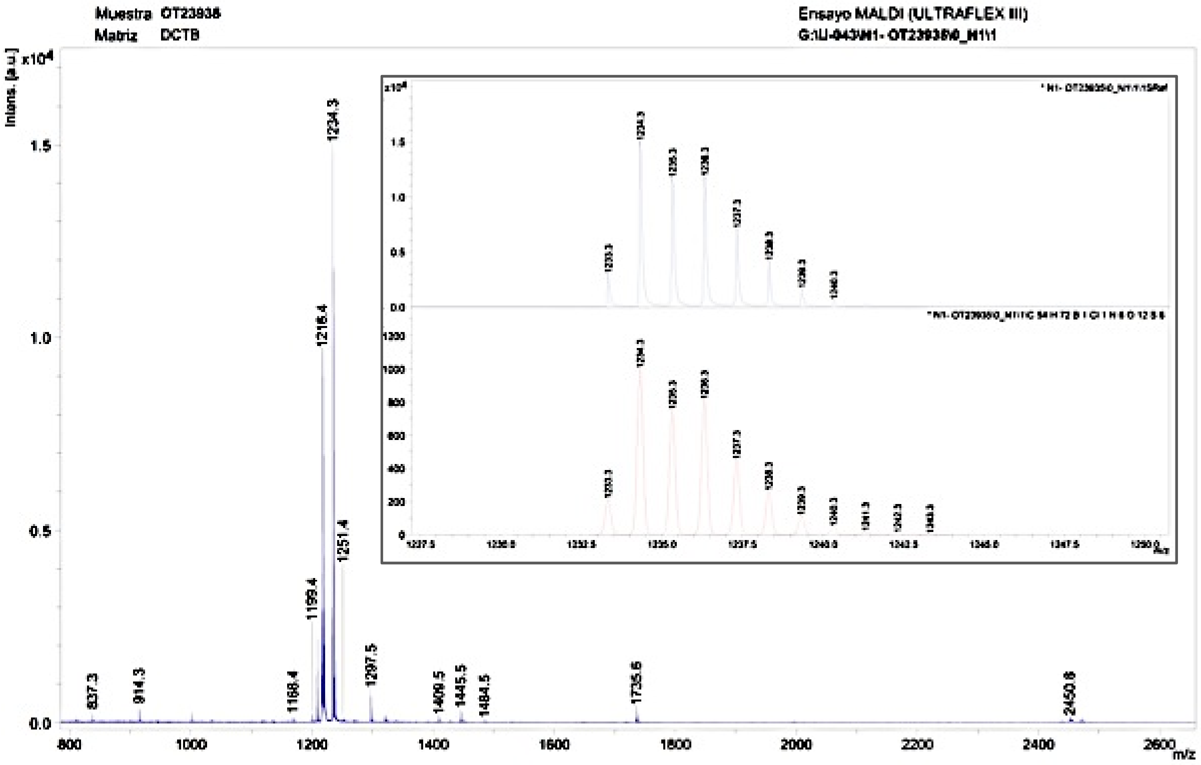

Figure S32: MALDI-TOF mass spectrum of **3b**, (DCTB,positive mode).[c]

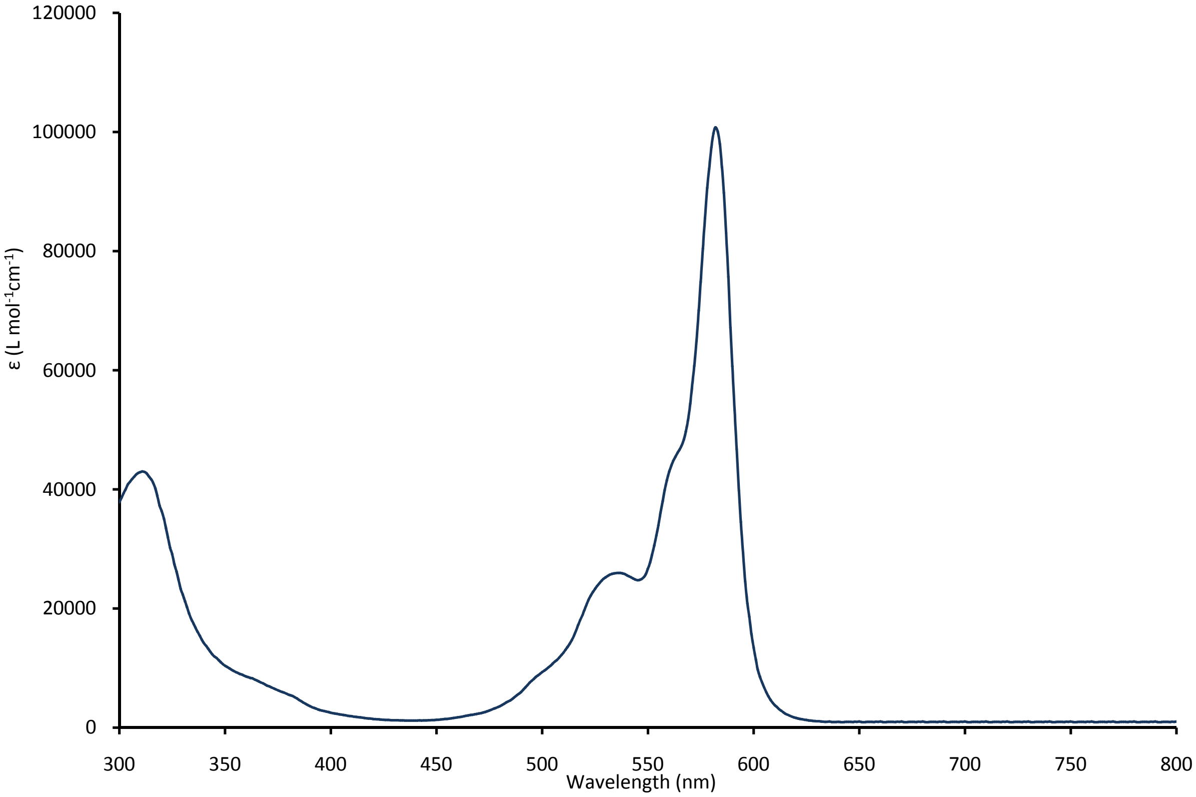

Figure S33: UV-vis spectrum of **3b** in toluene.

[c] The most intense peak corresponds to the $[M]^+$ ion, followed by the $[M - \text{axial group}]^+$ ion. Its observation in MS experiments (i.e. the loss of the axial group giving $SubPc^+$cation) together with the $[M - \text{axial group} + OH]^+$ ion (i.e. $SubPcOH^+$) is common for chlorosubstitutedSubPcs[1].

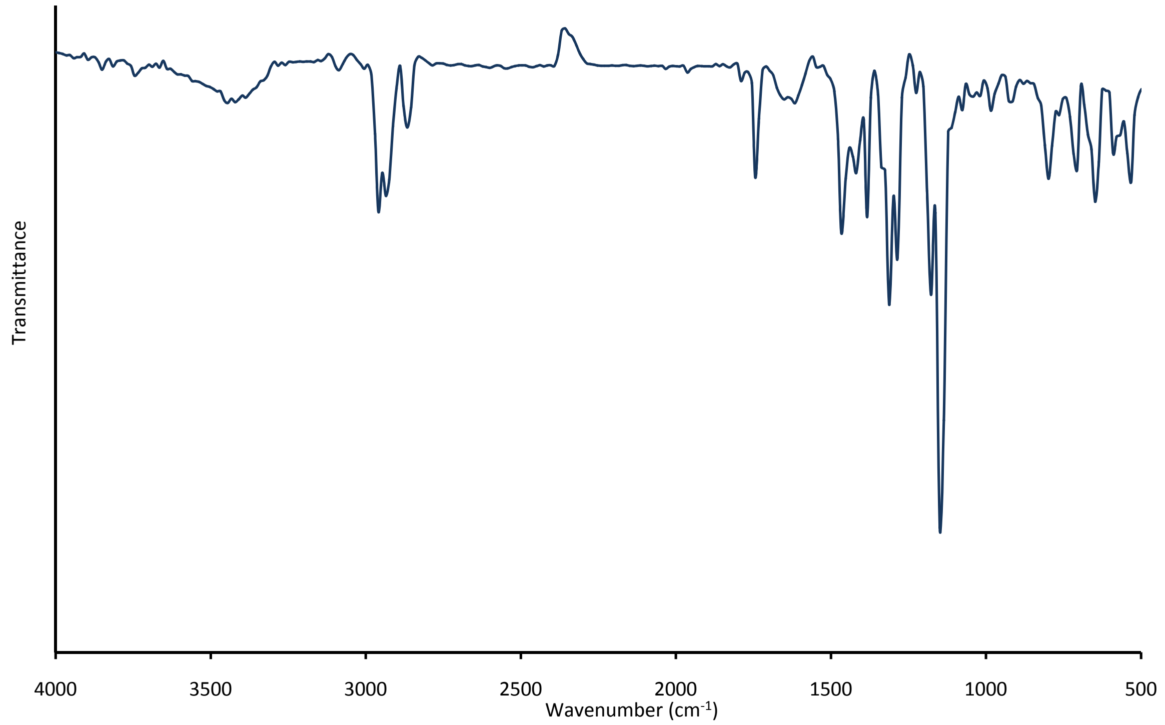

Figure S34: IR spectrum of **3b** (KBr).

- 1,2-Dicyano-4,5-bis(pentylsulfonyl)benzene **5**

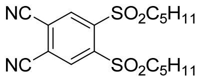

1,2-Dicyano-4,5-bis(pentylthio)benzene (**6**) (3 g, 9 mmol) was dissolved in 55 mL of acetic acid at 90 °C. A total amount of 43 mL of 33% H_2O_2 was added to the stirred solution as 5 mL portions every 15 min in the course of 2 h. The resulting turbid mixture was stirred at 90 °C for 2 h, until no **6** could be detected by TLC. After that, the reaction was stopped and allowed to cool to room temperature. The reaction mass was diluted with 100mL of brine, and the organic phase was extracted with 75 mL of dichloromethane (×2). Joined organic layer was washed with 150 mL of H_2O and 150 mL brine in a separation funnel. After that, it was dried over Na_2SO_4, filtered, rotary evaporated till dryness, affording a crude product as yellow oil. Column chromatography on silica gel with a mixture of dichloromethane-ethyl acetate (30:1, vol) yielded yellow-pale oil. Finally, the product was recrystallized from 40 mL of methanol, filtered, dried under vacuum.Compound **5** was obtained as a crystalline pale-yellow solid: 3.03 g (85%).

M. p.: 75 °C. ^{1}H NMR (300 MHz, $CDCl_3$): δ (ppm) = 8.68 (s, 2H), 3.69-3.63 (m, 2H; SO_2CH_2), 1.81-1.71 (m, 2H; $SO_2CH_2\mathbf{CH_2}$), 1.46-1.28 (m, 8H; $SO_2(CH_2)_2(\mathbf{CH_2})_2$), 0.92-0.87 (m, 6H; CH_3); ^{13}C NMR (75.5 MHz, $CDCl_3$): δ (ppm) = 144.61, 137.60, 121.29, 113.19, 57.26, 30.36, 29.83, 22.13, 13.77.

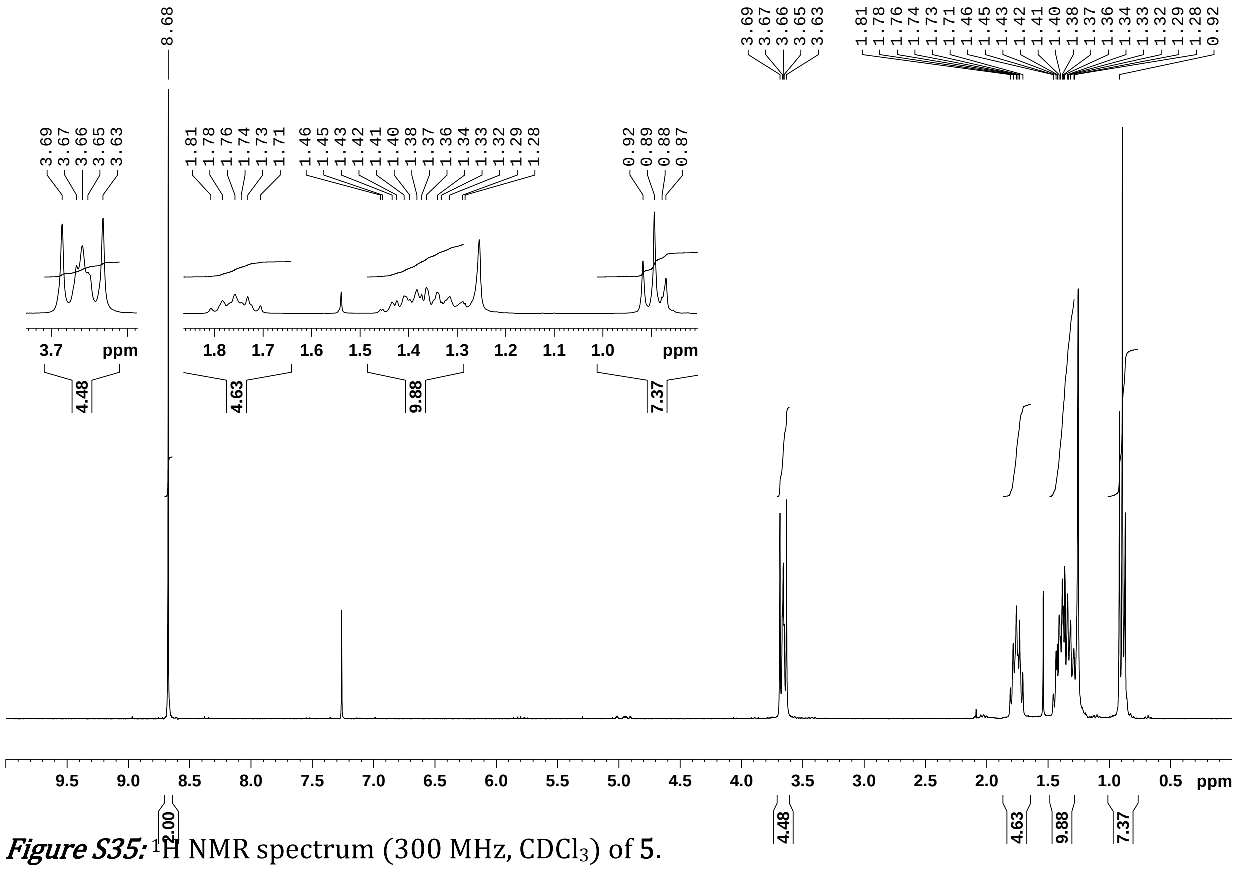

Figure S35: ^{1}H NMR spectrum (300 MHz, $CDCl_3$) of **5**.

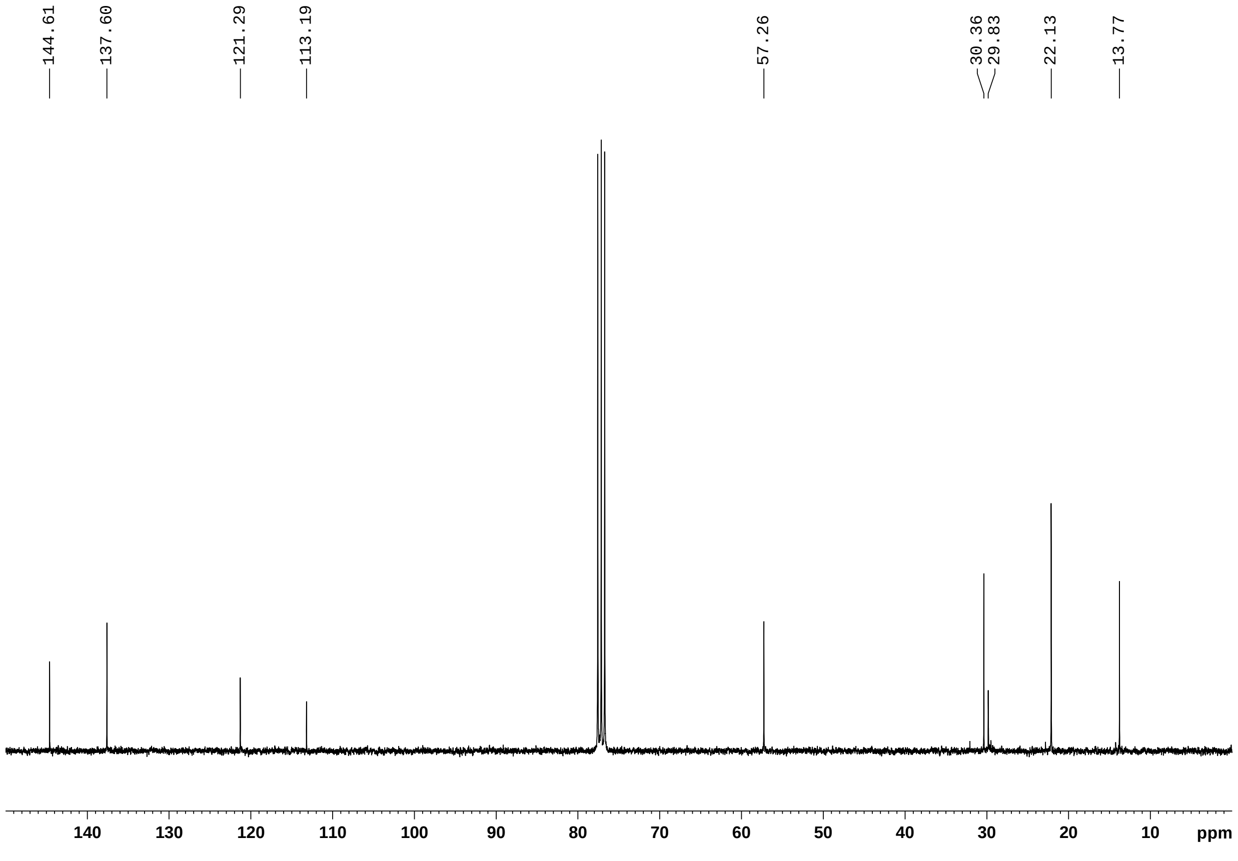

Figure S36: ^{13}C NMR spectrum (75 MHz, $CDCl_3$) of **5**.

- 1,2-Dicyano-4,5-bis(penylthio)benzene**6**

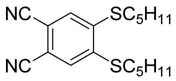

4,5-Dichloro-1,2-dicyanobenzene (**7**) (1 g, 5mmol), dry K_2CO_3 (2.0 g, 15mmol), and 10 mL of dimethylformamide (DMF) were placed in a 50-mL round-bottom flask, equipped with a magnetic stirrer, rubber seal, and globe. After that, 1-pentanethiol (1.5 mL, 12 mmol) was added via syringe, and the reaction was stirred at 100 ^{o}C for 4 hours when no **7** or a product of mono-substitution could be detected by TLC. Reaction mass was allowed to reach rt, and further poured into 50 mL of water. Formed precipitate was filtered, washed with water and air dried. Crude product was then dissolved in 50 mL of dichloromethane and filtered. Dichloromethane was rotary evaporated in a presence of celite till dryness. Resulting solid was used to charge the column filled with silica gel. The product was eluted with a mixture of hexane-ethylacetate (10:1, vol). After solvents were rotary evaporated, the product was recrystallized from methanol, filtered and vacuum dried. **6**was obtained as a white crystalline solid: 1.5 g (85%).

Mp: 64 °C. ^{1}H NMR (300 MHz, $CDCl_3$): δ (ppm) = 7.42(s, 2H), 3.01-2.99 (m, 2H; SO_2CH_2), 1.78-1.73 (m, 2H; $SO_2CH_2\mathbf{CH_2}$), 1.48-1.39 (m, 8H; $SO_2(CH_2)_2(\mathbf{CH_2})_2$), 0.94-0.89 (m, 6H; CH_3); ^{13}C NMR (75.5 MHz, $CDCl_3$): δ (ppm) = 144.39, 128.29, 115.81, 111.22, 32.85, 31.14, 27.90, 22.32, 14.00.

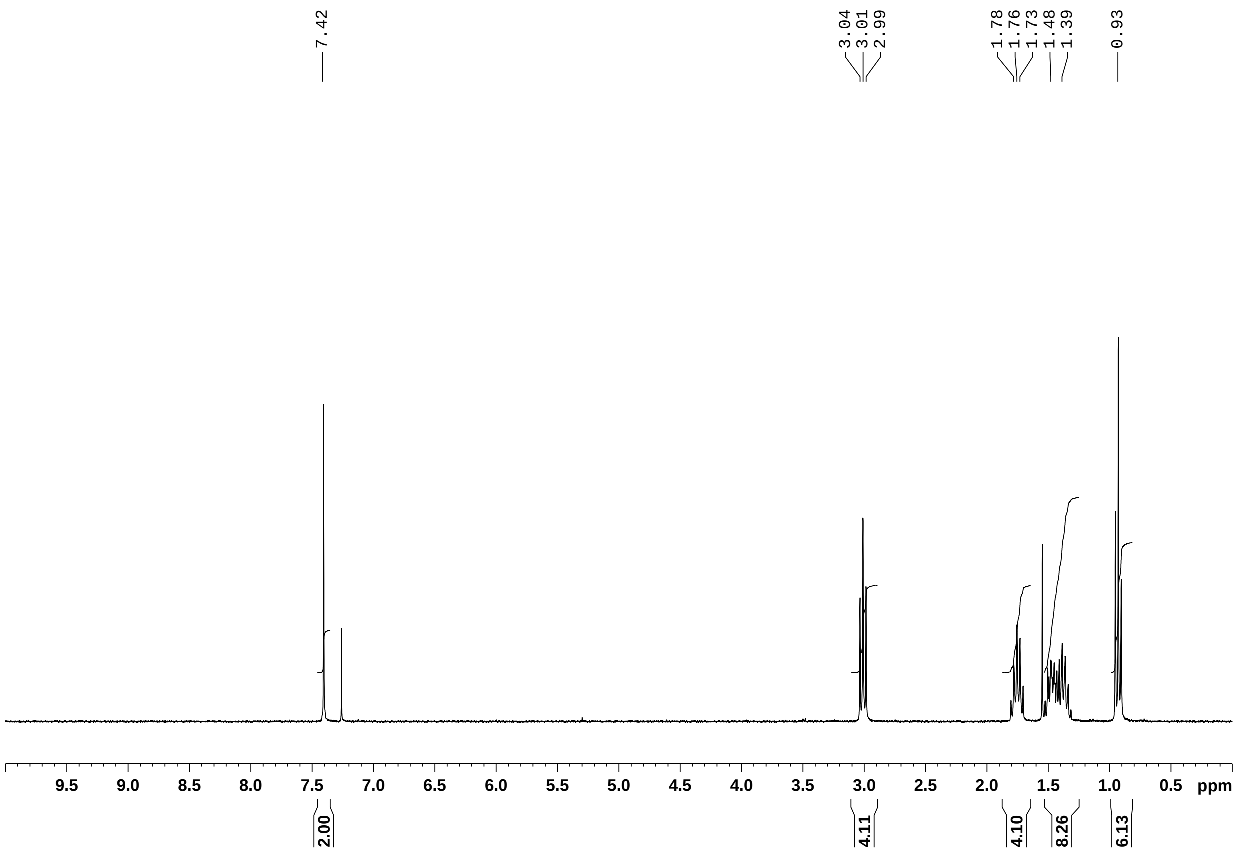

Figure S37: ^{1}H NMR spectrum (300 MHz, $CDCl_3$) of **6**.

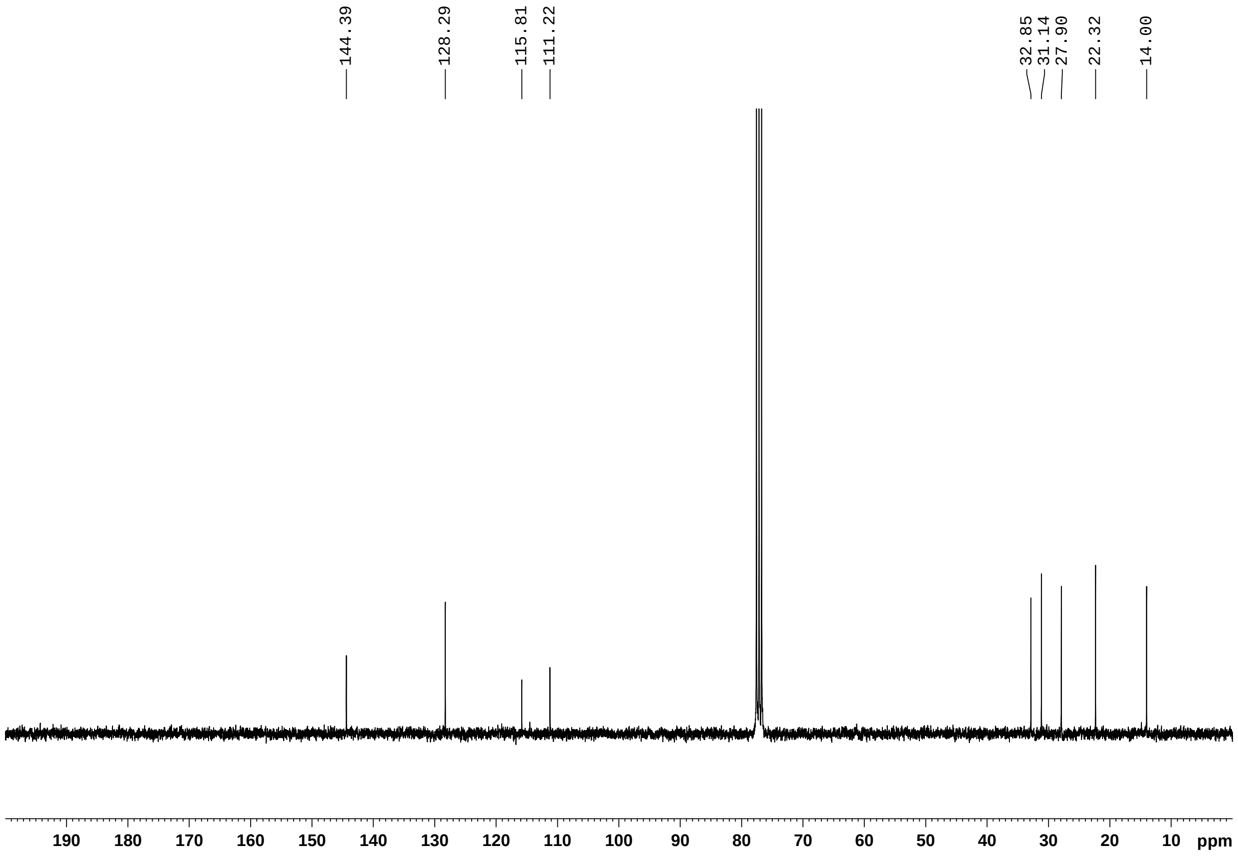

Figure S38: ^{13}C NMR spectrum (75 MHz, $CDCl_3$) of **6**.

3. Electrochemistry

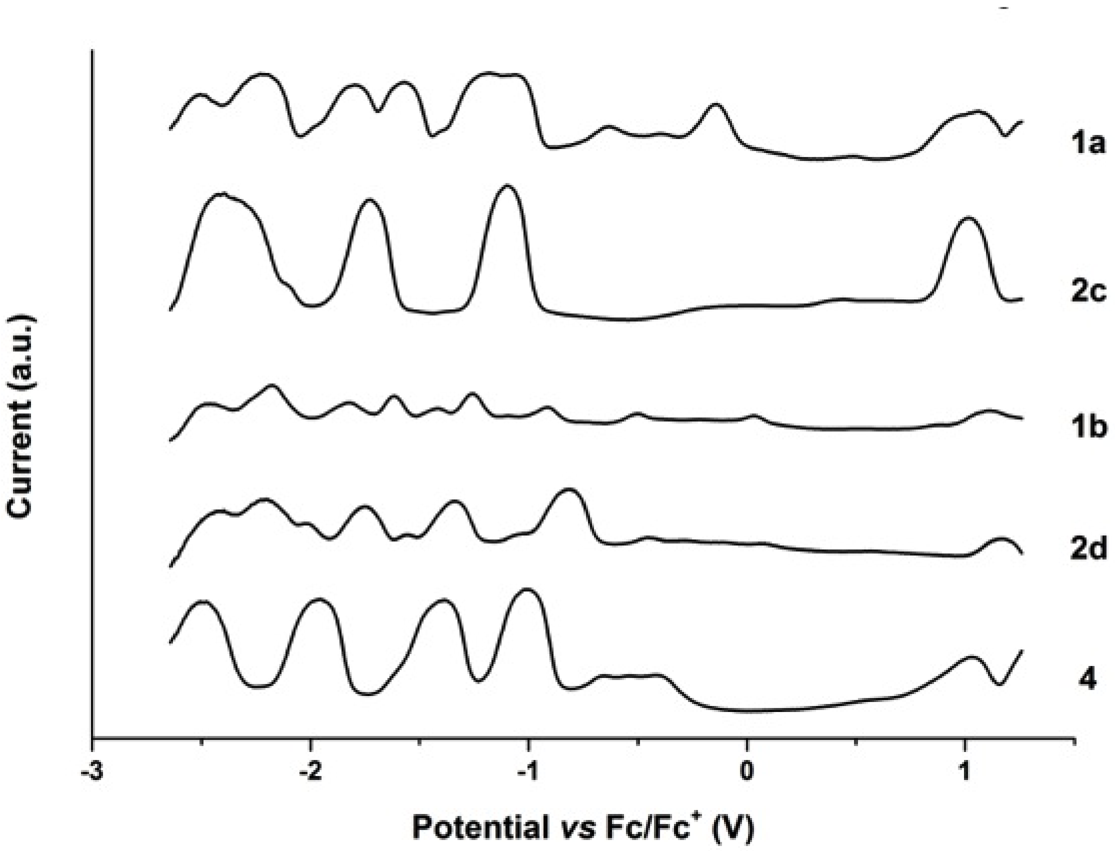

*Figure S39:*Differencial pulsevoltammograms of 1a, 2c, 1b, 2dand 4(≈10^{-3}M)at 20 mVs^{-1}in*o*-dichlorobenzene with 0.05 M (*n*-Bu)$_4$NPF$_6$ as a supporting electrolyte, carbon glassy as working electrode, Ptwire as counter electrode, Ag wire as pseudo-reference electrode. Potential values are referred to $E^{ox}_{1/2}$ of the Fc/Fc+ redox couple (+ 0.24 V in the present conditions). Decomposition products of 1a and 1b reveal reductions and oxidations at -0.63, -0.14 V and -0.50, 0.03 V, respectively.

4. Time resolved absorption

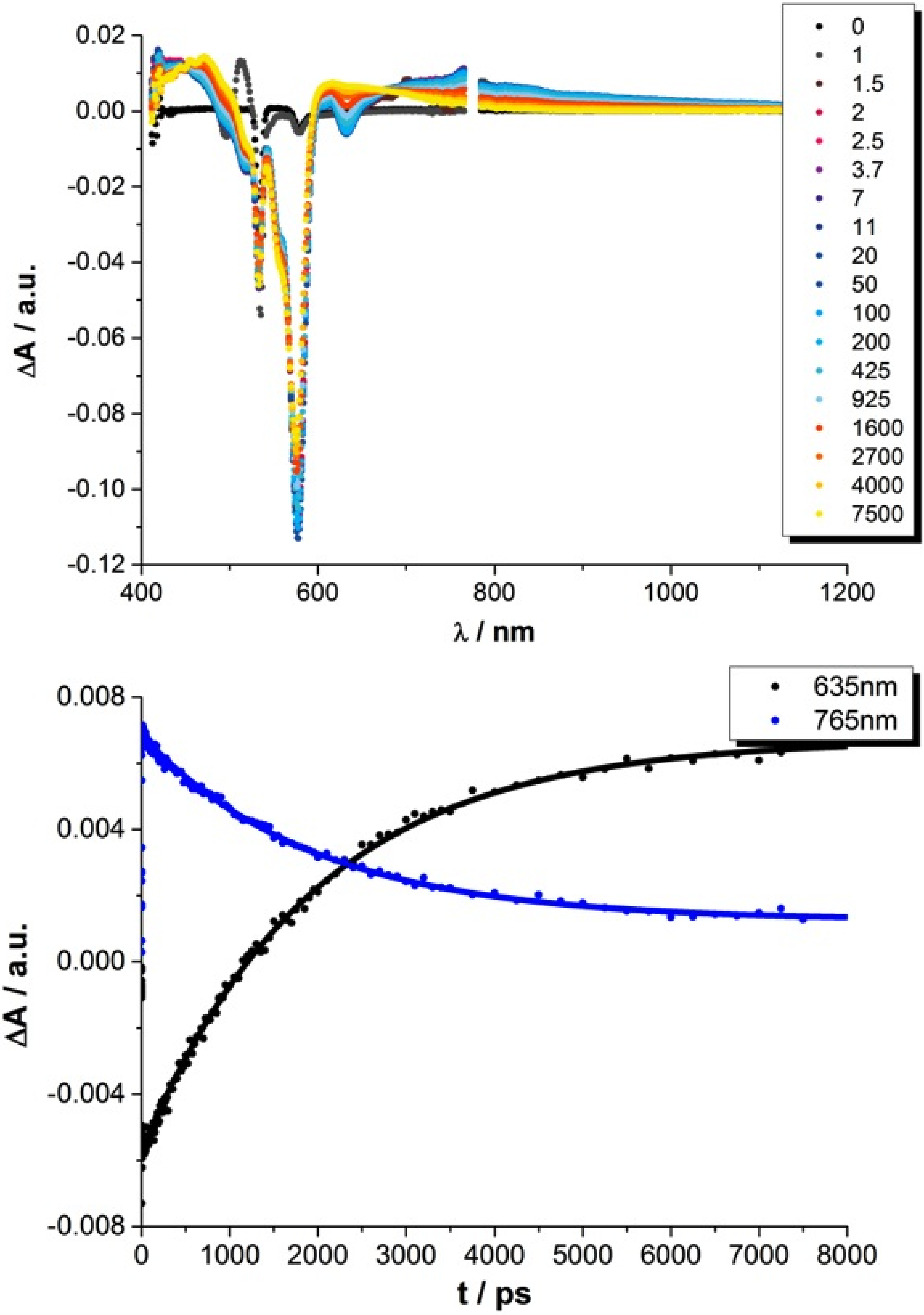

Figure S40. (top) Differential absorption spectra (visible and near-infrared) obtained upon femtosecond flash photolysis (530 nm) of **2c** (10^{-5} M) in argon-saturated toluene with several time delays between 0 and 7500 ps at room temperature. (bottom) Time-absorption profiles of the spectra shown at the top at 635 and 765 nm monitoring the intersystem crossing.

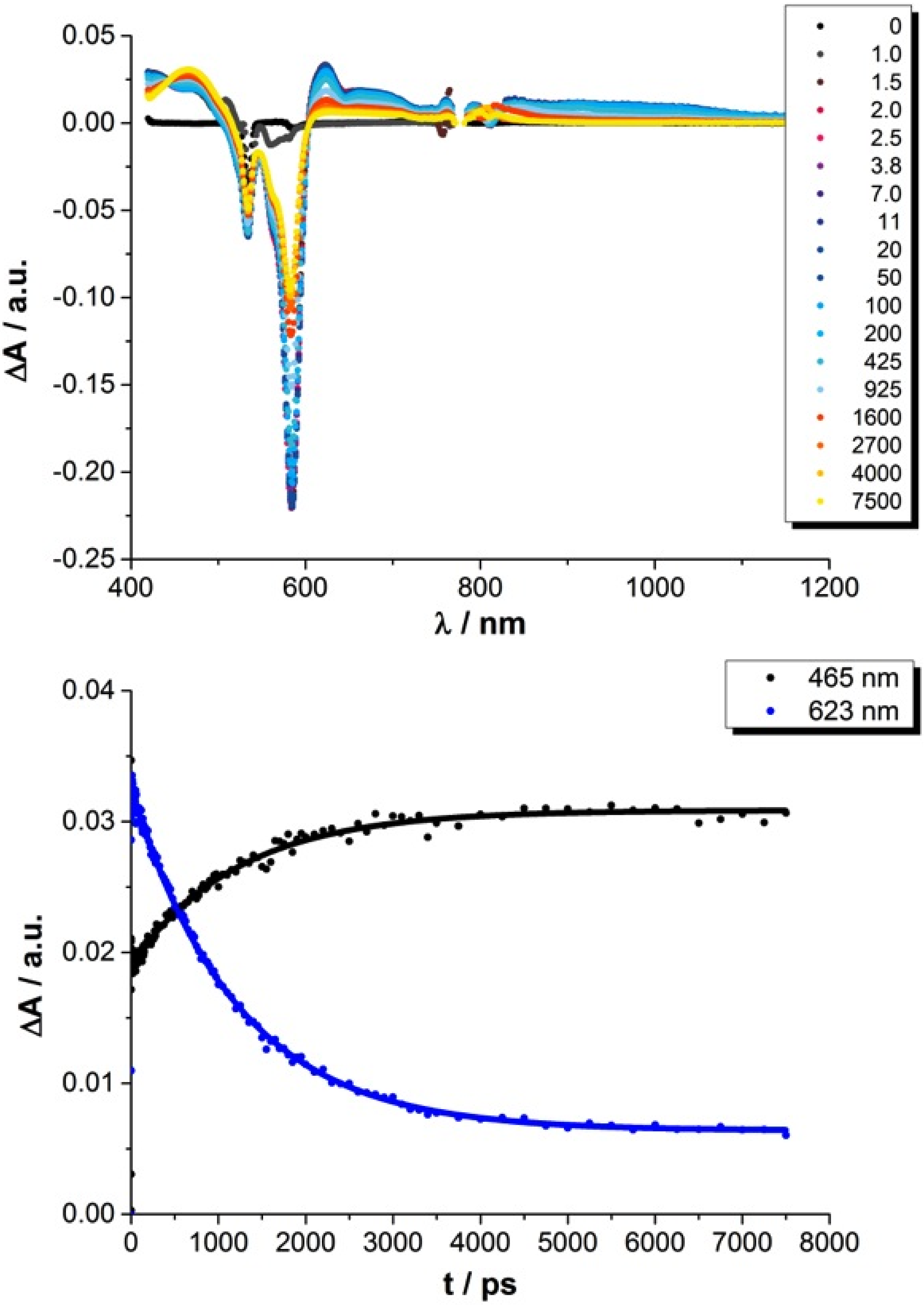

Figure S41. (top) Differential absorption spectra (visible and near-infrared) obtained upon femtosecond flash photolysis (530 nm) of **2d** (10^{-5} M) in argon-saturated toluene with several time delays between 0 and 7500 ps at room temperature. (bottom) Time-absorption profiles of the spectra shown at the top at 465 and 623 nm monitoring the intersystem crossing.

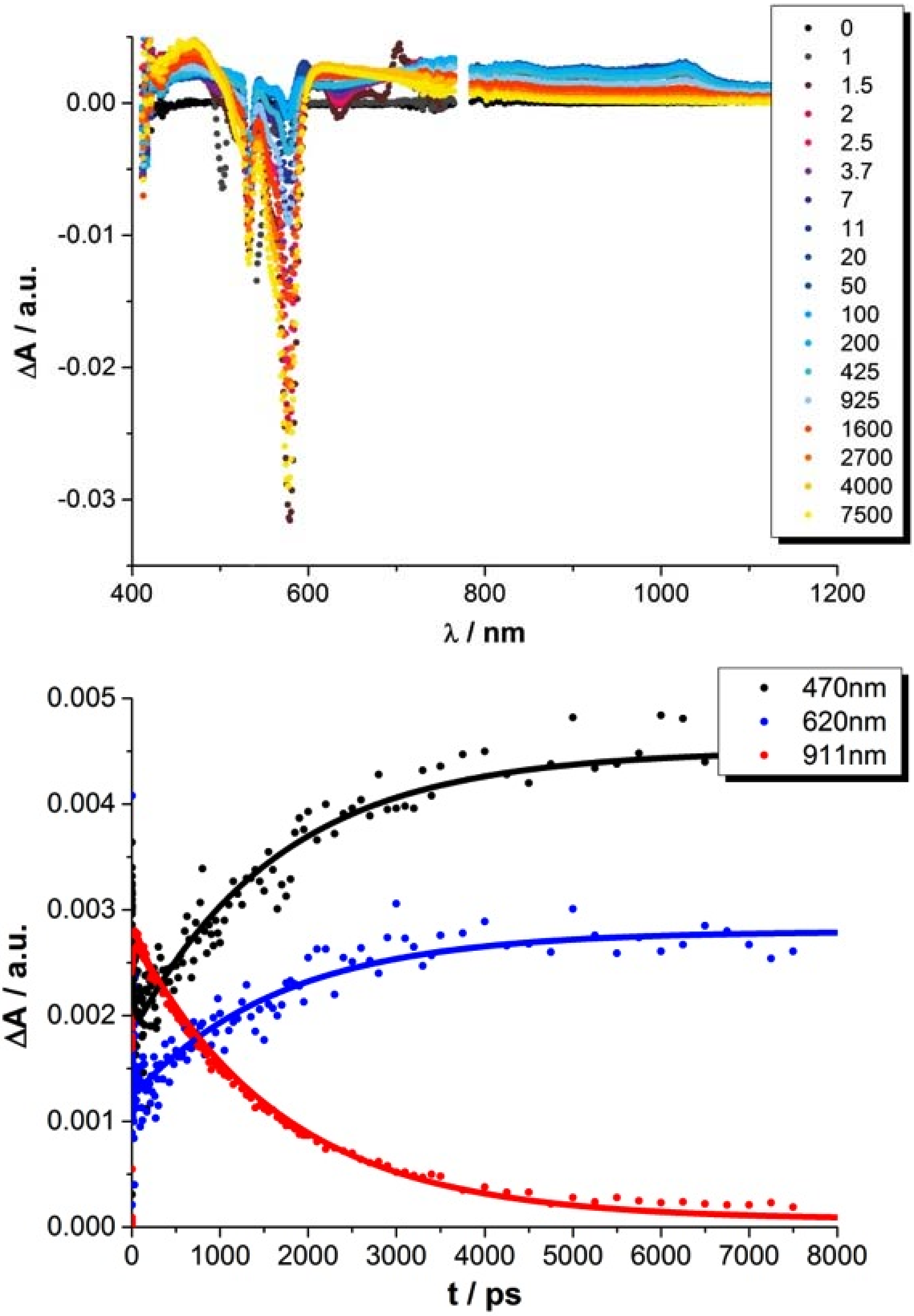

Figure S42. (top) Differential absorption spectra (visible and near-infrared) obtained upon femtosecond flash photolysis (530 nm) of **1a** (10^{-5} M) in argon-saturated toluene with several time delays between 0 and 7500 ps at room temperature. (bottom) Time-absorption profiles of the spectra shown at the top at 470, 620, and 911 nm monitoring the energy transfer.

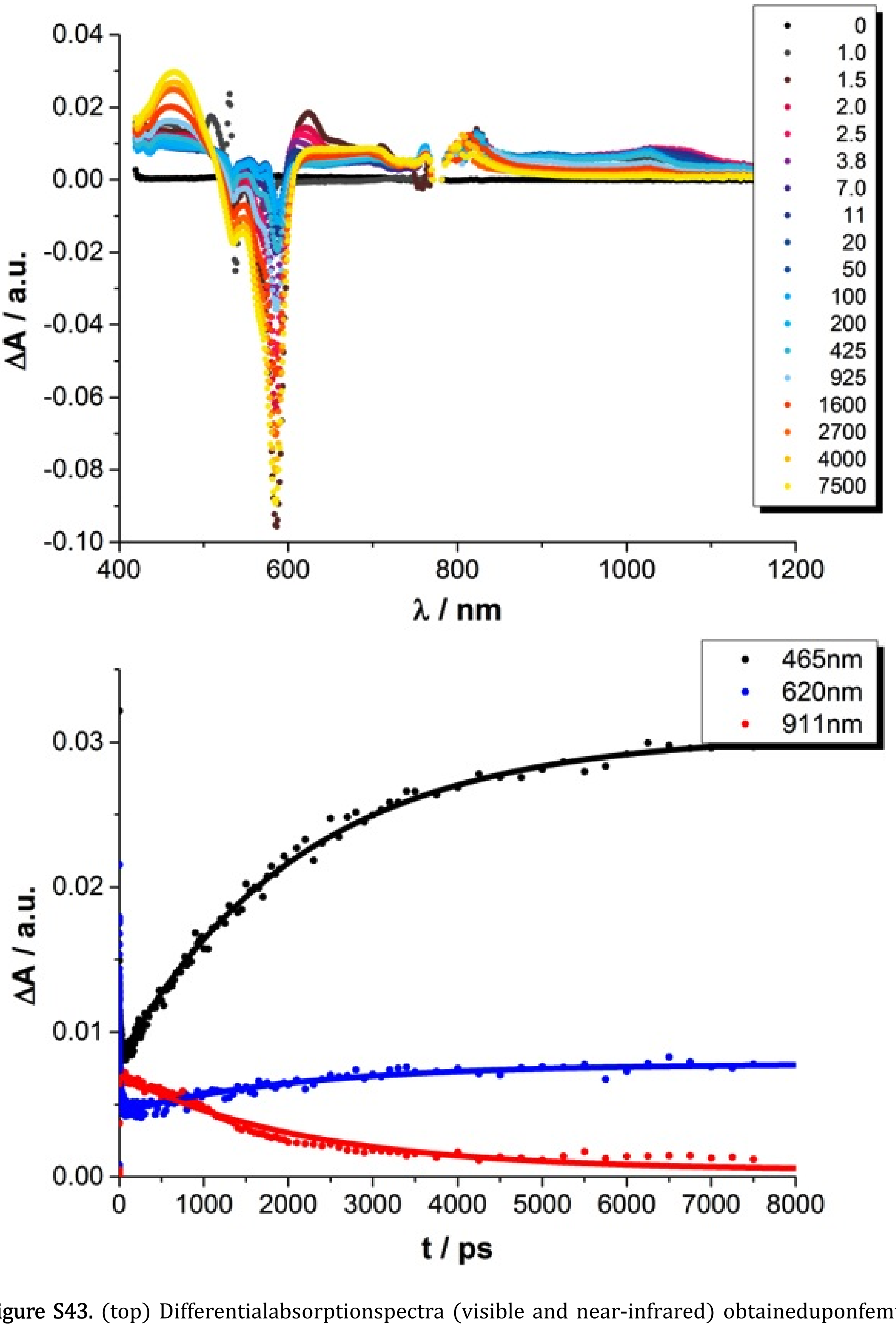

Figure S43. (top) Differentialabsorptionspectra (visible and near-infrared) obtaineduponfemtosecond flash photolysis (530 nm) of **1b** (10^{-5} M) in argon-saturatedtoluenewithseveral time delaysbetween 0 and 7500 ps at roomtemperature. (bottom) Time-absorptionprofiles of thespectrashown at the top at 465, 620, and 911 nmmonitoringtheenergy transfer.

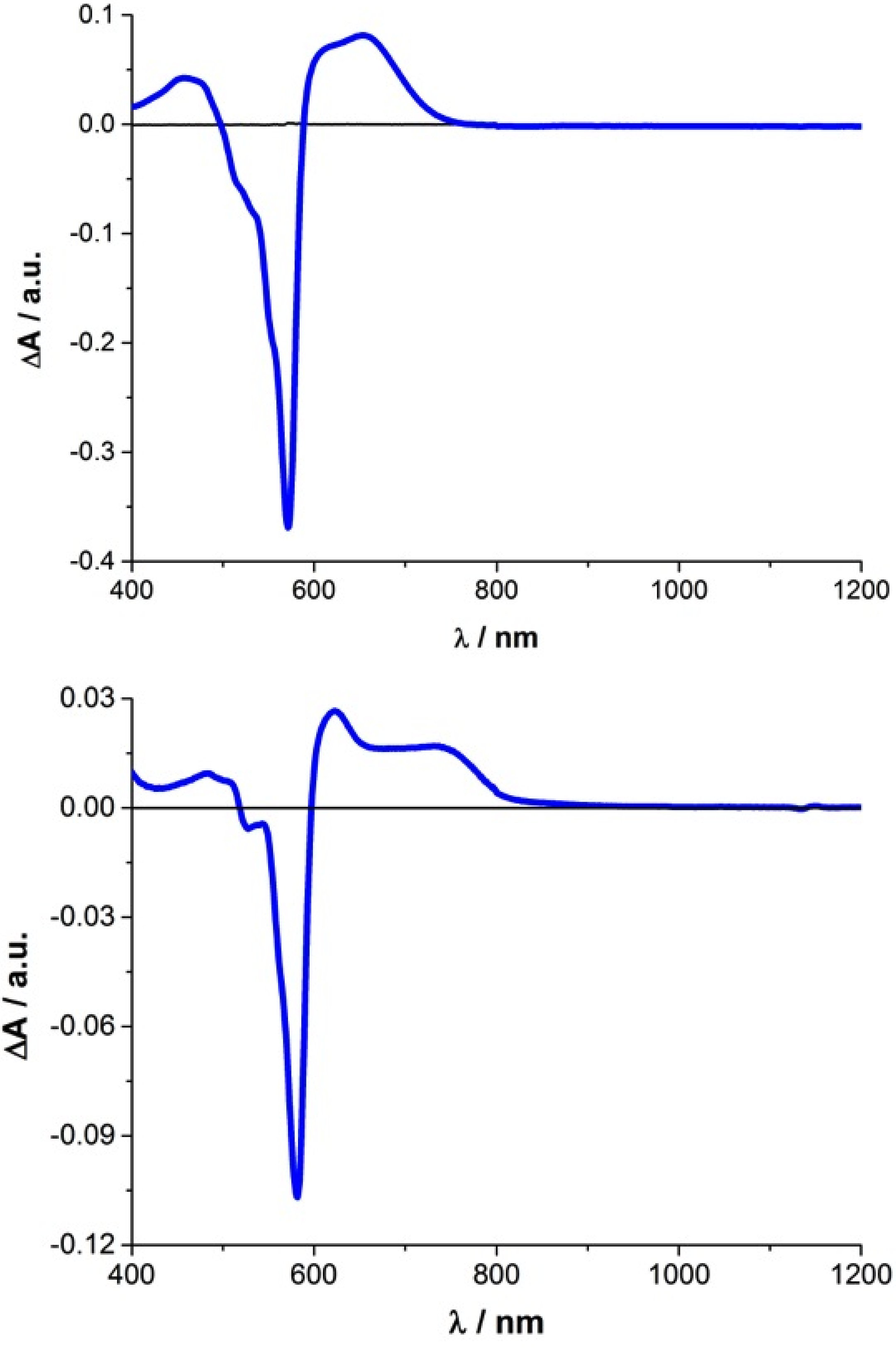

Figure S44. Differential absorption spectra (visible and near infrared) obtained upon electrochemical reduction of **2c** (top) and **2d** (bottom) at an applied bias of −0.5 and −0.3 V in argon-saturated toluene/acetonitrile mixtures (4/1 v/v) at room temperature.

5. Crystallographic data for 2d

A clear purple plate-like specimen of $C_{59}H_{79}BClN_6O_{13}S_6$, approximate dimensions 0.02 mm x 0.20 mm x 0.27 mm, was used for the X-ray crystallographic analysis. The X-ray intensity data were measured.

A total of 1060 frames were collected in a Bruker Kappa Apex II diffractometer. The total exposure time was 26.50 hours. The frames were integrated with the Bruker SAINT Software package using a narrow-frame algorithm. The integration of the data using a triclinic unit cell yielded a total of 59605 reflections to a maximum θ angle of 20.83° (1.00 Å resolution), of which 14024 were independent (average redundancy 4.250, completeness = 98.6%, R_{int} = 7.66%, R_{sig} = 6.96%) and 9016 (64.29%) were greater than $2\sigma(F^2)$. The final cell constants of *a* = 17.809(2) Å, *b* = 18.594(2) Å, *c* = 22.412(3) Å, α = 73.894(4)°, β = 83.175(4)°, γ = 71.769(3)°, volume = 6768.1(14) $Å^3$, are based upon the refinement of the XYZ-centroids of 8648 reflections above 20 σ(I) with 4.664° < 2θ< 42.41°. Data were corrected for absorption effects using the multi-scan method (SADABS). The ratio of minimum to maximum apparent transmission was 0.758.

The structure was solved and refined using the Bruker SHELXTL Software Package, using the space group P -1, with Z = 4 for the formula unit, $C_{60}H_{77}BN_6O_{13}S_6\cdot(CH_2Cl_2)_{0.25}$. The final anisotropic full-matrix least-squares refinement on F^2 with 1540 variables converged at R_1 = 10.80%, for the observed data and wR_2 = 36.55% for all data. The goodness-of-fit was 1.315. The largest peak in the final difference electron density synthesis was 3.545 $e^-/Å^3$ and the largest hole was -0.555 $e^-/Å^3$ with an RMS deviation of 0.156 $e^-/Å^3$.

Table S1. Sample and crystal data for **2d**.

Chemical formula	$C_{60}H_{77}BN_6O_{13}S_6\cdot(CH_2Cl_2)_{0.25}$
Temperature	100(2) K
Wavelength	0.71073 Å
Crystalsize	0.02 x 0.20 x 0.27 mm
Crystalhabit	clearpurpleplate
Crystalsystem	triclinic
Spacegroup	P -1
Unitcelldimensions	*a* = 17.809(2) Å α = 73.894(4)°
	b = 18.594(2) Å β = 83.175(4)°
	c = 22.412(3) Å γ = 71.769(3)°
Volume	6768.1(14) $Å^3$
Z	4
Absorptioncoefficient	0.304 mm^{-1}

Table S2. Data collection and structure refinement for **2d.**

**Theta range for data collection**	1.93 to 20.83°	
**Indexranges**	-17<=h<=17, -18<=k<=18, -20<=l<=22	
**Reflectionscollected**	59605	
**Independentreflections**	14024 [R(int) = 0.0766]	
**Coverage of independentreflections**	98.6%	
**Absorptioncorrection**	multi-scan	
**Structuresolutiontechnique**	directmethods	
**Structuresolutionprogram**	SHELXS-97 (Sheldrick, 2008)	
**Refinementmethod**	Full-matrix least-squares on F^2	
**Refinementprogram**	SHELXL-97 (Sheldrick, 2008)	
**Functionminimized**	$\Sigma\ w(F_o^2 - F_c^2)^2$	
**Data / restraints / parameters**	14024 / 5 / 1540	
**Goodness-of-fit on F^2**	1.315	
**Final R indices**	9016 data; $I>2\sigma(I)$	$R_1 = 0.1080$, $wR_2 = 0.2951$
	all data	$R_1 = 0.1661$, $wR_2 = 0.3655$
**Weightingscheme**	$w=1/[\sigma^2(F_o^2)+(0.2200P)^2+0.5000P]$ where $P=(F_o^2+2F_c^2)/3$	
**Largest diff. peak and hole**	3.545 and -0.555 eÅ^{-3}	
**R.M.S. deviation from mean**	0.156 eÅ^{-3}	

In **2d**, thesixelectronegativepentylsulfonylgroupsaretheinceptiontointermolecularrepulsionsandthe lack oftypicalSubPcpacking in a singlecrystal.[8] The presenceoftwoSubPcs in theasymmetricunitcellresulted in itsrather large structuralparameters (Table S1,S2). Figure S46demonstratesthat van der Waals interactionsbetweenoxygenatomsofthepentylsulfonylsandthecoresofadjacentSubPcsdictatethepacking. It is important to note that most of the alkyl chains present a high degree of disorder. Half of them (C25-C29, C45-C49, C50-C54, C85-C89, C90-C95, C95, C99 and C100-C104) could be refined anisotropically, although in some cases the occupancy factor needed to be lowered to values than 1 in order to get a good agreement. This means that only one of the multiple sites that these carbon occupy could be located (the one with most probability associated). In other cases, the final carbon atom did not allow the anisotropic refinement (C34, C44) and had to be refined with an isotropical displacement parameter. In a few cases, a larger portion of the chain needed to be kept isotropically refined (atoms C38-C39 and C111-C114). Only in two cases (C109 and C111), two alternate positions for the carbon atoms were located and a statistical disorder model was made. There are some unassigned density peaks, which are attributed to other solvent molecules that could not be identified.

The large number of atoms in the asymmetric unit, the quality of the collected data and the high degree of disorder expected for the alkyl substituted compounds prevented us to establish a more accurate model.

Structural data for compound **2d** have been deposited at the Cambridge Crystallographic Data Centre with deposition number CCDC 1014106.

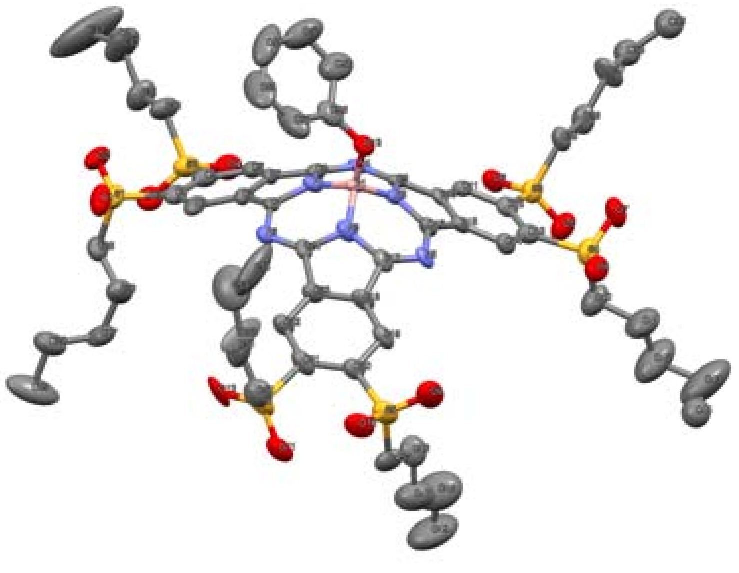

Figure S45. Ellipsoid plot of one of the molecules from the asymmetric unit in compound **2d** (ellipsoids at 50% probability). Hydrogen atoms have been omitted for clarity.

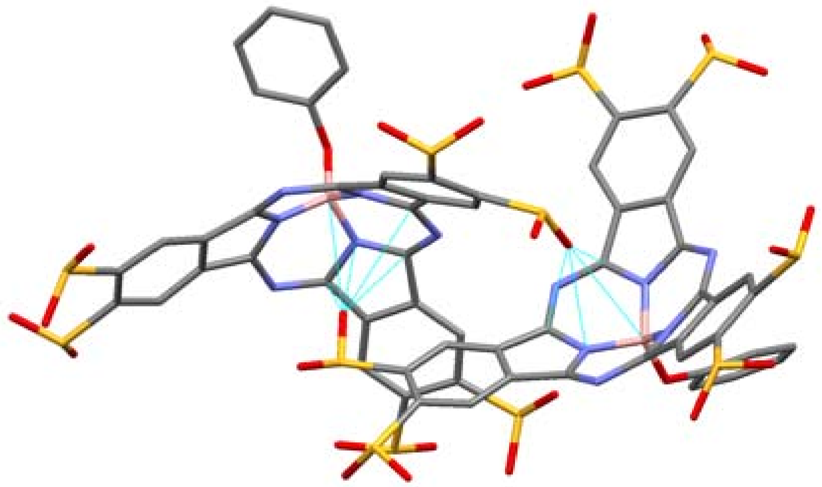

Figure S46. Simplifiedviewofthe cores of two interacting adjacent molecules of **2d**. Hydrogen atomsareomittedforclarity.

6. References

[1] a) J. Guilleme, D. Gonzalez-Rodriguez, T. Torres, *Angew. Chem. Int. Ed.*2011, *50*, 3506-3509; b) G. E. Morse, M. G. Helander, J. F. Maka, Zh.-H. Lu, T. P. Bender, *ACS Appl. Mat. Int.*2010, *2*, 1934-1944; c) M.E. El-Khouly, J. B. Ryu, K.-Y. Kay, O. Ito, S. Fukuzumi, *J. Phys. Chem. C*2009, *113*, 15444-15453; d) D. Gonzalez-Rodriguez, T. Torres, M. M. Olmstead, J. Rivera, M. A. Herranz, L. Echegoyen, C. AtienzaCastellanos, D. M. Guldi, *J. Am. Chem. Soc.*2006, *128*, 10680-10681; e) C. G. Claessens, T. Torres, *Angew. Chem. Int. Ed.*2002, *41*, 2561-2565.

[2] C. G. Claessens, D. Gonzalez- Rodriguez, B. delRey, T. Torres, G. Mark, H.-P. Schuchmann, C. vonSonntag, J. G. MacDonald, R. S. Nohr, *Eur. J. Org. Chem.* 2003, 2547-2551.

[3] M. Prato, M. Maggini, C. Giacometti, G. Scorrano, G. Sandona, G. Farnia, *Tetrahedron*1996, *52*, 5221-5234.

[4] D. Wohrle, M. Eskes, K. Shigehara, A. Yamada, *Synthesis*1993, 194-196.

[5] D. Gonzalez-Rodriguez, T. Torres, D. M. Guldi, J. Rivera, M. A. Herranz, L. Echegoyen, *J. Am. Chem. Soc.*2004, *126*, 6301-6313.

[6] M. Ince, A. Medina, J.-H. Yum, A. Yella, C. G. Claessens, M. V. Martinez-Diaz, M. Graetzel, M. K. Nazeeruddin, T. Torres, *Chem. Eur. J.*2014, *20*, 2016-2021.

[7] K. Okusa, H. Tanaka, M. Ohira, *J. Chromatogr. A*2000, *869*, 143-149.

[8] a) J. S. Castrucci, M. G. Helander, G. E. Morse, Z.-H. Lu, C. M. Yip, T. P. Bender, *Cryst. Growth Des.*2012, 12, 1095-1100; b) G. E. Morse, M. G. Helander, J. F. Maka, Z.-H. Lu, T. P. Bender, *Appl. Mater. Interfaces*2010, *2*, 1934-1944.
